# Amphipathic barbiturates as marine product mimics with cytolytic and immunogenic effects on head and neck squamous cell carcinoma cell lines

**DOI:** 10.3389/fphar.2023.1141669

**Published:** 2023-03-30

**Authors:** Susannah von Hofsten, Manuel K. Langer, Katja Korelin, Synnøve Magnussen, Dominik Ausbacher, Trude Anderssen, Tuula Salo, Morten B. Strøm, Annette Bayer, Ahmed Al-Samadi, Gerd Berge

**Affiliations:** ^1^ Department of Medical Biology, Faculty of Health Sciences, UiT—The Arctic University of Norway, Tromsø, Norway; ^2^ Department of Chemistry, Faculty of Science and Technology, UiT—The Arctic University of Norway, Tromsø, Norway; ^3^ Department of Oral and Maxillofacial Diseases, University of Helsinki, Helsinki, Finland; ^4^ Translational Immunology Research Program (TRIMM), University of Helsinki, Helsinki, Finland; ^5^ Department of Pharmacy, Faculty of Health Sciences, UiT—The Arctic University of Norway, Tromsø, Norway

**Keywords:** cancer, lysosomotropic, calreticulin, immunogenic cell death, head and neck squamous cell cancer (HNSCC), drug development

## Abstract

The incidence of head and neck squamous cell carcinoma (HNSCC) is increasing and the conventional treatments for this form of cancer can be tough. Despite the success of existing immunotherapies in some HNSCC patients, many do not respond to this type of treatment. Thus, the development of novel anti-cancer therapies should be prioritized. In the current study, the anticancer activity of a panel of novel compounds, herein termed marine product mimics (MPMs), against HNSCC cell lines is explored. The previously reported compound MPM-1, which is structurally related to the novel MPMs, was shown to have promising effects on the HNSCC cell line HSC-3. The results from the current study indicate that the novel MPMs are more potent than MPM-1 but cause a similar type of cell death. The results indicated that the MPMs must cross through the cell membrane to exert their action and that they are lysosomotropic. Further experiments showed that some of the MPMs could induce phosphorylation of eukaryotic initiation factor 2α (eIF2α) in HSC-3 and UT-SCC-24A cells, which indicates that they can activate the integrated stress response that is strongly associated with immunogenic cell death. Cell surface expression of calreticulin and release of HMGB1 and ATP, which are all hallmarks of immunogenic cell death, was also demonstrated in HSC-3 and UT-SCC-24A cells treated with MPMs. This suggests that the MPMs are interesting candidates for future HNSCC cancer therapies.

## 1 Introduction

Head and neck squamous cell carcinoma (HNSCC) (which includes all cancers of the lip and oral cavity, salivary glands, oropharynx, nasopharynx, hypopharynx, and larynx) is the cancer with the seventh highest incidence worldwide, and the incidence is increasing (Sung et al., 2021). Standard treatment of HNSCC involves surgery and irradiation or chemoradiotherapy with cisplatin and 5-fluorouracil (Johnson et al., 2020). The epidermal growth factor receptor (EGFR) inhibitor cetuximab can also be used. However, resistance to chemotherapy and targeted therapy is a frequent concern in HNSCC patients (Wang et al., 2016). During recent years, immunotherapy in the form of programmed cell death protein 1 (PD-1) inhibitors has also been approved as treatment for patients with recurrent or metastatic HNSCC in several countries (Johnson et al., 2020).

The emergence of different forms of immunotherapy has revolutionized cancer treatment during recent years.In particular, checkpoint inhibitors have been very successful in certain cancers and patient groups (Waldman et al., 2020). Nevertheless, there are still many patients who do not respond to treatment with existing immunotherapies. Thus, the need for the development of novel therapies persists.

The effect of immunotherapy relies on the ability of the patient’s own immune system to recognize and kill cancer cells. The recognition of cancer cells is dependent on the cancer cells’ presentation of cancer-related antigens on major histocompatibility complex molecules (MHC) (Waldman et al., 2020). Typically, these are neoantigens which emerge due to the mutations that occur in cancer cells during tumor progression. Thus, cancer types with a high mutational burden are often those that respond best to immunotherapy (Sha et al., 2020). HNSCC generally has a medium to high tumor mutational burden and studies have indicated that those HNSCC patients who have a higher tumor mutational burden do respond better to immunotherapy than those with a lower mutational burden (Sha et al., 2020).

A high mutational burden is not only beneficial for treatment with immune checkpoint inhibitors, but also for other immunotherapies. A promising form of immunotherapy for solid tumors consists of the intratumoral injection of oncolytic compounds that induce immunogenic cell death in cancer cells (Vitale et al., 2021). The induction of immunogenic cell death causes the release of tumor antigens as well as damage-associated molecular patterns (DAMPs) such as high mobility group box 1 (HMGB1) and adenosine triphosphate (ATP) (Galluzzi et al., 2017). Cell surface expression of calreticulin, which functions as an “eat-me” signal for immune cells, is also considered a hallmark of immunogenic cell death. Cancer cells succumbing to immunogenic cell death can cause the activation of an anti-tumor immune response, which leads to the infiltration of immune cells to the tumor area and the killing of more cancer cells.

We have previously reported on the cytolytic marine natural product mimic MPM-1 (von Hofsten et al., 2022). MPM-1 is a simplified mimic of the natural product *eusynstyelamide D*, which has been isolated from an arctic bryozoan (Tadesse et al., 2011). Instead of the five-membered dihydroxybutyrolactam ring which serves as the scaffold in *eusynstyelamide D*, MPM-1 is built on a symmetrical barbiturate scaffold, which makes it easier to synthesize. In addition, both *eusynstyelamide D* and MPM-1 contain two cationic and two lipophilic groups, which makes these compounds amphipathic. Our previous study suggested that MPM-1 was able to induce immunogenic cell death in the HNSCC cell line HSC-3 (von Hofsten et al., 2022). In the present study, we explore the anti-cancer and immunogenic effects of an extended panel of compounds which are structurally related to MPM-1. We study the mechanism of action of these compounds as well as the mode of death that they induce in HNSCC cell lines.

## 2 Materials and methods

### 2.1 Compounds

The MPMs were manufactured in-house and dissolved in DMSO to a concentration of 100 mM. Further dilutions were performed in cell culture media. The synthesis of the MPMs is described in detail in the Supplementary Data.S1

### 2.2 Cell culture

HSC-3 (RRID: CVCL_1288) was obtained from the Japanese Collection of Research Bioresources Cell Bank (JCRB Cell Bank, Osaka, Japan). The UT-SCC-8, UT-SCC-24A, UT-SCC-24B, UT-SCC-42A, UT-SCC-42B and UT-SCC-106A cell lines were all established at and obtained from Turku University Hospital and Prof. Grénman’s laboratory. All cancer cell lines were cultured in 1:1 DMEM/F-12 (#31330-038, Thermo Fisher Scientific, Waltham, MA, United States) supplemented with 10% fetal bovine serum (FBS), 1% ascorbic acid, 1% PenStrep, 0.1% amphotericin B and 0.01% hydrocortisone. The NOF cell line had been established in a previous study (Sinha et al., 2020). They were cultured in DMEM high glucose (#41965-039, Thermo Fisher Scientific) supplemented with 10% FBS, 1% sodium pyruvate, 1% PenStrep, and 0.1% amphotericin B. In the high throughput drug screening, the live cell apoptosis assay, and the migration and invasion assays, wells were plated with the human tumor-derived matrix “myogel” to provide a more realistic tumor microenvironment and improve the predictability of drug testing (Salo et al., 2018; Tuomainen et al., 2019). The use of human leiomyoma tissue to produce myogel was approved by the Ethics Committee of both Oulu and Helsinki University Hospitals (statement number 2/2017), and all research was performed in accordance with relevant regulations.

### 2.3 Hemolysis assay

The hemolytic effect of the MPMs was determined using a hemolysis assay as previously described (Paulsen et al., 2021). Briefly, hemolysis was determined using a heparinized fraction (10 IU/mL) of freshly drawn blood. The blood collected in ethylenediaminetetraacetic acid-containing test tubes (Vacutest, KIMA, Arzergrande, Italy) was used for the determination of the hematocrit (hct). The heparinized blood was washed 3 × with pre-warmed phosphate-buffered saline (PBS) and adjusted to a final hct of 4%. MPMs in DMSO (50 mM) were added to a 96-well polypropylene V-bottom plate (NUNC, Fisher Scientific, Oslo, Norway) and serially diluted. The test concentration range was 4–500 μM with DMSO contents ≤1%. A solution of 1% triton X-100 was used as a positive control for 100% hemolysis. As a negative control, a solution of 1% DMSO in PBS was included. Red blood cells (1% v/v final concentration) were added to the well plate and incubated at 37°C and 800 rpm for 1 h. After centrifugation (5 min, 3000 g), 100 μL from each well was transferred to a 96-well flat-bottomed microtiter plate, and absorbance was measured at 545 nm with a microplate reader (VersaMaxTM, Molecular Devices, Sunnyvale, CA, United States). The percentage of hemolysis was calculated as the ratio of the absorbance in the MPM treated and surfactant-treated samples, corrected for the PBS background. Three independent experiments were performed, and EC50 values are presented as averages.

### 2.4 High throughput drug screening

The MPMs were screened against a panel of eight different cell lines (HSC-3, UT-SCC-8, UT-SCC-24A, UT-SCC-24B, UT-SCC-42A, UT-SCC-42B and NOF) at the High Throughput Biomedicine Unit (HTB) at the Institute for Molecular Medicine Finland (FIMM). On day 1, black-walled 384-well plates were coated with 5 µl diluted myogel (0.5 mg/ml) per well using an automated reagent dispenser (MultiFlo FX, BioTek, Winooski, VT, United States). On day 2, 500 cells per well were seeded in 20 µl of complete media using the MultiFlo FX (BioTek), and the cells were left to adhere overnight. On day 3, the MPMs, cisplatin, DMSO and Benzethonium chloride were added to the cells using an automatic liquid handler (Echo 550, Labcyte Inc., San Jose, CA, United States). Cisplatin and the MPMs were added to yield five different concentrations in a ten-fold dilution series ranging from 10 to 100,000 nM. Each concentration was tested in triplicate wells. DMSO (0.1%) and benzethonium chloride (100 µM) served as negative and positive control, respectively. Three hours later, some of the plates were irradiated with 2 Gy in a gamma irradiator (OB29/4, STS, Braunschweig, Germany). All plates were taken to the irradiation room to ensure similar handling of the plates. 72 h later, the plates were cooled to room temperature before 25 µl of CellTiter-Glo 2.0 reagent (Promega, Madison, WI, USA) was added to each well using the MultiFlo FX (BioTek). Next, the plates were centrifuged for 5 min at 1,000 rpm before the luminescence signal was measured with the PHERAstar FS HT reader (BMG LABTECH GmbH, Ortenberg, Germany). The raw data was analyzed with the Breeze software (https://breeze.fmm.fi), which normalized the luminescence signal from treated cells against the signal from negative and positive control cells to calculate the percentage of inhibition and generate dose-response curves, which were then used to calculate drug sensitivity scores (DSS). The DSS is a parameter which combines the IC50, the slope of the dose-response curve, and the minimum and maximum responses into a single metric, as previously described (Yadav et al., 2014). Quality control was performed by calculation of the Z′-factor, which was >0.7 for all cell lines, indicating high quality of the assay (Zhang et al., 1999).

### 2.5 Flow cytometric detection of apoptosis

HSC-3 and UT-SCC-24A cells were seeded, 300,000 cells/well, in 6-well plates and left to adhere overnight. The media was then removed and replaced by 2 ml complete DMEM/F-12 containing 5, 7.5, 10 or 20 µM of MPM-2:0, MPM-6:0, MPM-3:2 or MPM-4:2, or 50 µM cisplatin. Some cells were left untreated. The cells were then incubated for 24 h. Next, the cell supernatants were collected before the cells were trypsinized and spun down with the supernatants. For the staining, an apoptosis detection kit was used (#88-8005-74, Thermo Fisher Scientific). The cells were washed once in PBS and once in 1x binding buffer before being incubated with FITC labeled Annexin V diluted 1:25 in 1 × binding buffer for 15 min. Next the cells were washed in 1 × binding buffer before being incubated with PI diluted 1:100 in 1 × binding buffer for at least 5 min before analysis was performed on the LSR Fortessa (BD Biosciences, CA, United States). Analyses were performed in FlowJo™ v.10 (https://www.flowjo.com/).

### 2.6 Live cell apoptosis assay

A black-walled 96-well plate (#6005182, PerkinElmer, Waltham, MA, United States) was coated with 50 µl myogel (0.5 mg/ml) per well. The next day, HSC-3 and UT-SCC-24A cells were stained with CellTrace Far Red (Invitrogen, Carlsbad, CA, USA) by resuspending 500,000 cells in 500 µl PBS and adding 0.5 µl of the dye before incubating the cells at 37°C for 20 min. Next, 2.5 ml of complete media was added to the cells, and they were incubated for another 5 min. The cells were then centrifuged and resuspended in complete media. The media remaining in the wells of the 96-well plate after myogel coating was removed and the cells were seeded with 1,000 cells/well in 100 µl of media. The cells were left to adhere overnight, and the following day, the media was replaced with 100 µl of media containing the Incucyte® caspase-3/7 green dye for apoptosis (Sartorius, Göttingen, Germany) (diluted 1:1,000), as well as 5 or 10 µM of the MPMs or 10 µM cisplatin. Control wells only contained media and the caspase-3/7 green dye. The plate was placed in the Incucyte S3 (Sartorius) and imaged at ×10 magnification every 2 hours for 24 h in total. Each condition had triplicate wells, and four images were taken per well. The experiment was conducted three times. The Incucyte software was used to analyze the area of red fluorescence (proliferation) and number of green and red objects (apoptotic cells).

### 2.7 MTS viability assays with bafilomycin A1 and z-VAD-FMK

HSC-3 and UT-SCC-24A cells were seeded, 10,000 cells/well, in flat bottom 96-well plates and left to adhere overnight. The supernatants were then removed and replaced by 50 µl of complete DMEM/F-12 ± 100 nM BafA1 (#196000, Sigma-Aldrich, St. Louis, MO, United States) or 50 µM z-VAD-FMK (#219007, Sigma-Aldrich). The plate was then incubated for 1 hour before another 50 µl of complete DMEM/F-12 ± 100 nM BafA1 or 50 µM z-VAD-FMK as well as the MPMs or cisplatin was added. The final concentration of cisplatin was 50 µM and for the MPMs it was 5 or 10 µM for MPM-2:0 and MPM-6:0, and 10 or 20 µM for MPM-3:2 and MPM-4:2, for HSC-3 and UT-SCC-24A cells, respectively. 100 μl of media ± 1% Triton X-100 served as positive and negative controls, respectively. The cells were incubated for 24 h before 20 µl of MTS reagent (CellTiter 96® Aqueous One Solution, Promega, Madison, WI, United States) was added to each well. The plate was then incubated for one more hour before absorbance was measured at 490 nm on a VersaMaxTM Microplate reader (Molecular Devices, San Jose, CA, United States). Viability was calculated using the following formula:

$$\%=\frac{Abs\;treated\;sample-Abs\;positive\;control}{Abs\;negative\;control-Abs\;positive\;control}\;x\;100$$

At least three independent experiments were performed, with triplicate wells in each experiment.

### 2.8 Confocal microscopy of lysosomes

HSC-3 and UT-SCC-24A cells were seeded at 5 × 10^4^ cells/well, in an 8-well chambered coverglass that had been pre-coated with Matrigel (#354234, Corning, Somerville, MA, United States). The following day, cells were treated with 5, 10, or 20 µM of MPM-2:0, MPM-6:0, MPM-3:2, or MPM-4:2. One well was left untreated. The cells were incubated for 24 h. 30 min before the end of the incubation period, lysotracker Deep Red (L12492, Thermo Fisher) was added to each well to a final concentration of 50 nM. The media was then removed and cells were fixed in 4% formaldehyde for 15 min at room temperature. Next, cells were washed 4 × 2 min in PBS before being incubated with DAPI (1 μg/mL in PBS) for 5 min followed by 2 × 2 min washing in PBS. Imaging was performed on the LSM 780 confocal microscope (Zeiss, Oberkochen, Germany).

### 2.9 Western blot for the detection of phosphorylated eIF2α

HSC-3 and UT-SCC-24A cells were seeded, 300,000 cells/well, in 6-well plates and left to adhere overnight. The media was then removed and replaced by 2 ml complete DMEM/F-12 containing 5, 7.5, 10 or 20 µM of MPM-2:0, MPM-6:0, MPM-3:2 or MPM-4:2, or 50 µM cisplatin. Some cells were left untreated. The cells were then incubated for 24 h. Next, the cell supernatants were collected before the cells were trypsinized and spun down with the supernatants. The cells were then washed twice in ice-cold PBS before being resuspended in 100 µl RIPA buffer (25 mM Tris-HCl (pH 7,6), 150 mM NaCl, 0.1% SDS, 0.5% sodium deoxycholate, 1% Triton X-100) containing 1% protease inhibitor (SIGMAFASTTM, #S8830, Sigma-Aldrich) and incubated on a shaker for 30 min. Next, the samples were sonicated and then kept at −70°C before being used. The samples were kept on ice or at 4°C during the whole procedure. The samples were thawed on ice and the protein concentration was measured using the DC Protein Assay kit (#5000111, Bio-Rad Laboratories, Hercules, CA, United States). 8 μg of protein was taken from each sample and mixed with DTT (final concentration 50 mM) and 20% 5 × sample buffer (250 mM Tris-HCl pH 6.8, 10% SDS, 20% Glycerol, 0.01% Bromphenol Blue). Next, the samples were boiled for 5 min and loaded on a NuPAGE® 10% Bis–Tris Gel (Thermo Fisher Scientific) before being electro-transferred to a polyvindiline dilfluoride (PVDF) immobilon-P membrane (Merck, Rahway, NJ, United States). The membrane was blocked for 1 h with 5% BSA in TBST and then incubated overnight at 4°C with the primary antibody targeting phosphorylated eIF2α (#ab32157, Abcam, Cambridge, United Kingdom) diluted 1:1,000 in TBST 5% BSA. Next, the membrane was washed three times in TBST and incubated with an HRP-conjugated goat anti-rabbit secondary antibody (#4050-05, Southern Biotech, Birmingham, AL, United States) diluted 1:2000 in TBST 5% BSA at room temperature for 1 h. The membrane was washed three times in TBST before it was incubated for 5 min with pre-mixed chemiluminescent peroxidase substrate-3 (Sigma-Aldrich) and subsequently imaged on an ImageQuant LAS 4000 (GE Healthcare, Chicago, IL, United States). For detection of non-phosphorylated eIF2α and β-actin, the membrane was first stripped by being incubated with 5 ml 1x stripping buffer (#2502, Merck) for 15 min at room temperature. The membrane was then washed three times in water. Blocking, incubation with antibodies, and detection was then performed as before but with primary antibodies targeting eIF2α (#ab242148, Abcam, diluted 1:2000) and β-actin (#A1978, Sigma-Aldrich, diluted 1:2000). The secondary antibodies were HRP-conjugated goat anti-rabbit (#4050-05, Southern Biotech) diluted 1:2000 and HRP-conjugated goat anti-mouse (#A2554, Sigma-Aldrich) diluted 1:40,000. Analysis of band intensities was performed in Image Studio Lite Ver 5.2 (https://www.licor.com/bio/image-studio-lite/).

### 2.10 Flow cytometric detection of cell surface calreticulin

This procedure was based on a published method (Liu et al., 2020), but was performed with some modifications. Briefly, HSC-3 and UT-SCC-24A cells were seeded, 300,000 cells/well, in 6-well plates and left to adhere overnight. The media was then removed and replaced by 2 ml complete DMEM/F-12 containing 5, 7.5, 10 or 20 µM of MPM-2:0, MPM-6:0, MPM-3:2 or MPM-4:2, or 50 µM cisplatin. Some cells were left untreated. The cells were then incubated for 24 h. Next, the cell supernatants were collected before the cells were trypsinized and spun down with the supernatants. The cells were then washed in PBS before being incubated with the viability dye Zombie Violet (#423114, BioLegend, San Diego, CA, United States) diluted 1:500 in PBS for 20 min. Next, the cells were incubated with an anti-calreticulin antibody (#ab2907, Abcam) diluted 1:100 in FACS buffer (2%BSA in PBS) for 30 min. The cells were then fixed in 4% formaldehyde for 15 min and washed twice in FACS buffer before being incubated with the secondary antibody (#A11034, Thermo Fisher Scientific) diluted 1:250 in FACS buffer for 30 min. The cells were washed in FACS buffer and analyzed on a LSR Fortessa (BD Biosciences) within 1 week. Analyses were performed in FlowJo™ v.10 (https://www.flowjo.com/).

### 2.11 Luminescence based detection of extracellular ATP

HSC-3 and UT-SCC-24A cells were seeded, 10,000 cells/well in 100 µl complete media in solid white 96-well plates and left to adhere overnight. The following day, 50 µl of complete media containing either cisplatin, MPM-2:0, MPM-6:0, MPM-3:2, or MPM-4:2 was added along with 50 µl of RealTime-Glo™ Extracellular ATP Assay Substrate (#GA5010, Promega). The final concentration of cisplatin was 50 μM, while the final concentration of MPM-2:0 and MPM-6:0 was either 5 or 10 μM, and the final concentration of MPM-3:2 and MPM-4:2 was 10 or 20 µM. Next, luminescence was read on a CLARIOstar microplate reader (BMG LABTECH) every 30 min for the first 3 h, followed by once every hour until the cells had been stimulated for 8 h, and finally once at 24 h of stimulation. The assay was performed with duplicate wells and three independent experiments were performed.

### 2.12 Western blot for the detection of extracellular HMGB1

Detection of HMGB1 in the supernatant of MPM treated HSC-3 and UT-SCC-24A cells was performed as previously described (von Hofsten et al., 2022). Briefly, HSC-3 and UT-SCC-24A cells were seeded, 100,000 cells/well in 12-well plates and left to adhere overnight. The following day, the media was removed and replaced with media containing either 50 µM cisplatin, 5 or 10 µM MPM-2:0 or MPM-6:0, or 10 or 20 µM MPM-3:2 or MPM-4:2. The cells were incubated for 24 h before supernatants were collected and centrifuged to remove debris. Supernatant samples were mixed with DTT (final concentration 50 mM) and sample buffer before being boiled for 5 min and loaded on a NuPAGE® 10% Bis–Tris Gel (Thermo Fisher Scientific) before being electro-transferred to a polyvindiline dilfluoride (PVDF) immobilon-P membrane (Merck, Rahway, NJ, United States). The membrane was blocked for 1 h with 5% non-fat dry milk in TBST and then incubated overnight at 4°C with the primary antibody targeting HMGB1 (#ab18256, Abcam) diluted 1:1,000 in TBST 5% non-fat dry milk. Next, the membrane was washed three times in TBST and incubated with an HRP-conjugated goat anti-rabbit secondary antibody (#4050-05, Southern Biotech, Birmingham, AL, United States) diluted 1:2000 in TBST 5% non-fat dry milk at room temperature for 1 h. The membrane was washed three times in TBST before it was incubated for 5 min with pre-mixed chemiluminescent peroxidase substrate-3 (Sigma-Aldrich) and subsequently imaged on an ImageQuant LAS 4000 (GE Healthcare, Chicago, IL, United States). Analysis of band intensities was performed in Image Studio Lite Ver 5.2 (https://www.licor.com/bio/image-studio-lite/).

### 2.13 Migration and invasion assays

Two Incucyte image lock 96-well plates (#4379, Sartorius) were coated with 50 µl myogel (0.5 mg/ml) per well. The next day, the media remaining in the wells was removed before HSC-3 cells were seeded at a density of 25,000 cells/well in 100 µl. The cells were left to adhere, and the following day, a scratch was made in the middle of each well using the Incucyte 96-pin WoundMaker tool (Sartorius). The wells were then washed once with media. For the migration assay, the media in each well was then replaced with 100 µl of media containing 0.1, 1 or 10 µM of MPM-2:0 or MPM-4:2. For the invasion assay, the media was replaced by 50 µl of a gel consisting of serum-free media, myogel (2.4 mg/ml), type I rat tail collagen (0.8 mg/ml, Corning, NY, USA) and 0.1, 1 or 10 µM of MPM-2:0 or MPM-4:2. The plate was incubated at 37°C for 1 h to let the gel solidify. Next, 100 µl of media, also containing 0.1, 1 or 10 µM of MPM-2:0 or MPM-4:2 was added on top of the gel. Both the migration and invasion plates were placed in the Incucyte S3 (Sartorius) and imaged at ×10 magnification every 2 hours for 24 h in total. The Incucyte software was used to analyze the relative wound density.

### 2.14 Statistical analysis

Statistical analyses were performed in GraphPad Prism 9.0 (https://www.graphpad.com/). A *p*-value of <0.05 was considered statistically significant. In all graphs, asterisks indicate significant differences: *p < 0.05, **p < 0.01, ***p < 0.001, ****p < 0.0001.

## 3 Results

### 3.1 Synthesis of the MPM library

**FIGURE 1:**
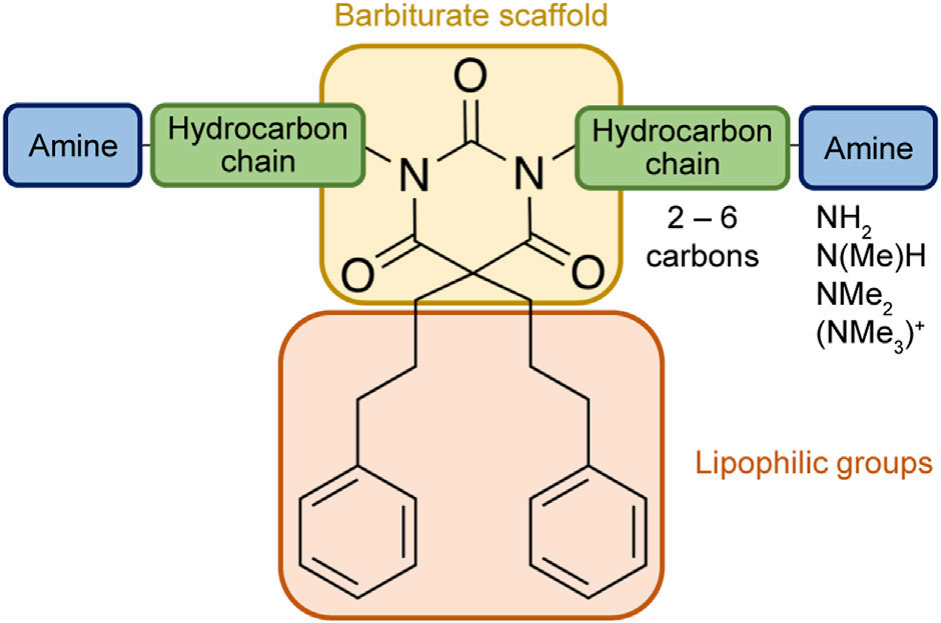
General structure of the marine product mimics (MPMs) included in this study. All compounds share the same barbiturate scaffold and lipophilic groups. All compounds also have two identical cationic groups which consist of a hydrocarbon chain of 2–6 carbons and an amine group which is either primary, secondary, tertiary, or quaternary. The cationic groups of the different compounds are demonstrated to the right.

Based on the structure of the previously reported marine natural product mimic MPM-1 (von Hofsten et al., 2022), a panel of nine structurally related amphipathic barbiturates was developed. All compounds share the same barbiturate scaffold and lipophilic groups that are present in MPM-1 (Figure 1). All new compounds also have two identical hydrocarbon chains that each contain one amine group and represent the cationic parts of the compounds. However, the length of the hydrocarbon chain and the number of methyl groups attached to the amine group varies between the different compounds. All final compounds are referred to as marine product mimics (MPM). The compound codes reflect the chemical structure and are given as follows: MPM-x:y, in which x denotes the number of carbon atoms in each hydrocarbon chain attached to the barbiturate scaffold and y is the number of methyl groups attached to the amine group. According to this system, the previously reported compound MPM-1 would be named MPM-4:0, but to avoid confusion, we will continue to refer to this compound as MPM-1.

**Scheme 1:**
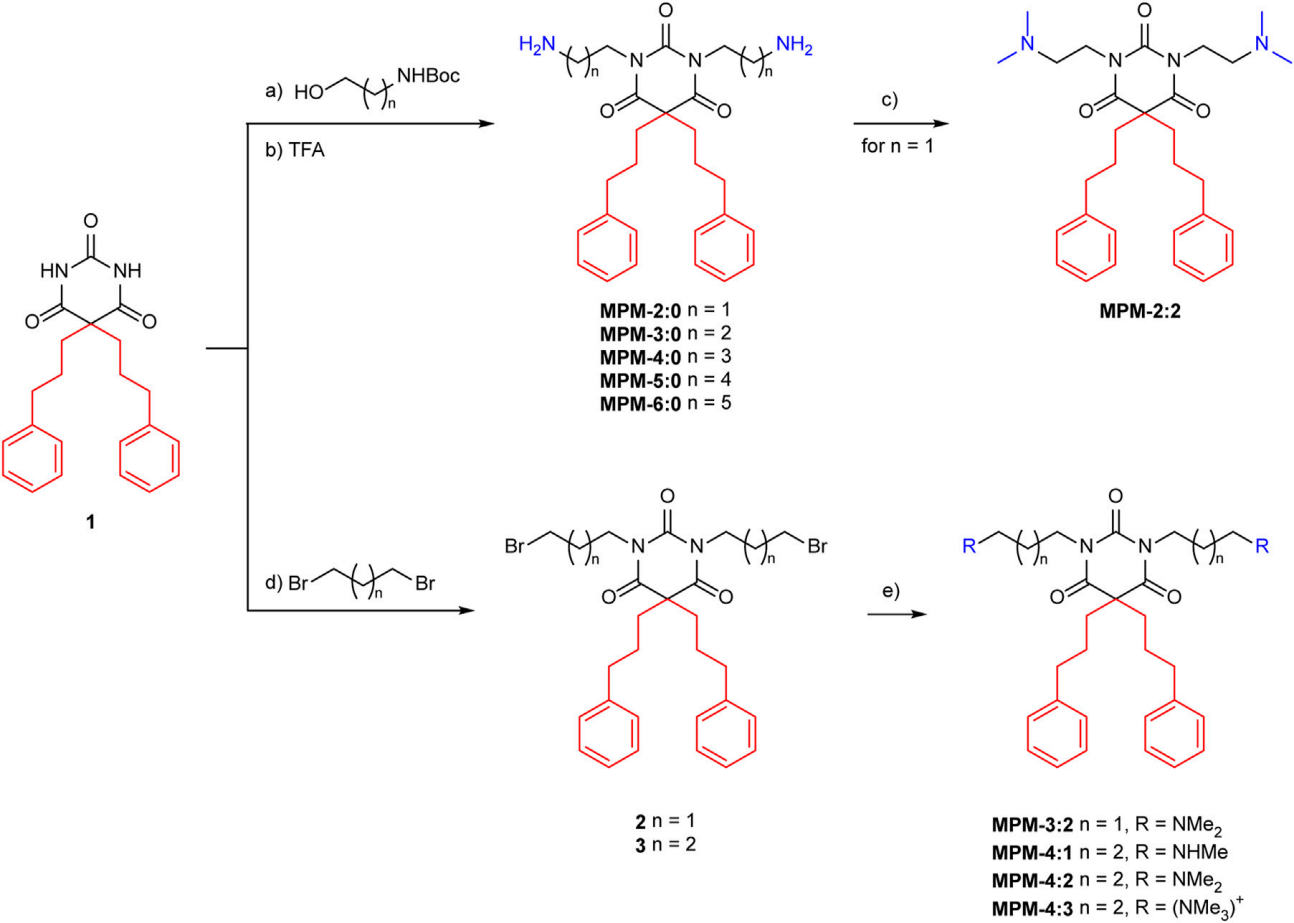
Synthesis of the MPMs. Conditions: **(A)** DIAD, PPh_3_, anhydrous DCM, 0°C to r.t.; **(B)** TFA, DCM, r.t., 51%–93% o2s; **(C)** formaldehyde (37%_(aq)_), NaBH_3_CN, AcOH, MeOH, r.t., 96%; **(D)** Cs_2_CO_3_, acetone, 60°C, 68%–86%; **(E)** NH_2_Me or NH(Me)_2_ or N (Me)_3_, anhydrous MeCN, 70°C, 63%–97%. All MPMs were obtained as di-TFA salts.

Starting from dialkylated barbituric acid 1, all MPMs were synthesized based on modified procedures previously developed in our group (Scheme 1) (Langer et al., 2022; von Hofsten et al., 2022). N,N′-Dialkylation of substituted barbituric acid 1 was easily achieved under standard Mitsunobu conditions, employing diisopropyl azodicarboxylate (DIAD), triphenylphosphine (PPh3) and an aliphatic N-Boc protected amino alcohol of choice in anhydrous dichloromethane (DCM) (Scheme 1, upper pathway). The resulting tetrasubstituted barbituric acids were treated with TFA in DCM to yield MPM-2:0, MPM-3:0, MPM-1, MPM-5:0 and MPM-6:0 as di-TFA salts (51%-93% yield over two steps). For some derivates small amount of reduced DIAD, a byproduct of the Mitsunobu reaction, was observed. The byproduct could be removed by trituration with ice-cold diethyl ether (Et2O). To synthesize the target molecules containing methylated amine groups a different approach was chosen, as free amines did not react under Mitsunobu conditions. Instead, barbituric acid 1 was treated with 1,3-dibromopropane or 1,4-dibromobutane under basic conditions to deliver tetrasubstituted barbiturates 2 and 3 (68% and 86% yield), respectively (Scheme 1, lower pathway). Combining barbiturates 2 and 3 with organic solutions of methylamine, dimethylamine and trimethylamine in anhydrous acetonitrile (MeCN) at elevated temperatures delivered MPM-4:1, MPM-4:2, MPM-4:3 and MPM-3:2 as di-TFA salts after purification by reversed phase chromatography (63%-97% yield). We investigated the same sequence towards MPM-2:2, namely, to alkylate 1 with 1,2-dibromoethane, but no conversion was observed. We therefore chose to di-methylate the primary amines of MPM-2:0 to obtain MPM-2:2. Treatment of MPM-2:0 with an aqueous formaldehyde solution in the presence of sodium cyanoborohydride (NaBH3CN) and acetic acid in methanol (Eschweiler-Clarke conditions) cleanly delivered the di-TFA salt of MPM-2:2 after reversed phase chromatography (96% yield). A detailed description of the experimental data, NMR data and purity analysis can be found in the Supplementary Data.S1

### 3.2 Biological activity of the MPMs

An overview of the novel MPMs is presented in Table 1. As a measure of the lipophilicity of the compounds, CLogP values were calculated for the compounds. The overall high values demonstrate that all the compounds, except MPM-4:3, are relatively lipophilic. Simultaneously, high pKa values imply that at physiological pH, most of the compounds will have positively charged amine groups. Thus, the MPMs can be described as amphipathic molecules due to lipophilic substituents and hydrophilic side chains. MPM-4:3 contains quaternary amine groups, which remain positively charged at all pH levels, which is why MPM-4:3 has no pKa value and a negative CLogP.

**TABLE 1:** Overview of the MPMs and their hemolytic activity against human red blood cells (expressed by the EC_50_ values in µM), as well as their CLogP and pKa values.

Core structure	Comp. ID^a^	R	EC_50_ ^b^	CLogP^c^	pKa^d^
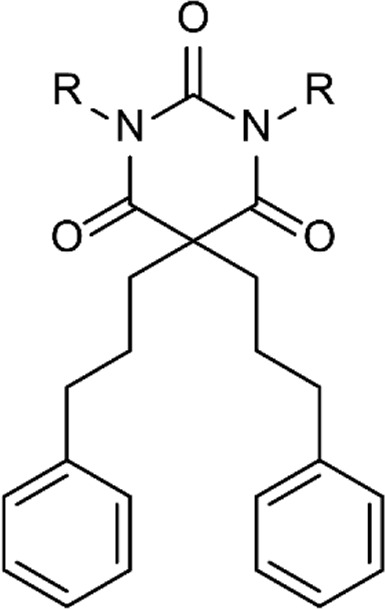	MPM-1	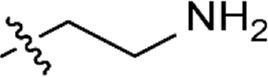	>500^e^	4.45	9.81
	MPM-2:0	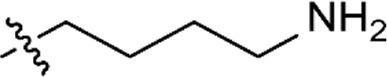	379	2.63	8.10
	MPM-3:0	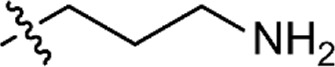	>500	3.54	9.22
	MPM-5:0	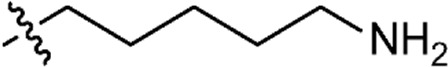	313	5.36	9.96
	MPM-6:0	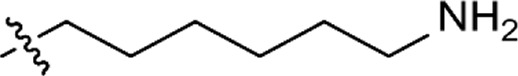	92	6.27	10.24
	MPM-4:1	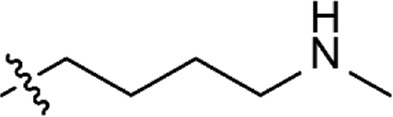	>500	5.15	9.73
	MPM-4:2	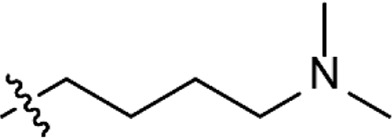	>500	5.69	9.68
	MPM-4:3	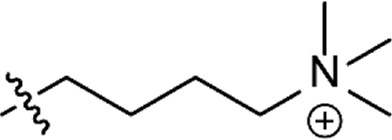	>500	−0.19	–
	MPM-2:2	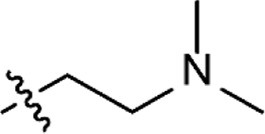	>500	3.60	9.09
	MPM-3:2	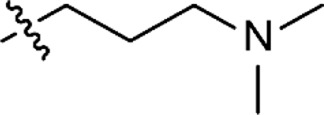	>500	4.78	8.06

^a^ Compound codes are constructed as follows: MPM-x:z, where x denotes the number of carbon atoms in the hydrocarbon chains and y is the number of methyl groups on the amine group.

^b^ Values given as greater than correspond to the highest concentration (500 μM) tested in the hemolysis assay.

^c^ CLogP values were calculated for the neutral molecules, except for MPM-4:3 (calculated with DataWarrior v5.5.0).

^d^ Values calculated with ChemBioDraw Ultra v21.0.0.28. –: not calculated.

^e^ Value from von Hofsten et al., 2022.

As a preliminary measure of the toxicity of the MPMs, their hemolytic activity was assessed and is expressed as EC_50_ values. Except for MPM-6:0, none of the MPMs showed hemolytic activity against red blood cells. MPM-6:0 had a relatively high EC_50_ of 92 μM. The MPMs were also assessed for antimicrobial activity, which demonstrated that they generally had low activity against bacteria (Supplementary Table S2). However, MPM-6:0 again demonstrated the highest activity against both gram-positive and gram-negative bacteria. Taken together, these results suggest that the MPMs do not have the ability to disrupt biological membranes. However, additional experiments are required to attest this hypothesis.

**FIGURE 2:**
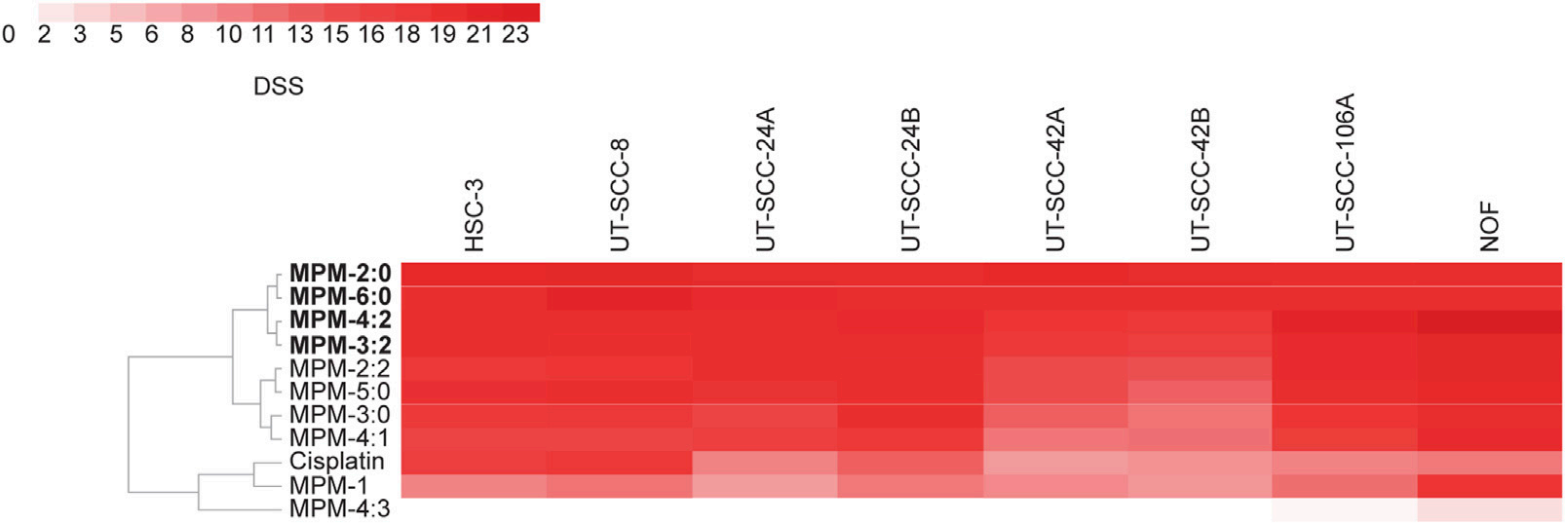
The potency of the MPMs was determined against a panel of seven head and neck squamous cell carcinoma cell lines as well as non-malignant normal oral fibroblasts (NOF) in a high throughput drug screening. The drug sensitivity scores (DSS) for each cell line and compound was calculated and presented as a heat map. The higher the DSS, the more potent the drug is. Exact DSS values are presented in Supplementary Table S3.

To study the anti-cancer effects of the MPMs, we performed a high-throughput drug screening. All compounds were screened against a panel of seven HNSCC cell lines and one normal oral fibroblast cell line (NOF), representing healthy cells. The cancer cell lines were selected to represent HNSCC tumors from different locations, as well as both primary and metastatic sites. Clinical and pathological characteristics of the cell lines are summarized in Supplementary Table S1. The UT-SCC cancer cell lines were originally established in the laboratory of Prof. Grénman and have been used in several drug screening studies previously (Lepikhova et al., 2018; Tuomainen et al., 2021). HSC-3 is a commercial cell line which has previously been used to study MPM-1 (von Hofsten et al., 2022). For comparative reasons, the screening included cisplatin in addition to the MPMs. The results from the drug screening were used to calculate drug sensitivity scores (DSS) and are presented as a heat map in Figure 2. Exact DSS can be found in Supplementary Table S3. The DSS is calculated from the dose-response curves for the different compounds and incorporates the slope of the curve, the IC50, and the minimum and maximum responses into a single metric (Yadav et al., 2014). Inactive compounds have a DSS of 0, while the more potent a compound is, the higher the score is (darker color in the heat map).

All compounds except MPM-4:3 effectively reduced the viability of all cell lines, as seen from their high DSS. The compounds were more potent than cisplatin for all cell lines except HSC-3 and UT-SCC-8, for which the potency of cisplatin and the MPMs was similar. Some MPMs were noticeably less potent against the cell lines UT-SCC-42A and UT-SCC-42B than the other cell lines. Overall, the four compounds MPM-2:0, MPM-6:0, MPM-3:2 and MPM-4:2 had the highest DSS and were therefore found to be the most potent. They showed a similar degree of activity against all cell lines, including UT-SCC-42A and UT-SCC-42B.

No compound selectively targeted the cancer cell lines over the NOFs. Surprisingly, there was a slight tendency towards selectivity for the NOFs, which was especially prominent for MPM-1. However, this effect can possibly be explained by the fact that the cancer cell lines and NOFs were cultured in different cell culture medias, which use different buffer systems. To test this hypothesis, MTS viability assays were performed with HSC-3 and UT-SCC-24A cells treated with the different MPMs diluted in three different media. The results revealed that the compounds were generally the least potent in DMEM/F-12, which contains HEPES buffer and should maintain a stable pH of 7.4 (Supplementary Figure S1). The compounds were the most potent in DMEM, which utilizes a sodium bicarbonate buffer system which is sensitive to changes in CO_2_ concentration in the surrounding environment, causing the pH to fluctuate. DMEM/F-12 which had been adjusted to a pH of 8 increased the potency of the compounds. According to the relatively high pKa values of the MPMs, increasing the pH above 7.4 would cause a larger proportion of compounds to be unprotonated. This result thus suggests that protonation of the MPMs affects their potency. Despite the fact that the MPMs were the most potent in DMEM, it was decided that all other experiments should be performed in DMEM/F-12 because the lower pH of this media is likely more representative of the microenvironment in a real tumor (Apostolova and Pearce, 2022).

As some chemotherapeutic drugs are known to have synergistic effects with irradiation and since irradiation is part of the standard treatment regimen for HNSCC patients, we also studied the cytotoxic effect of the MPMs when given in combination with irradiation. However, there were no synergistic effects of combining the MPMs with irradiation (Supplementary Table S4).

### 3.3 Mode of death induced by MPMs

For studies on the mechanism of action and the mode of death induced by the MPMs, the four most potent compounds (MPM-2:0, MPM-6:0, MPM-3:2 and MPM-4:2) were considered the most interesting and were therefore selected for these studies. The 2 cell lines HSC-3 and UT-SCC-24A were also chosen for this objective. HSC-3 was selected because it has previously been used to study the original compound MPM-1 (von Hofsten et al., 2022), and UT-SCC-24A was included to study whether the MPMs would affect different cell lines differently. As could be seen from the screening, MPM-2:0 and MPM-6:0 were somewhat more potent than MPM-3:2 and MPM-4:2. However, due to the design of the high throughput screening, which utilized a 10-fold dilution series of the compounds, it could not differentiate the potency of the different MPMs on the different cell lines at a detailed level. In general, the UT-SCC-24A cell line needed to be treated with higher concentrations of the MPMs as compared to HSC-3, to achieve a similar degree of cell death. In the mechanistic studies, the cells were treated with concentrations which were selected to render the cells heavily affected by the treatment, but not yet completely dead. The same assessment was made regarding cisplatin, which has previously been shown to effectively reduce the viability of both HSC-3 and UT-SCC-24A cells at concentrations ranging between 10 and 100 µM (Mandic et al., 2005; Ahmed et al., 2009). The current compound screening also demonstrated that cisplatin was generally somewhat less potent than the MPMs and should therefore be used at higher concentrations.

**FIGURE 3:**
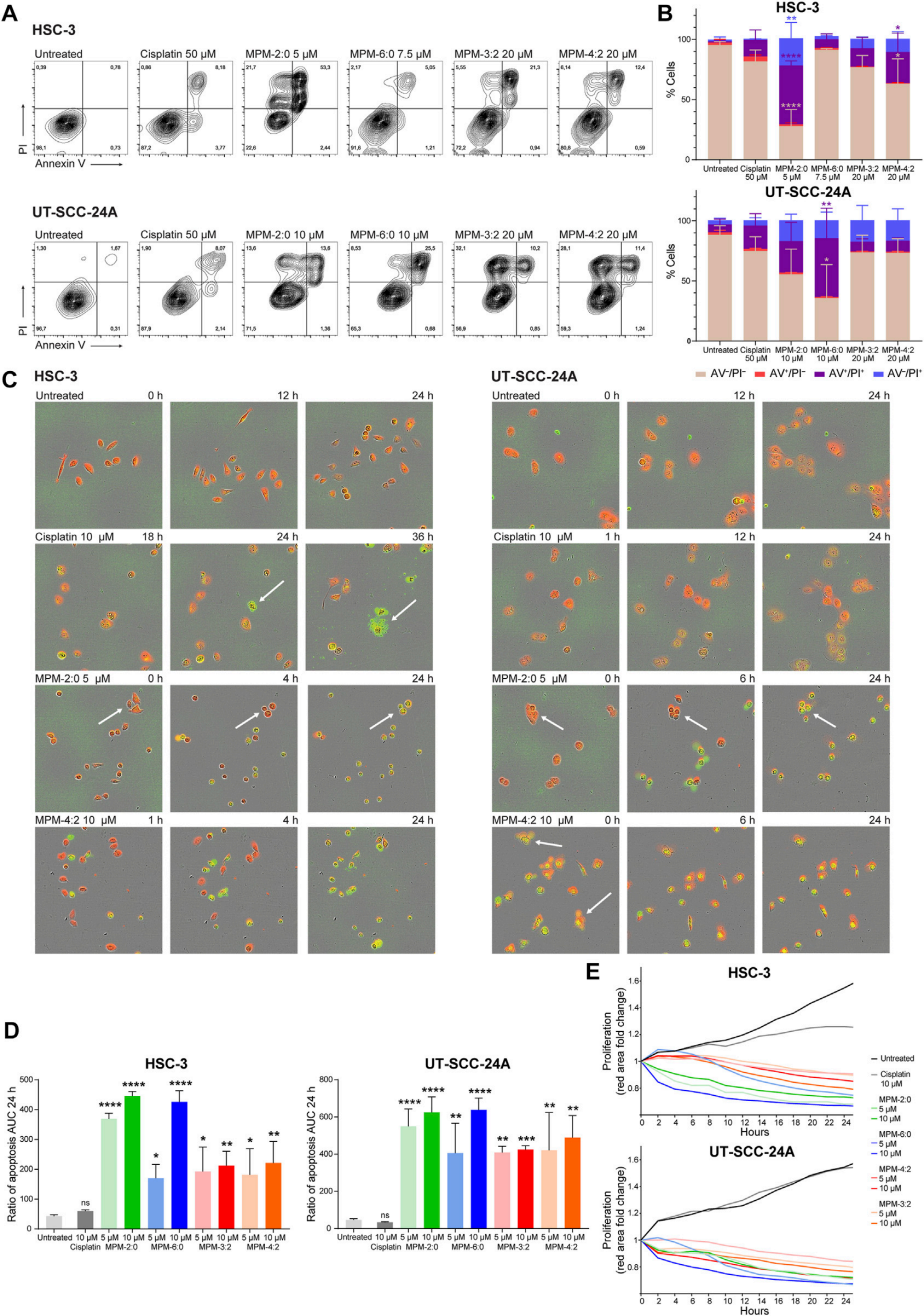
The cell surface expression of phosphatidylserine (determined by staining with Annexin V), which characterizes cells in early apoptosis, was analyzed by flow cytometry in HSC-3 and UT-SCC-24A cells treated with MPMs **(A)**. The percentage of Annexin V–/PI–, Annexin V+/PI–, Annexin V–/PI+ and Annexin V+/PI + cells was determined and the mean from three independent experiments is shown in **(B)**. Statistically significant differences, as compared to the untreated cells were determined by one-way ANOVA and Dunnett’s *post hoc*. A live cell apoptosis assay was performed where HSC-3 and UT-SCC-24A cells were stained red, treated with MPMs, and a dye which becomes fluorescent (green) upon cleavage by caspase 3/7 was added. Representative images are shown in **(C)**, with arrows pointing to cells referenced in the text. Three independent experiments were performed, and the mean extent of apoptosis was quantified by calculating the area under the curve (AUC) for the ratio of apoptosis (number of green + red objects) for 24 h **(D)**. The statistical differences were determined by one-way ANOVA and Dunnett’s *post hoc*. The degree of proliferation was determined by quantifying the area of red fluorescence and is expressed as fold change relative to timepoint 0 **(E)**.

To begin studying the mode of death induced by the MPMs, flow cytometric assessment of the externalization of phosphatidylserine (PS), which is a hallmark for cells in early apoptosis, was performed (Figures 3A,B). HSC-3 and UT-SCC-24A cells were treated with MPM-2:0, MPM-6:0, MPM-3:2, MPM-4:2 or cisplatin and then stained with FITC-labeled Annexin V, which binds to PS. Propidium iodide (PI), which only penetrates and stains cells with a compromised cell membrane, was added for the detection of cells which had lost their plasma membrane integrity. Cisplatin, which is known to trigger apoptosis, caused the appearance of a small, but clearly apoptotic (Annexin V+/PI−) population in both HSC-3 and UT-SCC-24A cells. A similar population was not seen in any of the MPM treated cells. In some HSC-3 samples, a population of Annexin V+ events with low fluorescence intensity for PI was seen. These could represent apoptotic cells. However, the PI fluorescence intensity was noticeably higher in these populations compared to untreated cells or the apoptotic population in cisplatin treated cells, suggesting that there had been some degree of membrane rupture in this population. Moreover, the MPMs also caused the appearance of an Annexin V−/PI + population, which are cells that have lost their membrane integrity but have not externalized PS. These cells were probably not apoptotic.

However, we also performed a live cell imaging apoptosis assay which monitors the activation of caspase 3 and 7. HSC-3 and UT-SCC-24A cells were stained with a red fluorescent marker and treated with MPM-2:0, MPM-6:0, MPM-3:2 or MPM-4:2 in two different concentrations (5 μM and 10 µM). A dye which becomes fluorescent (green) only upon cleavage by caspase 3/7 was also added and the cells were monitored for proliferation and caspase activation for 24 h (Figures 3C,D).

All MPMs caused rapid cell death in both cell lines, as seen by the complete halt of proliferation and movement (Figure 3E). Cisplatin also inhibited proliferation in HSC-3 cells, but not in UT-SCC-24A cells. The MPMs also quickly caused activation of caspase 3/7, which indicates the activation of apoptosis. Cisplatin only induced apoptosis in a minority of HSC-3 cells and in no UT-SCC-24A cells. However, the cisplatin treated cells that did die displayed a morphology which is typical for apoptosis. The formation of apoptotic bodies was clearly visible in these cells. The morphological changes that occurred to the MPM treated cells were generally not reminiscent of apoptosis. Both the HSC-3 and UT-SCC-24A cells treated with MPM-2:0 simply became round. In some cells, such as the UT-SCC-24A cells treated with MPM-4:2, the formation of some bleb-like structures, which could be reminiscent of apoptotic bodies, was seen.

**FIGURE 4:**
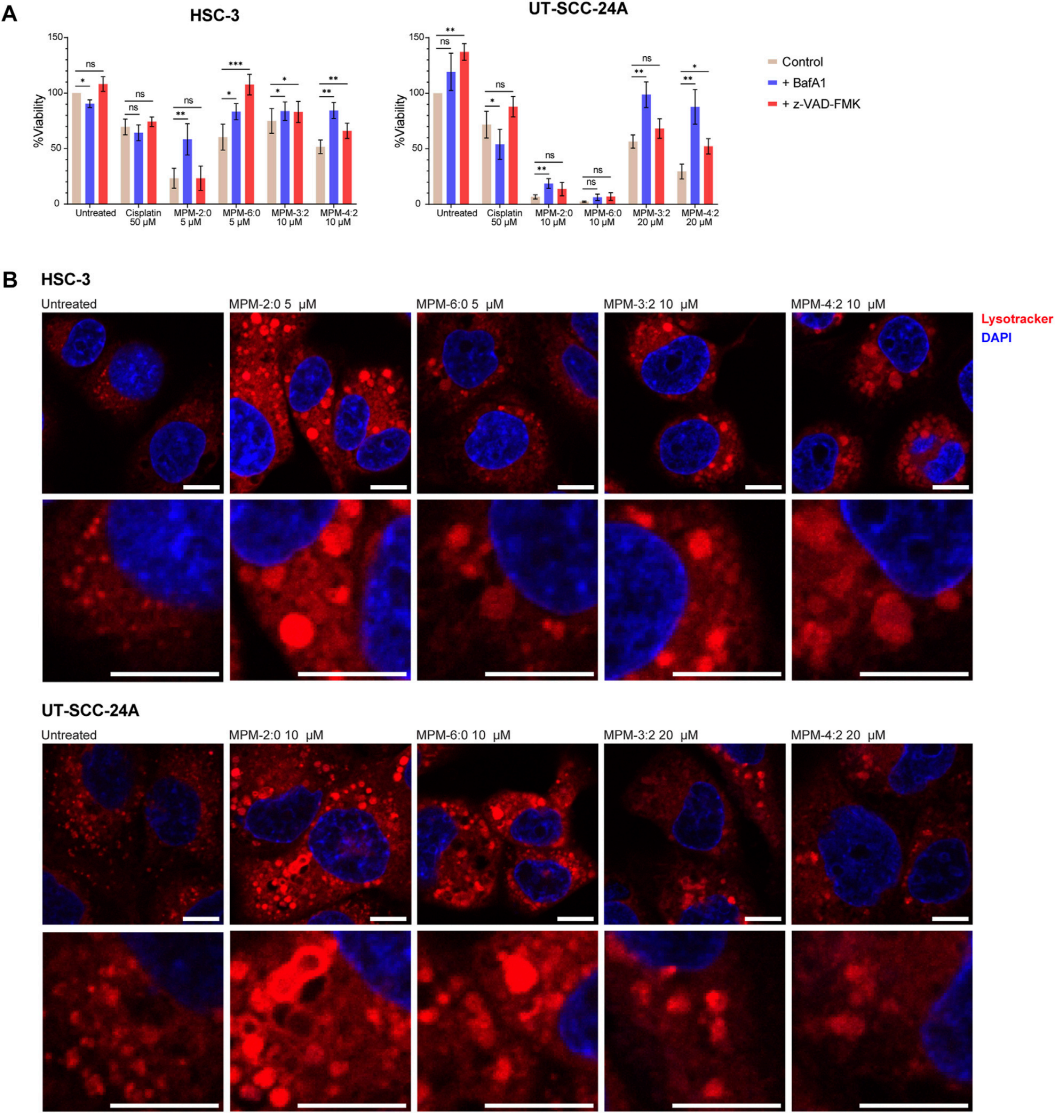
The viability of HSC-3 and UT-SCC-24A cells treated with MPMs in the presence of bafilomycin A1 (BafA1) or the pan caspase inhibitor Z-VAD-FMK was determined by means of the MTS assay **(A)**. The graph shows the mean from three independent experiments. Significant differences were determined by paired t-tests. **(B)** HSC-3 and UT-SCC-24A cells were left untreated or were treated with the MPMs for 24 h before being stained with Lysotracker Deep Red and imaged by confocal microscopy. All scale bars are 10 µm.

To further study the mode of death induced by the MPMs, MTS viability assays were performed on MPM treated HSC-3 and UT-SCC-24A cells in the presence of the pan-caspase inhibitor z-VAD-FMK or the V-ATPase inhibitor Bafilomycin A1 (BafA1) (Figure 4A). The function of the V-ATPase is to pump protons into acidic cellular compartments like the lysosomes, to keep their internal pH low. By inhibiting the function of the V-ATPase, BafA1 therefore increases the pH inside such compartments. Since the activity of caspase 3/7 had been observed, we hypothesized that the inhibition of caspase activity might rescue cells from cell death induced by the MPMs. Although not statistically significant, z-VAD-FMK seemed to have some protective effects against cisplatin for both cell lines, while BafA1 had the opposite effect. For the MPMs, the results demonstrated that z-VAD-FMK did provide some degree of protection in both cell lines, suggesting that apoptosis may represent some of the MPM induced cell death. However, BafA1 generally increased the viability of MPM treated cells more efficiently than z-VAD-FMK. Only for HSC-3 cells treated with MPM-6:0 did z-VAD-FMK increase viability more than BafA1. Protection by BafA1 indicates that the MPMs may be lysosomotropic compounds (Nadanaciva et al., 2011).

To further study whether the MPMs could be lysosomotropic, the morphology of the lysosomes in HSC-3 and UT-SCC-24A cells treated with MPM-2:0, MPM-6:0, MPM-3:2, and MPM-4:2 was studied by staining the cells with the fluorescent dye lysotracker and imaging the cells using confocal microscopy. Untreated HSC-3 and UT-SCC-24A cells generally contained many small lysosomes (Figure 4B). However, treatment with any of the MPMs drastically changed the lysosomal morphology in both cell lines. Many lysosomes became considerably larger, an effect which is associated with lysosomotropism (Seo et al., 2014).

### 3.4 Immunogenic potential of the MPMs

To examine the ability of the MPMs to induce immunogenic cell death, we studied their ability to activate the integrated stress response, which is highly associated with immunogenic cell death (Bezu et al., 2018). The integrated stress response is characterized by the phosphorylation of the eukaryotic initiation factor 2α (eIF2α). Western blot analysis of MPM treated HSC-3 and UT-SCC-24A cells demonstrated that some of the MPMs could significantly induce some phosphorylation of eIF2α (Figures 4A,B), indicating that they can activate the integrated stress response.

**FIGURE 5:**
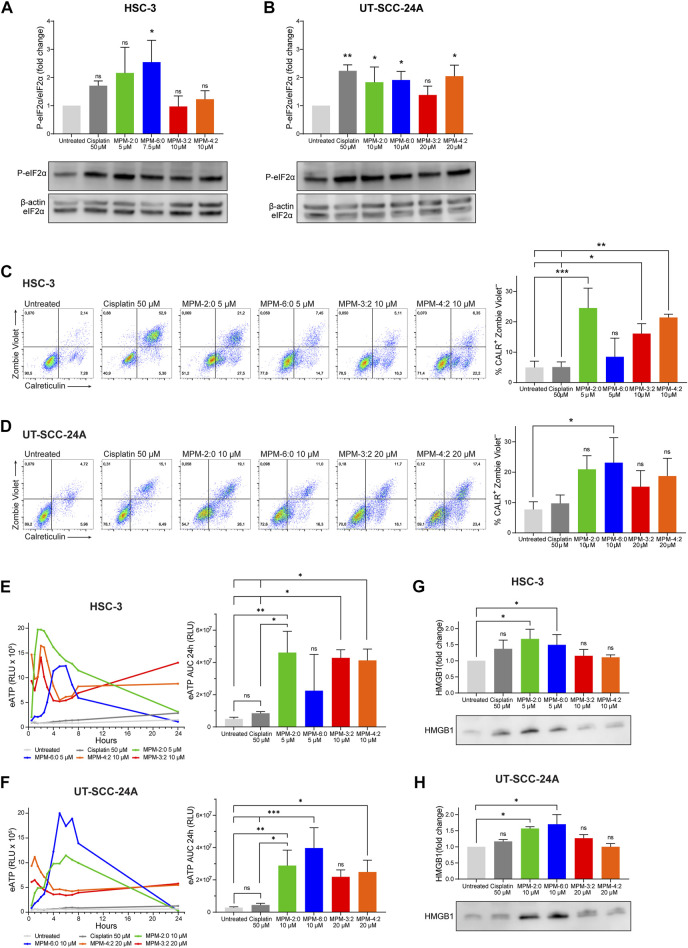
The phosphorylation of eIF2α in HSC-3 **(A)** and UT-SCC-24A **(B)** cells treated with MPMs was analyzed by Western blot. Band intensities were quantified and normalized to non-phosphorylated eIF2α. The cell surface expression of calreticulin was analyzed by flow cytometry in HSC-3 **(C)** and UT-SCC-24A **(D)** cells treated with MPMs. The percentage of live cells expressing calreticulin (Zombie Violet–/CALR+) was determined and the graph shows the mean from three independent experiments. Statistical differences were determined by one-way ANOVA and Bonferroni’s *post hoc*. Extracellular ATP (eATP) was measured in the supernatant of MPM treated HSC-3 **(E)** and UT-SCC-24A **(F)** cells by means of a luminescence-based assay at several time points over the course of 24 h. The amount of eATP is proportional to the relative luminescence units (RLU). Three independent experiments were performed. The area under the curve (AUC) was calculated and statistical analysis was performed by one-way ANOVA and Bonferroni’s *post hoc*. HMGB1 in the supernatant of untreated and MPM treated HSC-3 **(G)** and UT-SCC-24A **(H)** was analyzed by Western blot. Band intensities were quantified and normalized to the untreated sample. Statistical analysis was performed by Kruskal–Wallis test and Dunn’s *post hoc*.

The expression of calreticulin in MPM treated cells was analyzed by flow cytometry (Figures 5C,D). Since cell surface expression of calreticulin is known to occur early during immunogenic cell death, before plasma membrane integrity is lost, standard practice is to only analyze calreticulin in cells with intact plasma membranes (Liu et al., 2020). Another reason is that cells which have lost their plasma membrane integrity will also stain positively for intracellular calreticulin, making it impossible to distinguish between cell surface bound and intracellular calreticulin. Cisplatin, which is known to not cause immunogenic cell death, did not induce any cell surface expression of calreticulin. In contrast, all the MPMs could induce cell surface calreticulin. The increase in cell surface calreticulin was statistically significant for HSC-3 cells treated with MPM-2:0, MPM-3:2 and MPM-4:2. For UT-SCC-24A cells, only MPM-6:0 could induce statistically significant levels of cell surface calreticulin. Release of ATP from MPM treated HSC-3 and UT-SCC-24A cells was analyzed by means of a luminescence-based assay, which measured the relative amount of extracellular ATP (eATP) in the supernatant of treated cells at different time points up to 24 h. The results demonstrated that all MPMs could induce release of eATP (Figures 5E,F). Release of HMGB1 from MPM treated HSC-3 and UT-SCC-24A cells was analyzed by Western blot (Figures 5G,H). While all MPMs seemed to cause some release of HMGB1, the results were only statistically significant for MPM-2:0 and MPM-6:0 in both HSC-3 and UT-SCC-24A cells.

### 3.5 Effects on migration and invasion of HSC-3 cells

**FIGURE 6:**
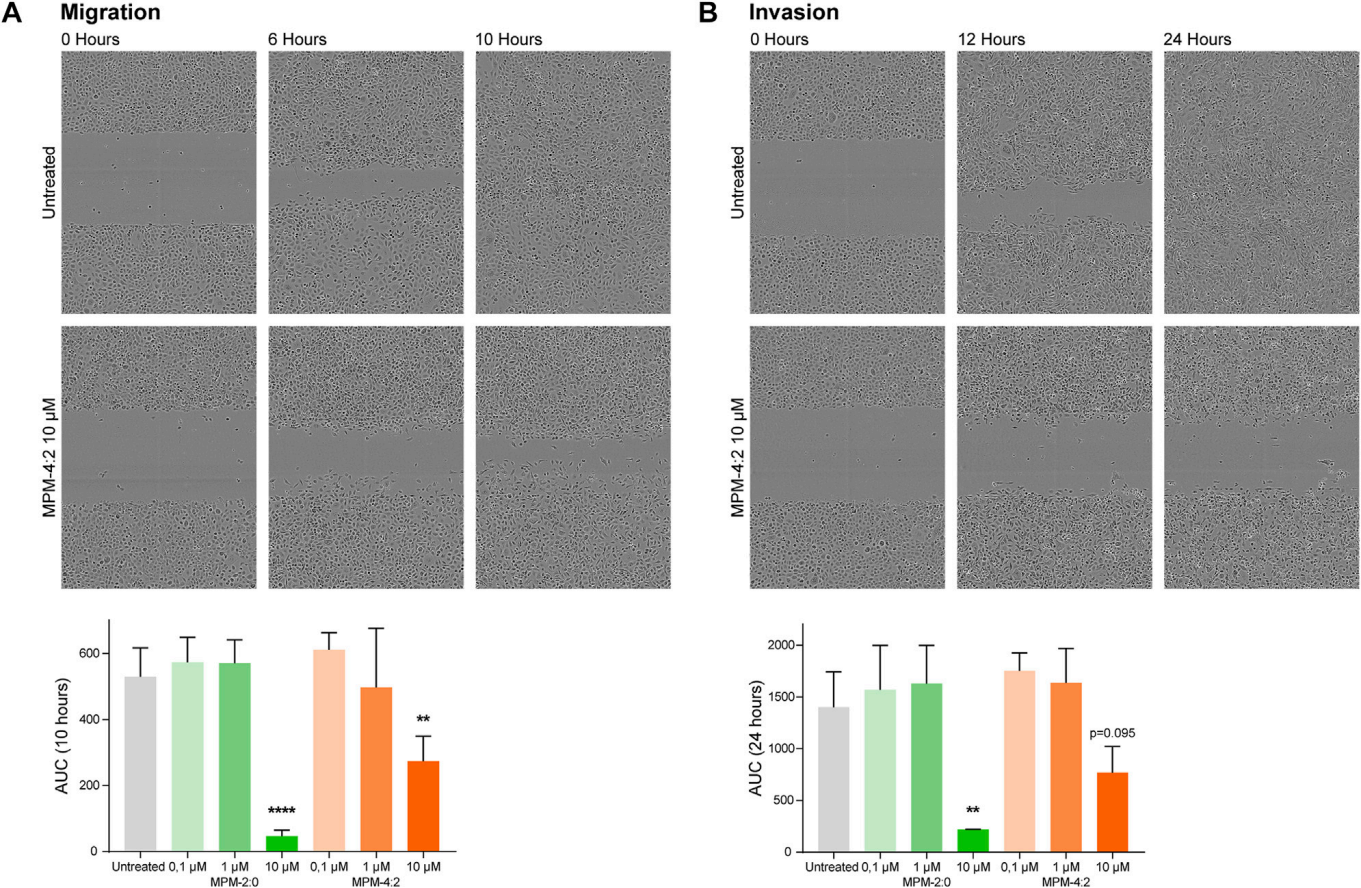
The migration and invasion of MPM treated HSC-3 cells was analyzed through scratch wound assays. The cells were left untreated or were treated with 0.1, 1 or 10 μM of MPM-2:0 or MPM-4:2. In the migration assay **(A)**, the wells were only filled with media, and in the invasion assay **(B)**, the scratch was filled with a myogel-collagen mix before the addition of media. The cells were imaged every 2 hours for 24 h. The results were quantified by calculating the area under the curve (AUC) for the relative wound density and statistical differences were calculated by one-way ANOVA and Dunnet’s *post hoc*. Four independent migration assays and three independent invasion assays were performed.

The HSC-3 cell line is very aggressive and able to migrate and invade through matrices effectively. Therefore, we wanted to study whether the MPMs could inhibit this ability and at the same time uncover any unwanted side effects relating to treatment-induced increase of migration or invasion of MPM treated cells. A standard scratch wound migration assay where cells were treated with MPM-2:0 or MPM-4:2 in a ten-fold dilution series was performed. The results showed that HSC-3 cells treated with 10 µM MPM-4:2 required more time to close the wound as compared to the untreated control cells in the migration assay (Figure 6A). The morphology of the treated cells suggested that they were heavily affected by the treatment and in the process of dying. However, after 24 h the cells were still moving, indicating that they were not completely dead (Supplementary Video S1). The same trend was observed in an invasion assay, where the scratch wound was filled with a myogel-collagen mix which the cells had to invade through. In the invasion assay, the control cells needed approximately 24 h to close the wound, whereas the cells treated with 10 µM MPM-4:2 never managed to close the wound (Figure 6B). MPM-2:0 was also included in the migration and invasion assays but at 10 µM it induced cell death so quickly that it was not possible to study the effect on migration or invasion. There was a trend towards faster wound closing for cells treated with the lower concentrations of both MPM-2:0 and MPM-4:2. However, the effect was not statistically significant.

## 4 Discussion

The current study presents a panel of novel compounds designed for use in cancer treatment *via* intratumoral injection. The structure of these compounds was based on the previously described compound MPM-1, which is a synthetic mimic of a group of natural products called *eusynstyelamides* that have been isolated from marine organisms (Strøm et al., 2018; Tadesse et al., 2011; von Hofsten et al., 2022). While MPM-1 was shown to be a potent anti-cancer compound, the current study demonstrated that, except for MPM-4:3, the novel MPMs were all more potent than MPM-1 against HNSCC cell lines. They were shown to effectively kill a range of different HNSCC cells. Studies on the mechanism of action revealed that overall, the different MPMs had similar effects on HSC-3 and UT-SCC-24A cells. However, despite the small differences in structure, there were some differences in how the compounds affected the cells.

Out of the four most potent compounds, which were selected for inclusion in the mechanistic studies, MPM-3:2 and MPM-4:2 have the most similar structure. They are both tertiary amines and there is only one carbon atom in the hydrocarbon chains that separates them. Unsurprisingly, these two compounds had very similar effects on the cells. For example, they both induced a large population of Annexin V−/PI + UT-SCC-24A cells, as seen by the flow cytometric analysis of PS externalization. Their Annexin V/PI plots for HSC-3 cells were also similar. In addition, the results from the live cell apoptosis assay, the viability assays with BafA1 and z-VAD-FMK, and the analysis of cell surface expression of calreticulin were all very similar for MPM-3:2 and MPM-4:2. This suggests that these two compounds function the same way. However, only by changing the structure slightly more, the properties of the compounds were affected. The structure of MPM-2:0 and MPM-6:0 differs by four carbons, and the results obtained for these compounds were more diverse, both when compared with each other and with MPM-3:2 and MPM-4:2. For example, MPM-6:0 stood out from the other compounds by the fact that it had a noticeably lower EC_50_ against red blood cells and that HSC-3 cells were more protected from it by z-VAD-FMK than by BafA1.

Changing the structure of the compounds also affected their potency against the HNSCC cells, although it was not evident exactly how or why that occurred. For example, the two most potent compounds, MPM-2:0 and MPM-6:0, contained the shortest and the longest hydrocarbon chains out of all the compounds, respectively. Thus, it is difficult to draw conclusions about how the hydrocarbon chain length affects potency. Moreover, MPM-3:2 and MPM-4:2, both have medium length hydrocarbon chains. On a similar note, methylation of the amine groups also seemed to affect potency, although it is unclear how. MPM-2:0 and MPM-6:0 are both primary amines, suggesting that increases potency. On the other hand, the other primary amines in this study, including the original MPM-1, were noticeably less potent than all the tertiary amines, thus indicating that may not be the case. However, we have previously seen that methylation of amine groups decreases both hemolytic and antibacterial activity (Langer et al., 2022). This trend was also seen in the current study, as none of the MPMs containing methylated amine groups had red blood cell (RBC) EC_50_ lower than 500 μM, and they generally had poor effects on bacteria. Contrary to this, both MPM-2:0 and MPM-6:0 were considerably more potent against bacteria and, as previously mentioned, MPM-6:0 was the most hemolytic compound.

Even though there were some clear differences regarding both potency and properties of the different MPMs, overall, their effect on cells was quite similar. Their potency was within the same range and they all induced a type of cell death which is reminiscent of what has previously been demonstrated for MPM-1 (von Hofsten et al., 2022). Originally, the MPMs presented in this study were created as part of a larger library of amphipathic barbiturates. Several of these were found to have antimicrobial activity and their mechanism of action in bacteria has been partly elucidated (Langer et al., 2022). It was found that these compounds could compromise the integrity of the bacterial cell membrane, which caused the bacteria to die. This effect was likely due to the amphipathic barbiturates functioning as detergents which disrupt biological membranes. Interestingly, despite effectively killing the cancer and normal cells, the MPMs generally did not affect bacteria or red blood cells. This indicates that their primary mode of action is not to disrupt the cell membrane. The inactivity of MPM-4:3, which should remain permanently protonated and therefore also permanently amphipathic, also strongly supports this conclusion. Moreover, it makes the MPMs more attractive as potential cancer drugs as they do not represent a threat to the normal flora. However, as discussed, MPM-6:0 did have considerable antimicrobial effects in addition to being the compound with the highest activity against red blood cells. This could indicate that MPM-6:0, unlike the other MPMs, may have some detergent like properties. This could be an effect of MPM-6:0 having the longest hydrocarbon chains, which makes it the most lipophilic and potentially increases its interaction with the lipid bilayer of the cell membrane. Likewise, MPM-5:0 also had relatively high activity against bacteria and the second lowest RBC EC_50_.

The fact that the MPMs generally did not affect bacteria or red blood cells suggests that their main target is located intracellularly and that they need to cross through the cell membrane to exert their action. The increased potency of the MPMs which was achieved by increasing the pH of the cell media, also supports this conclusion. When the pH is higher, an increased number of molecules are unprotonated, which makes them more lipophilic and likely more able to cross through a cell membrane, resulting in more cell death. The fact that MPM-4:3, which remains protonated at all pH levels, was completely inactive, is also in line with this theory.

The previous study on MPM-1 suggested that it is a lysosomotropic compound, which may partly induce cell death by accumulating in lysosomes and causing their dysfunction (von Hofsten et al., 2022). Lysosomotropic compounds accumulate in lysosomes because their low internal pH causes such compounds to become protonated and thereby unable to leave the lysosome (Marceau et al., 2012). When the concentration of compound within the lysosomes reaches above a certain threshold, they can act as detergents on the lysosomal membrane, causing its destabilization and the leakage of lysosomal content into the cytosol (Boya and Kroemer, 2008). Accumulation of lysosomotropic compounds in lysosomes can also cause influx of water and swelling of the lysosomes, giving the cells a vacuolated appearance (De Duve et al., 1974; Marceau et al., 2012). Compounds which contain both lipophilic and basic parts tend to be lysosomotropic. A study which looked at characteristics of lysosomotropic compounds found that a ClogP>2 and pKa between 6.5 and 11 were common traits for lysosomotropic compounds (Nadanaciva et al., 2011). This description fits all the MPMs except MPM-4:3. Further results also support the hypothesis that the MPMs are lysosomotropic. For instance, red blood cells do not contain lysosomes and are therefore not affected by lysosomotropic compounds. Accordingly, the MPMs generally had very little effect on red blood cells. In addition, staining of MPM treated HSC-3 and UT-SCC-24A cells with Lysotracker Deep Red demonstrated that the MPMs caused swelling of lysosomes. Furthermore, the fact that the potency of the MPMs was decreased in cells that were co-treated with BafA1 also supports the notion that the MPMs are lysosomotropic. BafA1 inhibits the vacuolar ATPase (V-ATPase), which is present in the lysosomal cell membrane and functions to pump protons into the lysosome to keep the internal pH low. By inhibiting the V-ATPase, BafA1 raises the lysosomal pH, which in turn causes less accumulation of lysosomotropic compounds and consequently less cell death. This phenomenon has been demonstrated for several different lysosomotropic compounds (Marceau et al., 2012).

The fact that the viability of MPM treated cells was significantly increased when they were treated with BafA1 suggests that lysosomotropism is central to their mechanism of inducing cell death. This result is in contrast to what was found for MPM-1, for which the potency was increased in cells treated with BafA1 (von Hofsten et al., 2022). From that result it was presumed that the main reason for MPM-1 induced cell death was not lysosomotropism. Instead, it was believed that MPM-1 may have a different target which it is able to reach in greater concentration when it is not trapped in lysosomes. This is a phenomenon which has been described for some lysosomotropic compounds including doxorubicin (Altan et al., 1998). This curious difference between the novel MPMs and the original MPM-1 highlights the fact that small changes in the structure of these compounds can greatly affect their mechanism of action. As such, *in vitro* mechanism studies are important to perform in order to be able to pick the best lead compounds for further optimization for cancer treatment.

Lysosomotropic compounds have been shown to be able to trigger the phosphorylation of eIF2α, which is a hallmark of the integrated stress response (Tian et al., 2021). Phosphorylation of eIF2α is also highly associated with immunogenic cell death, and specifically with the cell surface expression of calreticulin (Bezu et al., 2018). In line with this, our results demonstrated that the MPMs could induce both the phosphorylation of eIF2α and the cell surface expression of calreticulin in HSC-3 and UT-SCC-24A cells. On the contrary, cisplatin also induced high levels of phosphorylated eIF2α, but no cell surface calreticulin. This is in line with what has been demonstrated in previous studies (Bezu et al., 2018). The MPMs also induced release of ATP and HMGB1 from HSC-3 and UT-SCC-24A cells. Taken together, all of these results suggest that the MPMs may be able to induce immunogenic cell death.

Immunogenic cell death is considered a distinct form of cell death, which is defined by its ability to activate an adaptive immune response (Galluzzi et al., 2018). However, it can appear in different forms. Anthracyclines, which are a group of DNA-intercalating chemotherapeutic agents that are known for their ability to induce immunogenic cell death, trigger a form of immunogenic apoptosis (Obeid et al., 2007). Other compounds, for instance the amphipathic and oncolytic peptide LTX-315, induces a form of immunogenic cell death which is more reminiscent of necrosis (Zhou et al., 2016). Historically, necrosis is known as a proinflammatory type of cell death as the membrane rupture which occurs during necrosis causes the release of DAMPs, such as HMGB1 (Scaffidi et al., 2002).

While the activation of caspase 3/7 that was observed in the live cell apoptosis assay as well as the partial protection of cells from MPM induced cell death by the pan-caspase inhibitor z-VAD-FMK indicates that the MPMs can trigger apoptosis, there are however other factors that suggest that apoptosis is not the main type of cell death induced by the MPMs. For instance, the increase in viability caused by z-VAD-FMK was generally small and statistically insignificant in many cases. In addition, the morphology of the MPM treated cells was not typical for apoptotic cells. The flow cytometric analysis of externalization of PS did not indicate that the cells were undergoing apoptosis. This could be seen by the fact that there was no Annexin V+/PI–population in the MPM treated cells, as there was in the cisplatin treated cells. While some of the MPM treated HSC-3 and UT-SCC-24A cells, especially those treated with MPM-2:0, did appear to have been apoptotic as their PI intensity was relatively low, none of these cells were completely negative for PI, suggesting that membrane rupture happens early during cell death, which is not typical for apoptotic cells. The presence of a large population of Annexin V–/PI+ cells, which was especially prominent in UT-SCC-24A cells treated with MPM-3:2 and MPM-4:2, also supports this notion. Taken together, these results indicate that apoptosis is not the main mode of cell death induced by the MPMs.

Historically, apoptosis and necrosis were known as the two main modes of cell death, representing regulated and accidental cell death, respectively. However, it is now accepted that a variety of different cell death modes exist (Galluzzi et al., 2018). For example, different forms of regulated necrosis exist as well (Galluzzi et al., 2018). The fact that BafA1 and, to a certain extent, z-VAD-FMK, could increase the viability of MPM treated cells indicates that the death that they induce is regulated. The cell surface expression of calreticulin, which is regulated by the phosphorylation of eIF2α, and the activation of caspase 3/7 also indicate the activation of regulated pathways. As previously mentioned, phosphorylation of eIF2α is a reaction to cellular stress, specifically to endoplasmic reticulum stress and the misfolding of proteins (Hetz et al., 2020). This in turn may cause the activation of caspases and subsequent apoptosis. However, as discussed, it seems like the MPMs do not induce classic apoptosis.

Taken together, this study has demonstrated that amphipathic barbiturates effectively reduce the viability of HNSCC cancer cells by inducing a particular form of cell death, which may be coupled to lysosomotropism. All of the four most potent compounds were able to induce cell surface expression of calreticulin and release of ATP. MPM-2:0 and MPM-6:0 also caused release of HMGB1. These are important hallmarks for immunogenic cell death and therefore makes the MPMs interesting to study further. By making small changes to the structure of the MPMs, their potency could be modified. This is of great interest when designing and selecting compounds for future *in vivo* studies. However, potency is not the only important feature of an anti-cancer compound. Despite MPM-3:2 and MPM-4:2 being somewhat less potent than MPM-2:0 and MPM-6:0, they had a considerably lower effect on red blood cells and bacteria. Therefore, MPM-3:2 and MPM-4:2 may be the most attractive compounds for inclusion in future studies.

## Data Availability

The original contributions presented in the study are included in the article/Supplementary Material, further inquiries can be directed to the corresponding authors.
