# Supplementary material for: Amphipathic barbiturates as marine product mimics with cytolytic and immunogenic effects on head and neck squamous cell carcinoma cell lines
Source: Front Pharmacol. 2023 Mar 30;14:1141669. doi: 10.3389/fphar.2023.1141669 (PMC10098121; doi:10.3389/fphar.2023.1141669)
Supplement: Supplementary file 1 [file DataSheet2.pdf]

Supplementary Material

Supplementary Table S1: Clinical and pathological characteristics of the cancer cell lines. TNM is based on pathology reports.

CELL LINE	SEX[A]	AGE[B]	TNM	SPECIMEN SITE	TYPE[C]	GRADE	REF
**UT-SCC-8**	M	42	T2N0M0	larynx	pri	G1	LE* Uni. Turku
**UT-SCC-24A**	M	41	T2N0M0	tongue	pri	G2	LE* Uni. Turku
**UT-SCC-24B**	M	41	T2N1M0	neck	met(per)	G2	LE* Uni. Turku
**UT-SCC-42A**	M	43	T4N3M0	larynx	pri	G3	LE* Uni. Turku
**UT-SCC-42B**	M	43	T4N3M0	neck	met	G3	LE* Uni. Turku
**UT-SCC-106**	M	37	T1AN0M	larynx	pri	G1	LE* Uni. Turku
**HSC-3**	M	64		tongue	met		JCRB Cell Bank

[A]M=MALE, F=FEMALE, [B] AGE IN YEARS, [C] PRI=PRIMARY TUMOR, MET=METASTASIS, PER= PERSISTENT DISEASE, *LOCALLY ESTABLISHED

Supplementary Table S2. Overview of the antimicrobial activity of all MPMs given by their MIC in µg/mL.

Core structure	Comp. ID	R	Antimicrobial activity S. a	B. s	E. c	P. a
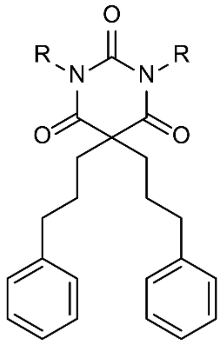	**MPM-1**	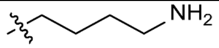				
	**MPM-2:0**	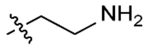	8	8	16	16
	**MPM-3:0**	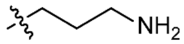	32	16	64	64
	**MPM-5:0**	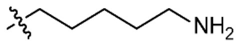	8	4	32	32
	**MPM-6:0**	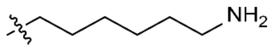	4	2	8	8
	**MPM-4:1**	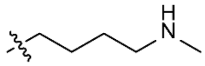	32	8	>64	>64
	**MPM-4:2**	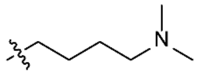	64	16	32	>64
	**MPM-4:3**	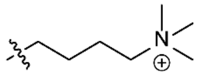	64	>64	64	>64
	**MPM-2:2**	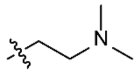	>64	32	32	>64
	**MPM-3:2**	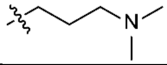	>64	16	32	>64

Bacterial reference strains: S. a – *Staphylococcus aureus* ATCC 9144, B.s – *Bacillus subtilis* 168, E. c – *Escherichia coli* ATCC 25922, and P. a – *Pseudomonas aeruginosa* ATCC 27853. All compounds were tested as di-TFA salts.

Supplementary Table S3. Calculated drug sensitivity scores (DSS) for the MPMs on HNSCC cell lines. The data was obtained from the high throughput drug screening.

	HSC-3	UT-SCC-8	UT-SCC-24A	UT-SCC-24B	UT-SCC-42A	UT-SCC-42B	UT-SCC-106A	NOF
**MPM-2:0**	19	19,2	18,7	18,3	19	18,7	18	18,3
**MPM-6:0**	17,8	19,5	18,6	18,3	17,9	18,4	17,9	18
**MPM-4:2**	18,2	18,1	18,4	18,9	17	16,6	19,5	22,6
**MPM-3:2**	17,9	17,8	17,9	18,4	16	15,7	18,6	19,3
**MPM-2:2**	16,8	17	18,1	17,8	13,4	12,9	18,6	19,2
**MPM-5:0**	17,6	18,1	17,4	18,2	13,5	11,5	17,9	19
**MPM-3:0**	16,1	16,5	14,3	18	11,7	10,1	17,1	18,3
**MPM-4:1**	14,8	14,5	15,5	16,4	9,9	10,2	15,8	18,5
**Cisplatin**	15,6	16,8	8,9	11,6	7,1	7,7	9	9,6
**MPM-1**	8,8	10	6,9	9,7	8,4	7,4	10,3	17,1
**MPM-4:3**	0	0	0	0	0,1	0,1	0,5	2,2

Supplementary Table S4. Combined treatment of cells with MPMs and irradiation does not have synergistic or antagonistic effects. The degree of synergy/antagonism was calculated by subtracting the drug sensitivity score (DSS) in non-irradiated cells from the DSS in irradiated (2 Gy) cells, giving a delta DSS (ΔDSS). ΔDSS >5 indicates synergy, while ΔDSS<–5 indicates antagonism.

	HSC-3	UT-SCC-8	UT-SCC-24A	UT-SCC-24B	UT-SCC-42A	UT-SCC-42B	UT-SCC-106A	NOF
**MPM-2:0**	-0,7	0,6	-0,7	-0,1	-0,7	-0,1	-0,5	-0,7
**MPM-6:0**	0,7	-0,8	-0,2	0,1	0,3	-0,5	-0,4	-0,3
**MPM-4:2**	0,3	0,5	-0,6	-0,5	0,4	-0,4	-0,2	1,2
**MPM-3:2**	-0,2	0,1	-0,2	0,1	0,6	0,3	-0,2	-1,6
**MPM-2:2**	1,4	0,2	-0,4	-1,5	0,9	-0,4	-1,5	-1,2
**MPM-5:0**	0,2	0,4	-1,2	-0,5	-0,8	-0,5	-1	-1,2
**MPM-3:0**	0	-0,6	-1,8	-0,6	-1,1	-0,1	-0,6	-0,6
**MPM-4:1**	-0,4	0,6	-1,6	-0,5	-0,1	-1,1	-0,8	-0,8
**Cisplatin**	-0,8	-0,8	-0,9	0,7	-0,1	-0,6	0,3	-1,9
**MPM-1**	0	-0,8	-1,1	0,5	-0,8	0,6	0,3	0,4
**MPM-4:3**	0	0	0	0,1	0	-0,1	-0,5	-1,4

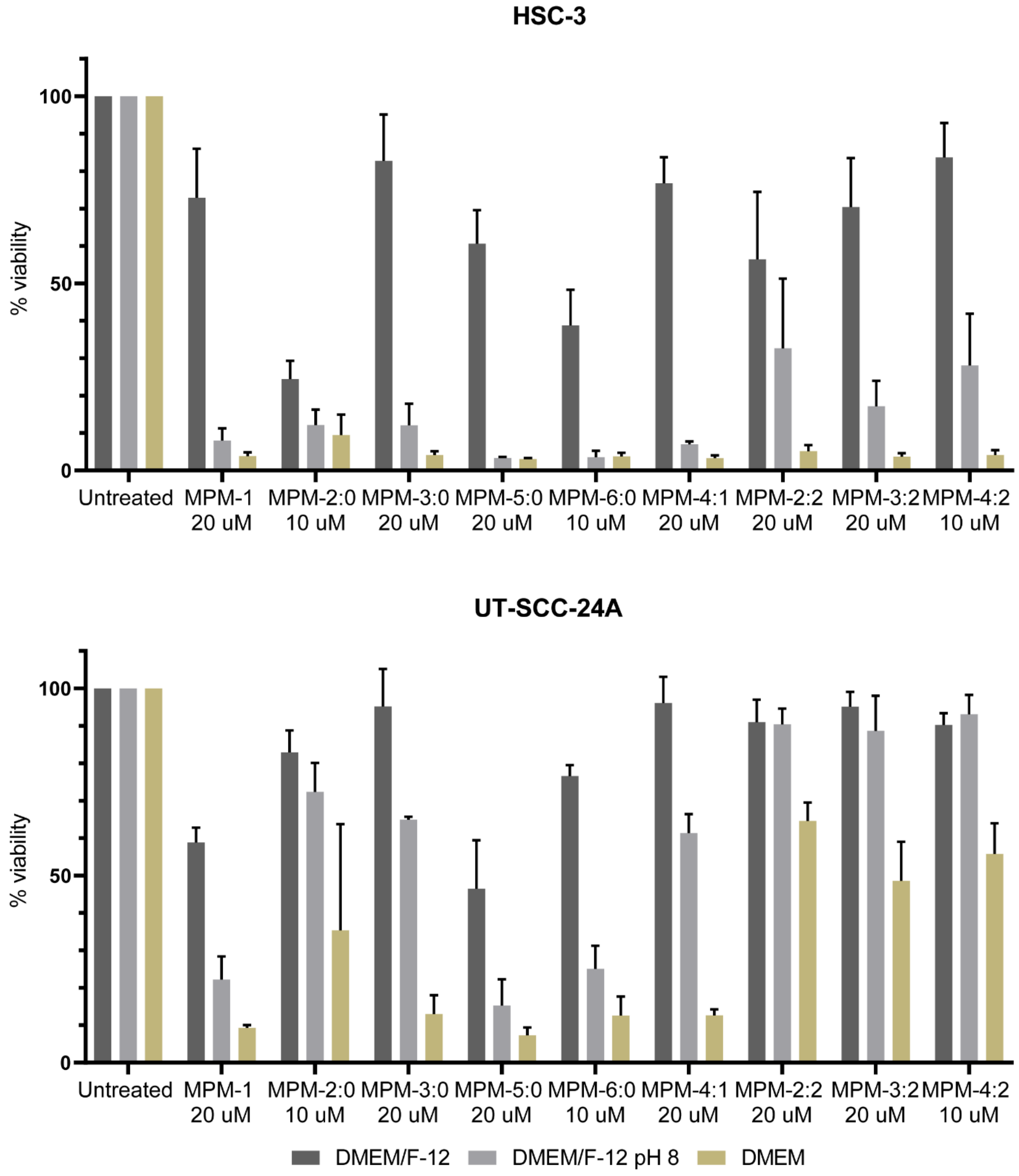

Supplementary Figure S1. The viability of HSC-3 and UT-SCC-24A cells treated with MPMs in different cell culture medias was measured by means of the MTS assay. The cells were seeded in 96-well plates (15 000 cells/well) and treated with MPMs for four hours the following day at 10 or 20 µM according to their potency. The cells were treated in DMEM/F-12, DMEM/F-12 pH 8 (pH adjusted by the addition of NaOH) or DMEM.

Supplementary Methods

Method for determination of bacterial minimum inhibitory concentration (MIC)

Stock solutions of the water-soluble compounds were prepared by dissolving them in ultrapure water (Milli-Q H_2O, Millipore, MA, USA). The less water-soluble compounds were first dissolved in 25 - 50 µL 100% DMSO before further dilution with ultrapure water. The DMSO concentration was always less than 1% in the working concentration of each compound. A modified broth microdilution susceptibility test [1] based on the CLSI M07-A9 protocol, [2] was used to determine minimal inhibitory concentrations (MIC). Briefly, the test compounds were two-fold diluted with ultrapure water in polystyrene 96-well flat-bottom microplates (NUNC, Roskilde, Denmark). The bacterial inoculum was diluted to 2.5 - 3 x 10^4 cells/mL in Mueller-Hinton broth (MHB, Difco Laboratories, USA) and added to the different diluted compounds in a ratio of 1:1. Positive control (ciprofloxacin, Sigma-Aldrich, USA), negative control (bacteria + water), and media control (media + water) were included in each experiment. The microplates were incubated for 48 h at 35 °C in an EnVision microplate reader (PerkinElmer, Waltham, MA, USA). The lowest concentration of compounds that caused no bacterial growth, as determined by optical density (OD_{600}) measurements, was defined as the MIC value. All compounds were tested in 3 technical replicates.

Supplementary Video S1. HSC-3 cells were seeded at 100% confluence and a standard scratch wound migration assay was performed. The cells were imaged every 2 hours for 24 hours. The video shows untreated cells (left) and cells treated with 10 µM MPM-4:2 (right).

References

1. Igumnova EM, Mishchenko E, Haug T, Blencke HM, Sollid JUE, Fredheim EGA, et al. Synthesis and antimicrobial activity of small cationic amphipathic aminobenzamide marine natural product mimics and evaluation of relevance against clinical isolates including ESBL-CARBA producing multi-resistant bacteria. Bioorg Med Chem. 2016;24(22):5884-94.

2. Cockerill FR. Methods for Dilution Antimicrobial Susceptibility Tests for Bacteria That Grow Aerobically. Approved Standard. M07-A9 In: Institut CaLS, editor. 9th ed: Wayne, Pa : Clinical and Laboratory Standards Institut; 2012.
