# Supplementary material for: Amphipathic barbiturates as marine product mimics with cytolytic and immunogenic effects on head and neck squamous cell carcinoma cell lines
Source: Front Pharmacol. 2023 Mar 30;14:1141669. doi: 10.3389/fphar.2023.1141669 (PMC10098121; doi:10.3389/fphar.2023.1141669)
Supplement: Supplementary file 2 [file DataSheet1.pdf]

Supplementary Material

1 Experimental procedures

1.1 General information

Unless otherwise noted, purchased chemicals were used as received without further purification. Solvents were dried according to standard procedures over molecular sieves of appropriate size. Normal phase flash chromatography was carried out on silica gel 60 (230−400 mesh) or on an interchim ® PuriFlash XS420 flash system with the sample preloaded on a Samplet® cartridge belonging to a Biotage SP-1 system. Purification by reversed phase (RP) C18 column chromatography (H_2O with 0.1 % TFA/MeCN with 0.1 % TFA) was performed on an interchim® PuriFlash XS420 flash system with the sample preloaded on a Samplet® cartridge. Thin layer chromatography was carried out using Merck TLC Silica gel 60 F254 and visualized by short-wavelength ultraviolet light or by treatment with an appropriate stain.

NMR spectra were obtained on a 400 MHz Bruker Advance III HD spectrometer equipped with a 5 mm SmartProbe BB/1H (BB = 19F, 31P-15N) at 20 °C. The chemical shifts are reported in ppm relative to the solvent residual peak ($CDCl_3$: δH 7.26 and δC 77.16; Methanol-d4: δH 3.31 and δC 49.00; deuterium oxide: δH 4.79; DMSO-d6 δH 2.51 and δC 39.52). ^{13}C NMR spectra were obtained with ^{1}H decoupling. Data are represented as follows: chemical shift, multiplicity (s = singlet, d = doublet, t = triplet, s = septet, m = multiplet), coupling constant (*J* in Hz) and integration. The raw data was analyzed with MestReNova (Version 14.0.0-23239).

High-resolution mass spectra (HRMS) were recorded from methanol solutions on an LTQ Orbitrap XL (Thermo Scientific) either in negative or in positive electrospray ionization (ESI) mode. The data was analyzed with the Thermo Scientific Xcalibur software.

The purity of all tested compounds was determined to be ≥95%. The analyses were carried out on a Waters ACQUITY UPC^2 system equipped with a Torus™ DEA 130Å, 1.7 μm, 2.1 mm x 50 mm column or a Torus™ 2-PIC 130Å, 1.7 μm, 2.1 mm x 50 mm column. Compounds were detected on a Waters ACQUITY PDA detector spanning wavelengths from 190 to 650 nm, coupled to a Waters ACQUITY QDA detector for low resolution mass (LRMS) detection. The derivatives were eluted with a mobile phase consisting of supercritical CO_2 and MeOH containing 0.1 % NH_3 and a linear gradient of 2 – 40% MeOH over 2 or 4 min followed by isocratic 0.5 min of 40% MeOH. The flow rate was 1.5 mL/min.

Dialkylated barbituric acid **1** was obtained following the procedures described by von Hofsten et al.[1]

<u>*General Procedure A:*</u> *N,N'*-dialkylation via Mitsunobu reaction

The respective 5,5-disubstituted barbituric acid, *N*-Boc amino alcohol and PPh_3 were mixed with anhydrous DCM in a heat dried vial under an argon atmosphere. The suspension was cooled to 0 °C and upon dropwise addition of DIAD a clear yellow solution was obtained. The mixture was left

stirring in the melting ice-water bath until TLC indicated full conversion. Then 10 % $NaHCO_{3(aq)}$ solution and EtOAc were added and the layers were separated. The aqueous layer was extracted twice with EtOAc and the combined organics were dried over Na_2SO_4, filtered and the solvent was removed under reduced pressure. The crude product was purified by column chromatography on silica gel with EtOAc/heptane as solvents to yield the *N,N'*-dialkylated barbiturates.
To the *N,N'*-dialkylated barbiturates dissolved in DCM, was added TFA and the mixture was stirred at ambient temperature until HRMS indicated full conversion. The solvent was removed and the crude product was purified on an automated flash system equipped with a C18 column and MeCN/H_2O containing 0.1% TFA as solvents. The product containing fractions were collected, the solvent was removed and the product was lyophilized for 48 h. The obtained solids were triturated three times with Et_2O. The solids were dissolved in MeOH and water was added. The mixture was lyophilized for 48 h to yield the desired amines as di-TFA salts in ≥95% purity.

1.2 Synthesis of intermediates

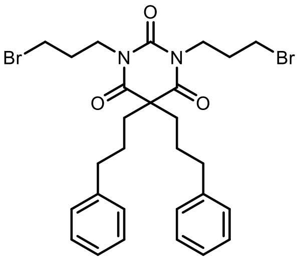

1,3-bis(3-bromopropyl)-5,5-bis(3-phenylpropyl)pyrimidine-2,4,6(1H,3H,5H)-trione **2**.
Compound **1** (100 mg, 0.27 mmol, 1.0 eq) and Cs_2CO_3 (223.5 mg, 0.69 mmol, 2.5 eq) were mixed with acetone (1 mL) and the suspension was stirred for 10 min at ambient temperature. 1,3-dibromopropane (98 µL, 0.96 mmol, 3.5 eq) was added and the suspension was heated to 60 °C for 42 h. The suspension was allowed to cool to ambient temperature, EtOAc and water were added, and the layers were separated. The aqueous layer was extracted with EtOAc twice. The combined organics were dried over $MgSO_4$, filtered and the solvent was removed. After purification by column chromatography on silica gel with 15% EtOAc in heptane compound **2** (113 mg, 0.19 mmol, 68%) was obtained as a colorless oil.

^{1}H NMR (400 MHz, Chloroform-*d*) δ 7.30 – 7.22 (m, 4H), 7.21 – 7.14 (m, 2H), 7.10 – 7.04 (m, 4H), 4.01 (t, *J* = 7.2 Hz, 3H), 3.37 (t, *J* = 6.6 Hz, 4H), 2.53 (t, *J* = 7.7 Hz, 4H), 2.14 (s, *J* = 6.9 Hz, 4H), 2.06 – 1.97 (m, 4H), 1.42 – 1.31 (m, 4H). **^{13}C NMR** (101 MHz, Chloroform-*d*) δ 171.6 (2C), 150.6, 141.0 (2C), 128.7 (4C), 128.4 (4C), 126.3 (2C), 56.7, 41.3 (2C), 39.6 (2C), 35.7 (2C), 31.1 (2C), 29.9 (2C), 27.1 (2C). **HRMS** (ESI): calcd for $C_{28}H_{34}Br_2N_2O_3Na^+$ $[M+H]^+$ 627.0828, found: 627.0821.
Note: Chloroform-d signal overlaps with phenyl rings.

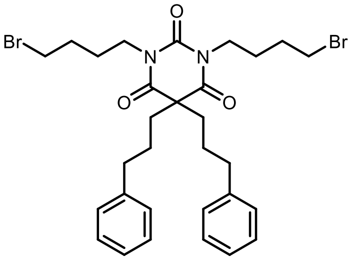

1,3-bis(4-bromobutyl)-5,5-bis(3-phenylpropyl)pyrimidine-2,4,6(1H,3H,5H)-trione **3**.
Compound **1** (243 mg, 0.67 mmol, 1.0 eq) and Cs_2CO_3 (543.1 mg, 1.67 mmol, 2.5 eq) were mixed with acetone (1 mL) and the suspension was stirred for 10 min at ambient temperature. 1,4-dibromobutane (339 µL, 2.33 mmol, 3.5 eq) was added and the suspension was heated to 60 °C for 48 h. The suspension was allowed to cool to ambient temperature, EtOAc and water were added, and the layers were separated. The aqueous layer was extracted with EtOAc twice. The combined organics were dried over $MgSO_4$, filtered and the solvent was removed. After purification on an automated flash system

equipped with a silica column and gradient 0-25% EtOAc in heptane compound **3** (364 mg, 0.57 mmol, 86%) was obtained as a colorless oil.

^{1}**H NMR** (400 MHz, Chloroform-*d*) δ 7.30 – 7.23 (m, 4H), 7.21 – 7.14 (m, 2H), 7.11 – 7.06 (m, 4H), 3.90 (t, *J* = 7.2 Hz, 4H), 3.39 (t, *J* = 6.5 Hz, 4H), 2.54 (t, *J* = 7.7 Hz, 4H), 2.06 – 1.98 (m, 4H), 1.90 – 1.81 (m, 4H), 1.78 – 1.68 (m, 4H), 1.43 – 1.33 (m, 4H). ^{13}**C NMR** (101 MHz, Chloroform-*d*) δ 171.7 (2C), 150.6, 141.1 (2), 128.6 (4C), 128.4 (4C), 126.3, 56.6, 41.3 (2C), 39.8 (2C), 35.7 (2C), 32.8 (2C), 30.1 (2C), 27.1 (2C), 26.0 (2C). **HRMS** (ESI): calcd for $C_{30}H_{39}Br_2N_2O_3^+$ [M+H]$^+$ 633.1322, found: 633.1329.
Note: Chloroform-d signal overlaps with signals originating from the phenyl rings.

1.3 Synthesis of final MPMs

The following compounds were prepared according to General Procedure A

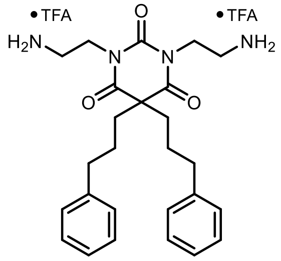

1,3-bis(2-aminoethyl)-5,5-bis(3-phenylpropyl)pyrimidine-2,4,6(1H,3H,5H)-trione **MPM-2:0**.

Compound **1** (75 mg, 206 µmol, 1.0 eq), *tert*-butyl (2-hydroxyethyl)carbamate (83 mg, 515 µmol, 2.5 eq), PPh_3 (162 mg, 617 µmol, 3.0 eq) and DIAD (129 µL, 617 µmol, 3.0 eq) were stirred in anhydrous DCM (1.0 mL) for 4 h. The crude was purified with 10-50% EtOAc in heptane to yield impure Boc-**MPM-2:0** (127 mg, 195 µmol, 95%) as a white solid.

TFA (315 µL, 4.11 mmol, 20.0 eq) and DCM (1.0 mL) were added and the solution was stirred at ambient temperature for 22 h. The crude was purified by RP chromatography with a gradient of 15-50% MeCN in H_2O (both containing 0.1% TFA) to yield the di-TFA salt of **MPM-2:0** (71 mg, 105 µmol, 51% o2s) as a white solid.

^{1}**H NMR** (400 MHz, Methanol-*d4*) δ 7.27 – 7.19 (m, 4H), 7.18 – 7.07 (m, 7H), 4.18 (t, *J* = 5.7 Hz, 4H), 3.21 (t, *J* = 5.7 Hz, 4H), 2.54 (t, *J* = 7.2 Hz, 4H), 1.96 – 1.87 (m, 4H), 1.54 – 1.42 (m, 4H). ^{13}**C NMR** (101 MHz, Methanol-*d4*) δ 172.9 (2C), 153.1, 142.5 (2C), 129.4 (8C), 127.0 (2C), 57.8, 41.0 (2C), 39.5 (2C), 38.7 (2C), 36.4 (2C), 27.3 (2C). **HRMS** (ESI): calcd for $C_{26}H_{35}N_4O_3^+$ [M+H]$^+$ 451.2704, found 451.2694. **SFC**: >99%.

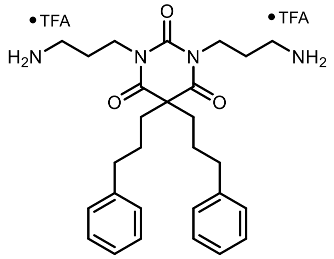

1,3-bis(3-aminopropyl)-5,5-bis(3-phenylpropyl)pyrimidine-2,4,6(1H,3H,5H)-trione **MPM-3:0**.

Compound **1** (75 mg, 206 µmol, 1.0 eq), *tert*-butyl (3-hydroxypropyl)carbamate (90 mg, 515 µmol, 2.5 eq), PPh_3 (162 mg, 617 µmol, 3.0 eq) and DIAD (129 µL, 617 µmol, 3.0 eq) were stirred in anhydrous DCM (1.0 mL) for 4 h. The crude was purified with 10-50% EtOAc in heptane to yield impure Boc-**MPM-3:0** (132 mg, 194 µmol, 95%) as a white solid.

TFA (315 µL, 4.11 mmol, 20.0 eq) and DCM (1.0 mL) were added and the solution was stirred at ambient temperature for 22 h. The crude was purified by RP chromatography with a gradient of 15-50% MeCN in H_2O (both containing 0.1% TFA) to yield the di-TFA salt of **MPM-3:0** (133 mg, 188 µmol, 92% o2s) as a white solid.

^{1}H NMR (400 MHz, Methanol-*d4*) δ 7.28 – 7.20 (m, 4H), 7.24 (dd, *J* = 8.1, 6.7 Hz, 4H), 7.18 – 7.12 (m, 2H), 3.98 (t, *J* = 7.1 Hz, 4H), 2.96 (t, *J* = 7.4 Hz, 3H), 2.55 (t, *J* = 7.4 Hz, 4H), 2.03 – 1.88 (m, 8H), 1.42 (dtd, *J* = 14.9, 7.6, 4.3 Hz, 4H). **^{13}C NMR** (101 MHz, Methanol-*d4*) δ 173.0 (2C), 152.0, 142.4 (2C), 129.4 (4C), 129.3 (4C), 127.1 (2C), 57.7, 40.1 (2C), 40.0 (2C), 38.4 (2C), 36.4 (2C), 27.8 (2C), 27.3 (2C).**HRMS** (ESI): calcd for $C_{28}H_{39}N_4O_3^+$ [M+H]$^+$ 479.3017, found 479.3011. **SFC**: >99%.

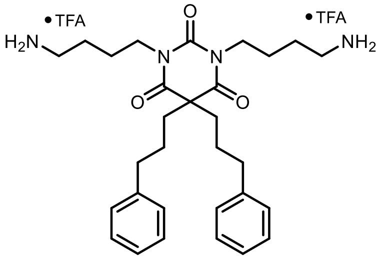

1,3-bis(4-aminobutyl)-5,5-bis(3-phenylpropyl)pyrimidine-2,4,6(1H,3H,5H)-trione **MPM-1**.

Compound **1** (500 mg, 1.37 mmol, 1.0 eq), *tert*-butyl (4-hydroxybutyl)carbamate (597 mg, 3.16 mmol, 2.3 eq), PPh_3 (1.08 g, 4.12 mmol, 3.0 eq) and DIAD (862 µL, 4.12 mmol, 3.0 eq) were stirred in anhydrous DCM (4.0 mL) for 22 h. The crude was purified with 10-50% EtOAc in heptane to yield impure Boc-**MPM-1** (906 mg, 1.28 mmol, 93%) as a white solid.

TFA (2.10 mL, 27.4 mmol, 20.0 eq) and DCM (4.0 mL) were added and the solution was stirred at ambient temperature for 22 h. The crude was purified by RP chromatography with a gradient of 15-50% MeCN in H_2O (both containing 0.1% TFA) to yield the di-TFA salt of **MPM-1** (586 mg, 798 µmol, 58% o2s) as a white solid.

^{1}H NMR (400 MHz, Methanol-*d4*) δ 7.28 – 7.20 (m, 4H), 7.19 – 7.12 (m, 2H), 7.12 – 7.07 (m, 4H), 3.96 – 3.85 (m, 4H), 2.98 – 2.89 (m, 4H), 2.54 (t, *J* = 7.4 Hz, 4H), 2.02 – 1.90 (m, 4H), 1.65 (p, *J* = 3.8 Hz, 8H), 1.47 – 1.34 (m, 4H). **^{13}C NMR** (101 MHz, Methanol-*d4*) δ 172.9 (2C), 151.9, 142.5 (2C), 129.4 (4C), 129.3 (4C), 127.1 (2C), 57.6, 42.2 (2C), 40.2 (4C), 36.4 (2C), 27.9 (2C), 26.0 (2C), 25.9 (2C). **HRMS** (ESI): calcd for $C_{30}H_{43}N_4O_3^+$ [M+H]$^+$ 507.3330, found 507.3324. **SFC:** 96.3%.

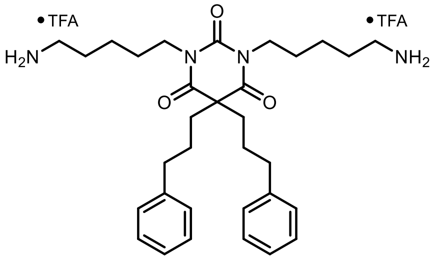

1,3-bis(5-aminopentyl)-5,5-bis(3-phenylpropyl)pyrimidine-2,4,6(1H,3H,5H)-trione **MPM-5:0**\.

Compound **1** (75 mg, 206 µmol, 1.0 eq), *tert*-butyl (5-hydroxypentyl)carbamate (105 mg, 515 µmol, 2.5 eq), PPh_3 (162 mg, 617 µmol, 3.0 eq) and DIAD (129 µL, 617 µmol, 3.0 eq) were stirred in anhydrous DCM (1.0 mL) for 4 h. The crude was purified with 10-45% EtOAc in heptane to yield impure Boc-**MPM-5:0** (185 mg, 252 µmol, 122%) as a colorless solid.

TFA (315 µL, 4.11 mmol, 20.0 eq) and DCM (1.0 mL) were added and the solution was stirred at ambient temperature for 22 h. The crude was purified by RP chromatography with a gradient of 15-50% MeCN in H_2O (both containing 0.1% TFA) to yield the di-TFA salt of **MPM-5:0** (146 mg, 191 µmol, 93% o2s) as a white solid.

^{1}H NMR (400 MHz, Methanol-*d4*) δ 7.28 – 7.20 (m, 4H), 7.19 – 7.12 (m, 2H), 7.12 – 7.06 (m, 4H), 3.93 – 3.85 (m, 4H), 2.93 – 2.81 (m, 4H), 2.53 (t, *J* = 7.5 Hz, 4H), 2.01 – 1.89 (m, 4H), 1.74 – 1.53 (m, 8H), 1.45 – 1.33 (m, 8H). **^{13}C NMR** (101 MHz, Methanol-*d4*) δ 173.0 (2C), 151.8, 142.5, 129.5 (4C), 129.3 (4C), 127.1 (2C), 57.5, 42.5 (2C), 40.5 (2C), 40.4 (2C), 36.5 (2C), 28.4 (2C), 28.0 (4C), 24.7 (2C). **HRMS** (ESI): calcd for $C_{32}H_{47}N_4O_3^+$ [M+H]$^+$ 535.3643, found 535.3637. **SFC:** >99%.

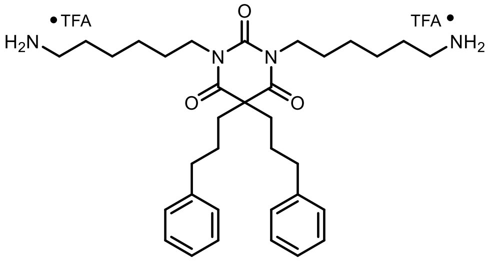

1,3-bis(6-aminohexyl)-5,5-bis(3-phenylpropyl)pyrimidine-2,4,6(1H,3H,5H)-trione **MPM-6:0**.

Compound **1** (75 mg, 206 µmol, 1.0 eq), *tert*-butyl (6-hydroxyhexyl)carbamate (112 mg, 515 µmol, 2.5 eq), PPh_3 (162 mg, 617 µmol, 3.0 eq) and DIAD (129 µL, 617 µmol, 3.0 eq) were stirred in anhydrous DCM (1.0 mL) for 4 h. The crude was purified with 10-45% EtOAc in heptane to yield impure Boc-**MPM-6:0** (161 mg, 211 µmol, 103%) as a colorless oil.

TFA (315 µL, 4.11 mmol, 20.0 eq) and DCM (1.0 mL) were added and the solution was stirred at ambient temperature for 22 h. The crude was purified by RP chromatography with a gradient of 15-50% MeCN in H_2O (both containing 0.1% TFA) to yield the di-TFA salt of **MPM-6:0** (120 mg, 152 µmol, 74% o2s) as a white solid.

^{1}H NMR (400 MHz, Methanol-*d4*) δ 7.28 – 7.20 (m, 4H), 7.19 – 7.12 (m, 2H), 7.11 – 7.04 (m, 4H), 3.91 – 3.84 (m, 4H), 2.93 – 2.84 (m, 4H), 2.53 (t, *J* = 7.5 Hz, 4H), 1.99 – 1.89 (m, 4H), 1.67 – 1.51 (m, 8H), 1.46 – 1.31 (m, 12H). **^{13}C NMR** (101 MHz, Methanol-*d4*) δ 173.0 (2C), 151.9, 142.5 (2C), 129.5 (4C), 129.3 (4C), 127.1 (2C), 57.5, 42.7 (2C), 40.6 (2C), 40.5 (2C), 36.5 (2C), 28.7 (2C), 28.4 (2C), 28.0 (2C), 27.4 (2C), 26.9 (2C). **HRMS** (ESI): calcd for $C_{34}H_{51}N_4O_3^+$ $[M+H]^+$ 563.3956, found 563.3950. **SFC:** >99%.

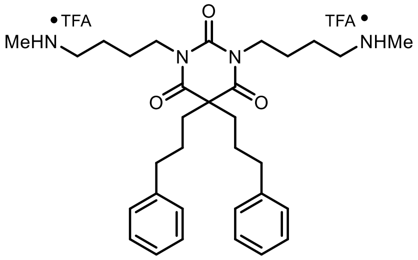

1,3-bis(4-(methylamino)butyl)-5,5-bis(3-phenylpropyl)pyrimidine-2,4,6(1H,3H,5H)-trione **MPM-4:1**.

Compound **3** (86 mg, 136 µmol, 1.0 eq) was taken up in anhydrous acetonitrile (1 mL) and methylamine (542 µL, 1.08 mmol, 8.0 eq; 2M in THF) was added. After heating to 70 °C for 40 h, the mixture was allowed to cool to ambient temperature. The solvent was removed and crude was purified on an automated flash system equipped with a C18 column and gradient 10-55% MeCN in H_2O (both containing 0.1% TFA). The di-TFA salt of **MPM-4:1** (65 mg, 85 µmol, 63%) was obtained as a white solid.

^{1}H NMR (400 MHz, Methanol-*d4*) δ 7.28 – 7.20 (m, 4H), 7.19 – 7.12 (m, 2H), 7.11 – 7.06 (m, 4H), 3.90 (t, *J* = 6.8 Hz, 4H), 3.00 (t, *J* = 7.2 Hz, 4H), 2.67 (s, 6H), 2.54 (t, *J* = 7.4 Hz, 4H), 1.99 – 1.90 (m, 4H), 1.74 – 1.57 (m, 8H), 1.46 – 1.32 (m, 4H). **^{13}C NMR** (101 MHz, Methanol-*d4*) δ 172.9 (2C), 151.9, 142.5 (2C), 129.5 (4C), 129.3 (4C), 127.1 (2C), 57.6, 49.7 (2C) 42.2 (2C), 40.3 (2C), 36.4 (2C), 33.5 (2C), 27.9 (2C), 26.0 (2C), 24.4 (2C). **HRMS** (ESI): calcd for $C_{32}H_{47}N_4O_3^+$ $[M+H]^+$ 535.3643, found: 535.3646. **SFC:** 96.2%.

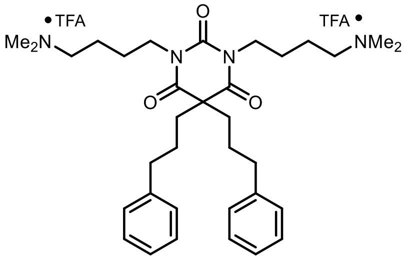

1,3-bis(4-(dimethylamino)butyl)-5,5-bis(3-phenylpropyl)pyrimidine-2,4,6(1H,3H,5H)-trione **MPM-4:2**.

Compound **3** (82 mg, 129 µmol, 1.0 eq) was taken up in anhydrous acetonitrile (1 mL) and dimethylamine (517 µL, 1.03 mmol, 8.0 eq; 2 M in THF) was added. After heating to 70 °C for 40 h, the mixture was allowed to cool to ambient temperature. The solvent was removed and crude was purified on an automated flash system equipped with a C18 column and gradient 10-55% MeCN in H_2O (both containing 0.1% TFA). The di-TFA salt of **MPM-4:2** (99 mg, 125 µmol, 97%) was obtained as a white solid, which became a colorless oil upon standing.

^{1}H NMR (400 MHz, Methanol-*d4*) δ 7.28 – 7.20 (m, 4H), 7.19 – 7.12 (m, 2H), 7.12 – 7.07 (m, 4H), 3.92 (t, *J* = 7.1 Hz, 4H), 3.17 – 3.08 (m, 4H), 2.83 (s, 12H), 2.54 (t, *J* = 7.4 Hz, 4H), 2.00 – 1.91 (m, 4H), 1.77 – 1.57 (m, 8H), 1.46 – 1.33 (m, 4H). **^{13}C NMR** (101 MHz, Methanol-*d4*) δ 172.9 (2C), 151.9, 142.5 (2C), 129.5 (4C), 129.3 (4C), 127.1 (2C), 58.3 (2C), 57.6, 43.4 (4C), 42.1 (2C), 40.3 (2C), 36.5 (2C), 28.0 (2C), 25.9 (2C), 22.9 (2C). **HRMS** (ESI): calcd for $C_{34}H_{51}N_4O_3^+$ $[M+H]^+$ 563.3956, found: 563.3956. **SFC:** >99%.

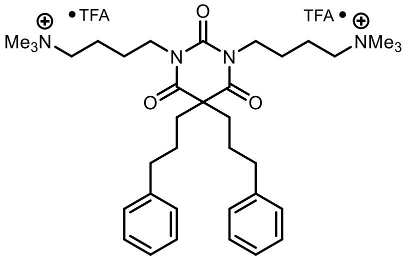

4,4'-(2,4,6-trioxo-5,5-bis(3-phenylpropyl)dihydropyrimi-dine-1,3(2H,4H)-diyl)bis(N,N,N-trimethylbutan-1-aminium) **MPM-4:3**.

Compound **3** (79 mg, 125 µmol, 1.0 eq) was taken up in anhydrous acetonitrile (1 mL) and trimethylamine (996 µL, 1.00 mmol, 8.0 eq; 1 M in THF) was added. After heating to 70 °C for 40 h, the mixture was allowed to cool to ambient temperature. The solvent was removed and crude was purified on an automated flash system equipped with a C18 column and gradient 10-55% MeCN in H_2O (both containing 0.1% TFA). The di-TFA salt of **MPM-4:3** (98 mg, 120 µmol, 96%) was obtained as a white solid, which became a colorless oil upon standing.

^{1}H NMR (400 MHz, Methanol-*d4*) δ 7.29 – 7.21 (m, 4H), 7.20 – 7.13 (m, 2H), 7.12 – 7.07 (m, 4H), 3.98 – 3.83 (m, 4H), 3.40 – 3.33 (m, 4H), 3.08 (s, 18H), 2.54 (t, *J* = 7.5 Hz, 4H), 2.03 – 1.93 (m, 4H), 1.83 – 1.71 (m, 4H), 1.70 – 1.59 (m, 4H), 1.47 – 1.34 (m, 4H). **^{13}C NMR** (101 MHz, Methanol-*d4*) δ 173.0 (2C), 151.8, 142.5 (2C), 129.5 (4C), 129.3 (4C), 127.2 (2C), 67.0 (t, *J* = 2.9 Hz, 2C), 57.6, 53.5 (t, *J* = 3.9 Hz, 6C), 42.1 (2C), 40.4 (2C), 36.5 (2C), 28.0 (2C), 25.7 (2C), 21.3 (2C). **HRMS** (ESI): calcd for $C_{36}H_{56}N_4O_3^{2+}$ $[M]^{2+}$ 296.2171, found: 296.2172. **SFC:** >99%.

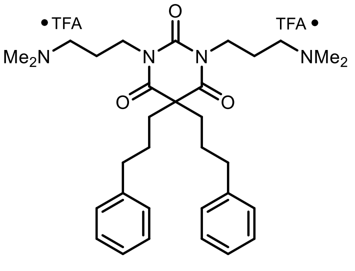

1,3-bis(3-(dimethylamino)propyl)-5,5-bis(3-phenylpropyl)-pyrimidine-2,4,6(1H,3H,5H)-trione **MPM-3:2**.

Compound **2** (113 mg, 186 µmol, 1.0 eq) was taken up in anhydrous acetonitrile (1 mL) and dimethylamine (745 µL, 1.49 mmol, 8.0 eq; 2 M in THF) was added. After heating to 70 °C for 24 h, the mixture was allowed to cool to ambient temperature. The solvent was removed and crude was purified on an automated flash system equipped with a C18 column and gradient 10-50% MeCN in H_2O (both containing 0.1% TFA). The di-TFA salt of **MPM-3:2** (135 mg, 177 µmol, 95%) was obtained as a white powder.

^{1}H NMR (400 MHz, Methanol-*d4*) δ 7.27 – 7.19 (m, 4H), 7.19 – 7.06 (m, 6H), 3.97 (t, *J* = 7.1 Hz, 4H), 3.18 – 3.07 (m, 4H), 2.83 (t, *J* = 1.4 Hz, 12H), 2.55 (t, *J* = 7.4 Hz, 4H), 2.08 – 1.89 (m, 8H), 1.49 – 1.36 (m, 4H). **^{13}C NMR** (101 MHz, Methanol-*d4*) δ 173.0 (2C), 151.9, 142.5 (2C), 129.5 (4C), 129.4 (4C), 127.1 (2C), 57.7, 56.4 (2C), 43.4 (4C), 40.0 (4C), 36.4 (2C), 28.0 (2C), 24.4 (2C). **HRMS** (ESI): calcd for $C_{32}H_{47}N_4O_3^+$ $[M+H]^+$ 535.3643, found: 535.3637. **SFC:** >99%.

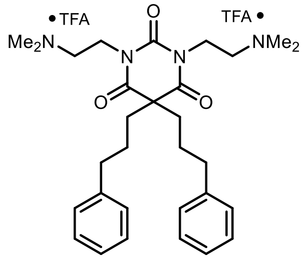

1,3-bis(2-(dimethylamino)ethyl)-5,5-bis(3-phenylpropyl)pyrimidine-2,4,6(1H,3H,5H)-trione **MPM-2:2**.

To **MPM-2:0** (29 mg, 43 µmol, 1.0 eq; TFA salt) and $NaBH_3CN$ (8.1 mg, 128 µmol, 3.0 eq) in MeOH (300 µL), were added acetic acid (9.8 µL, 171 µmol, 4.0 eq) and formaldehyde (19.2 µL, 256 µmol, 6.0 eq; 37% aqueous solution) and the resulting mixture was stirred at ambient temperature for 20 h. Saturated $NaHCO_{3(aq)}$ solution was added, the solvent was removed and the obtained salts were washed with MeOH. The combined organics were filtered over cotton wool and the solvent was removed. The crude was purified on an automated flash system equipped with a C18 column and gradient 15-50% MeCN in H_2O (both containing 0.1% TFA). The di-TFA salt of **MPM-2:2** (30 mg, 41 µmol, 96%) was obtained as a white foam.

^{1}H NMR (400 MHz, Methanol-*d4*) δ 7.27 – 7.19 (dd, *J* = 8.0, 6.6 Hz, 4H), 7.18 – 7.08 (m, 6H), 4.24 (t, *J* = 6.2 Hz, 4H), 3.40 (t, *J* = 6.1 Hz, 4H), 2.96 (s, 12H), 2.54 (t, *J* = 7.3 Hz, 4H), 1.99 – 1.89 (m, 4H), 1.56 – 1.43 (m, 4H). **^{13}C NMR** (101 MHz, Methanol-*d4*) δ 172.8 (2C), 152.7, 142.6 (2C), 129.4 (8C), 127.0 (2C), 57.8, 56.5 (2C), 43.9 (4C), 38.5 (2C), 38.4 (2C), 36.4 (2C), 27.4 (2C). **HRMS** (ESI): calcd for $C_{30}H_{43}N_4O_3^+$ $[M+H]^+$ 507.3330, found: 507.3326. **SFC:** *not obtained*.

2 NMR spectra

2.1 Starting materials for barbituric acid

1,3-bis(3-bromopropyl)-5,5-bis(3-phenylpropyl)pyrimidine-2,4,6(1H,3H,5H)-trione **2**.

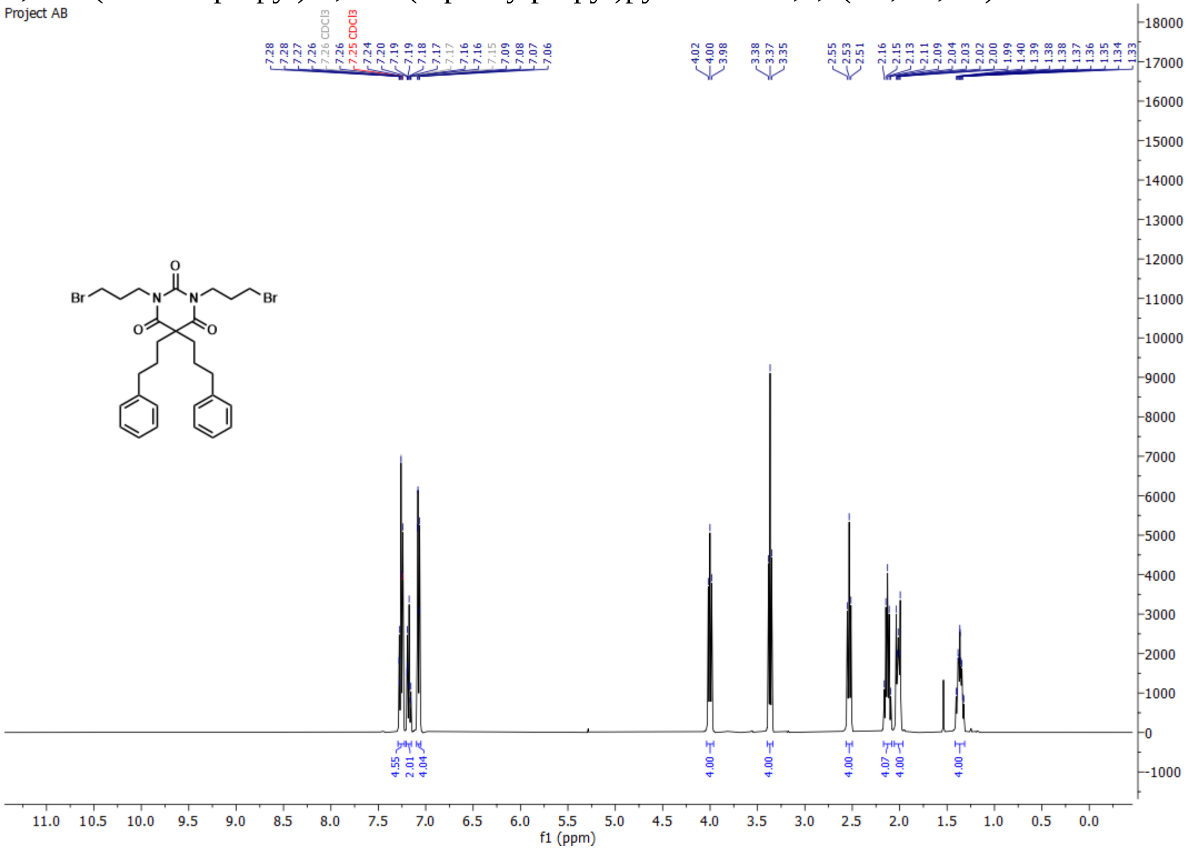

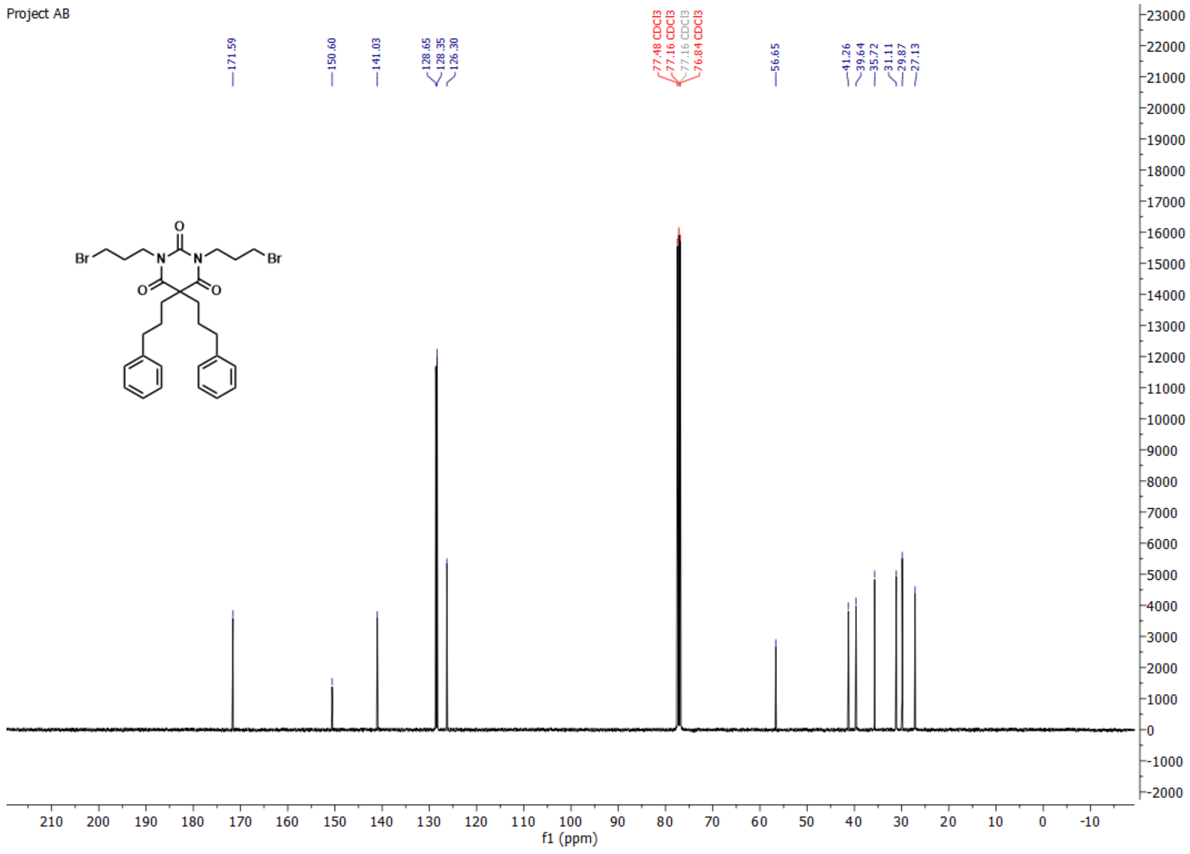
Project AB
171.59
150.60
141.03
128.65
128.35
126.30
77.48 CDCl3
77.16 CDCl3
77.16 CDCl3
76.84 CDCl3
56.65
41.26
39.64
35.72
31.11
29.87
27.13
Br
N
O
Br
f1 (ppm)

1,3-bis(4-bromobutyl)-5,5-bis(3-phenylpropyl)pyrimidine-2,4,6(1H,3H,5H)-trione **3**.

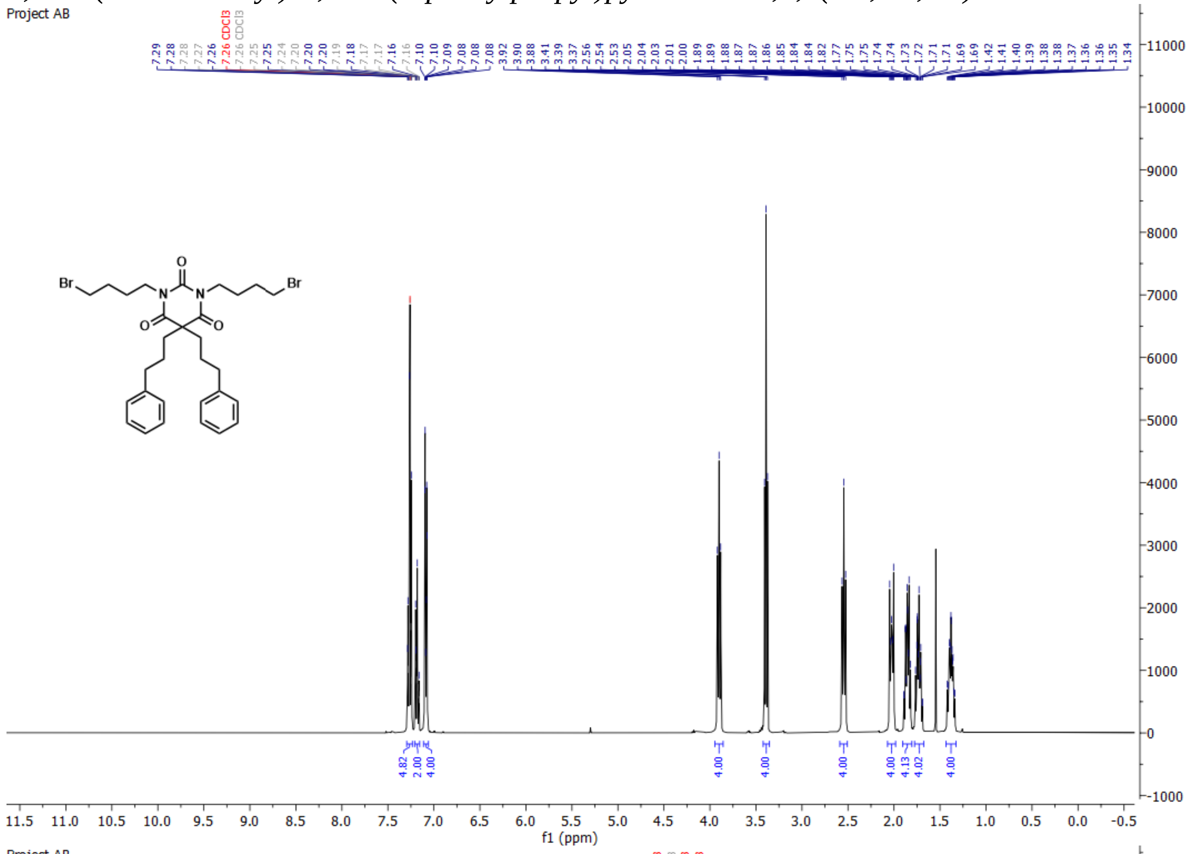

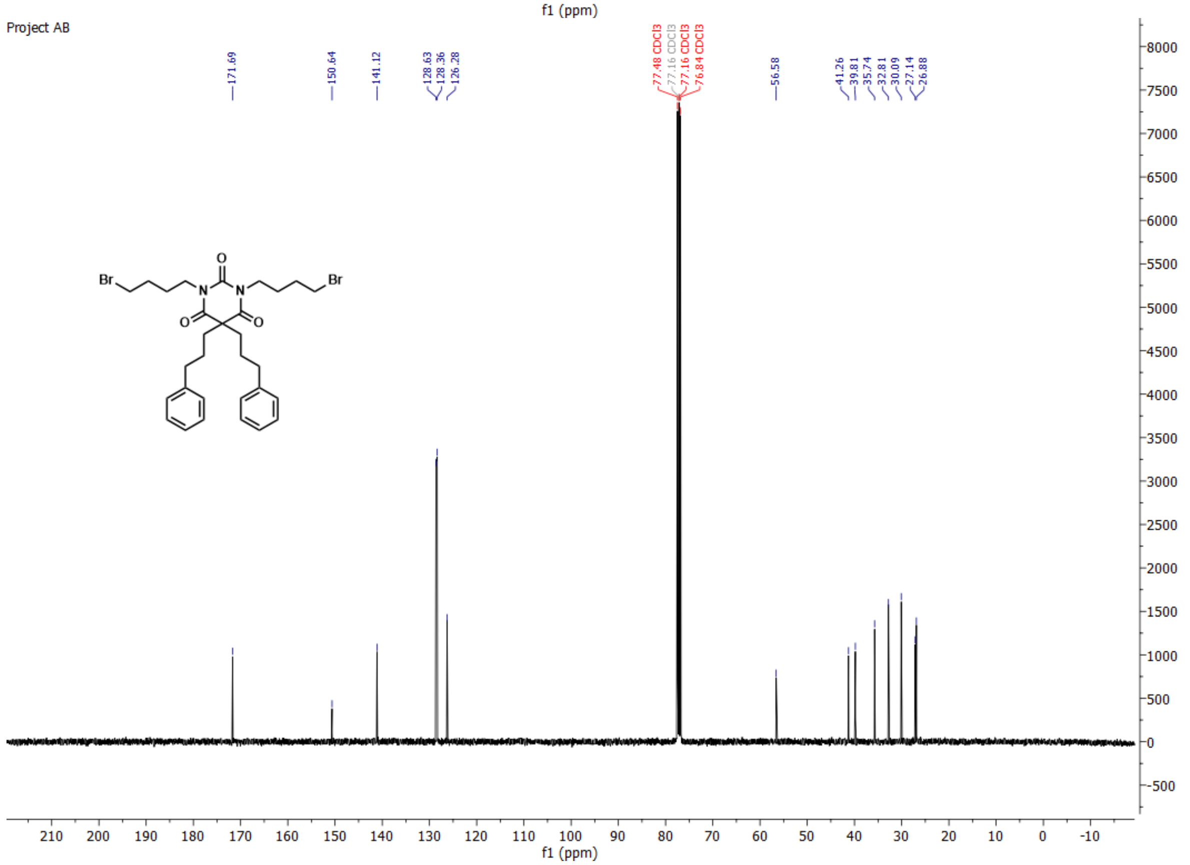

2.2 Final MPMs

1,3-bis(2-aminoethyl)-5,5-bis(3-phenylpropyl)pyrimidine-2,4,6(1H,3H,5H)-trione **MPM-2:0**.

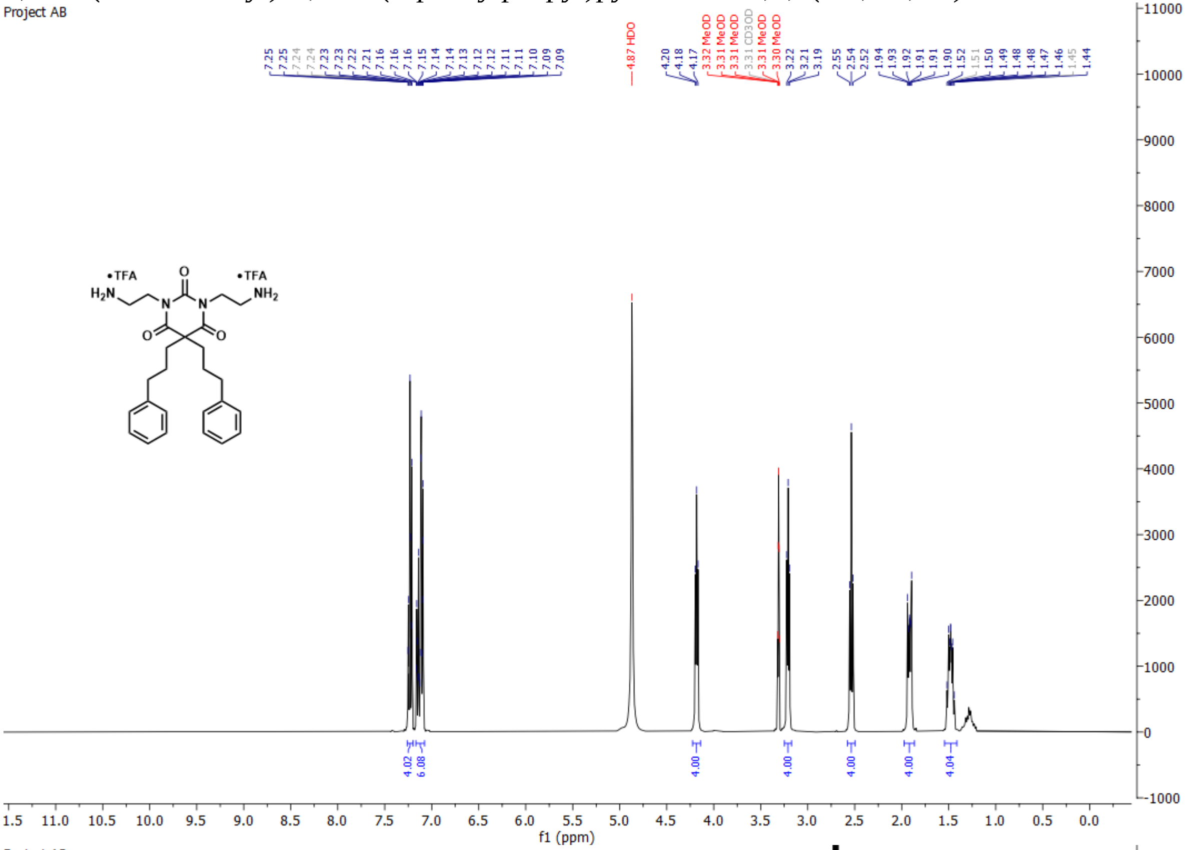

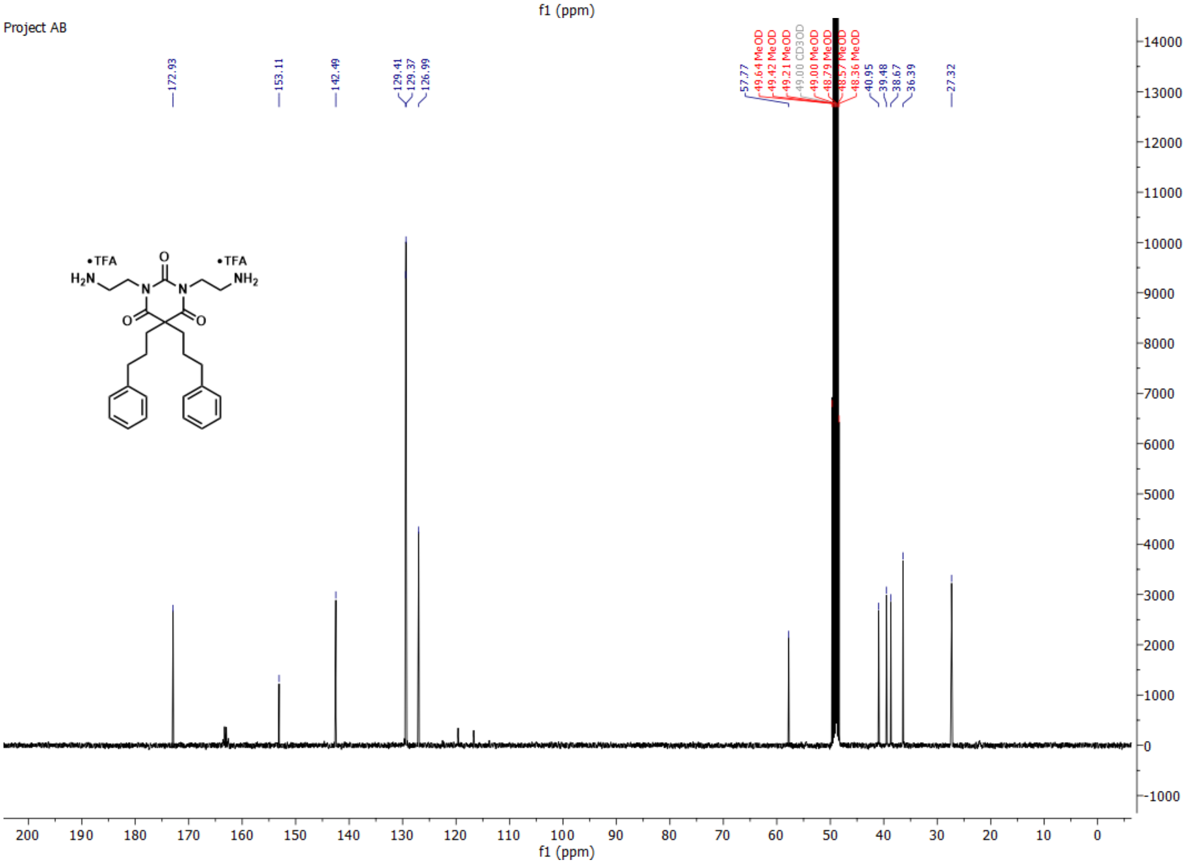

1,3-bis(3-aminopropyl)-5,5-bis(3-phenylpropyl)pyrimidine-2,4,6(1H,3H,5H)-trione **MPM-3:0**.

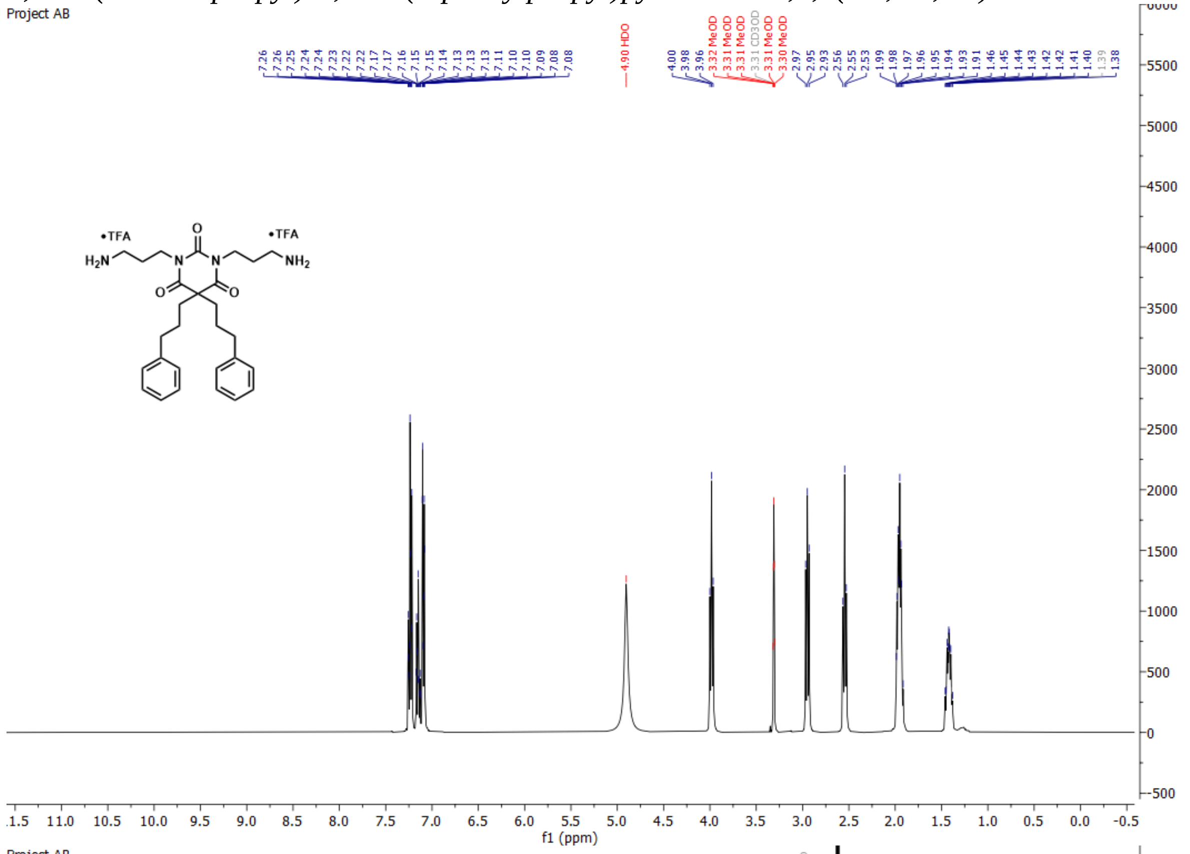

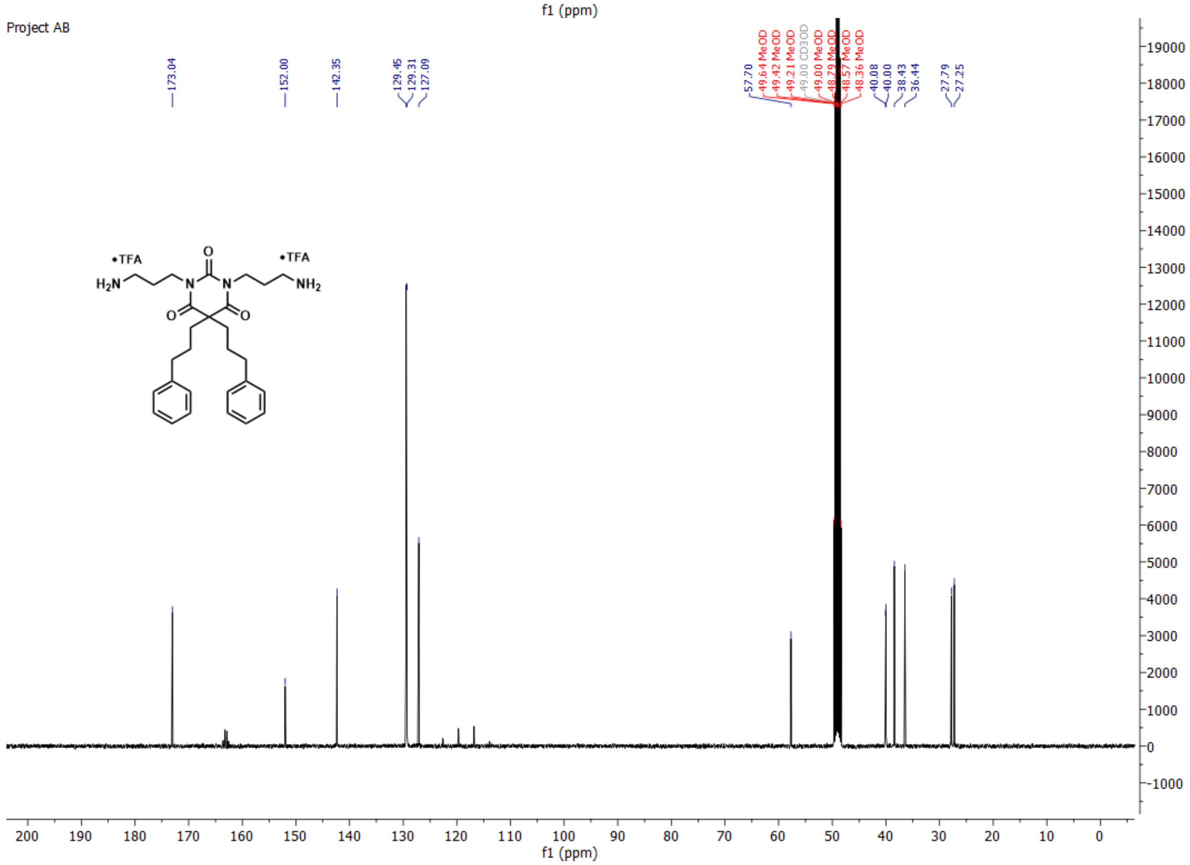

1,3-bis(4-aminobutyl)-5,5-bis(3-phenylpropyl)pyrimidine-2,4,6(1H,3H,5H)-trione **MPM-1**.

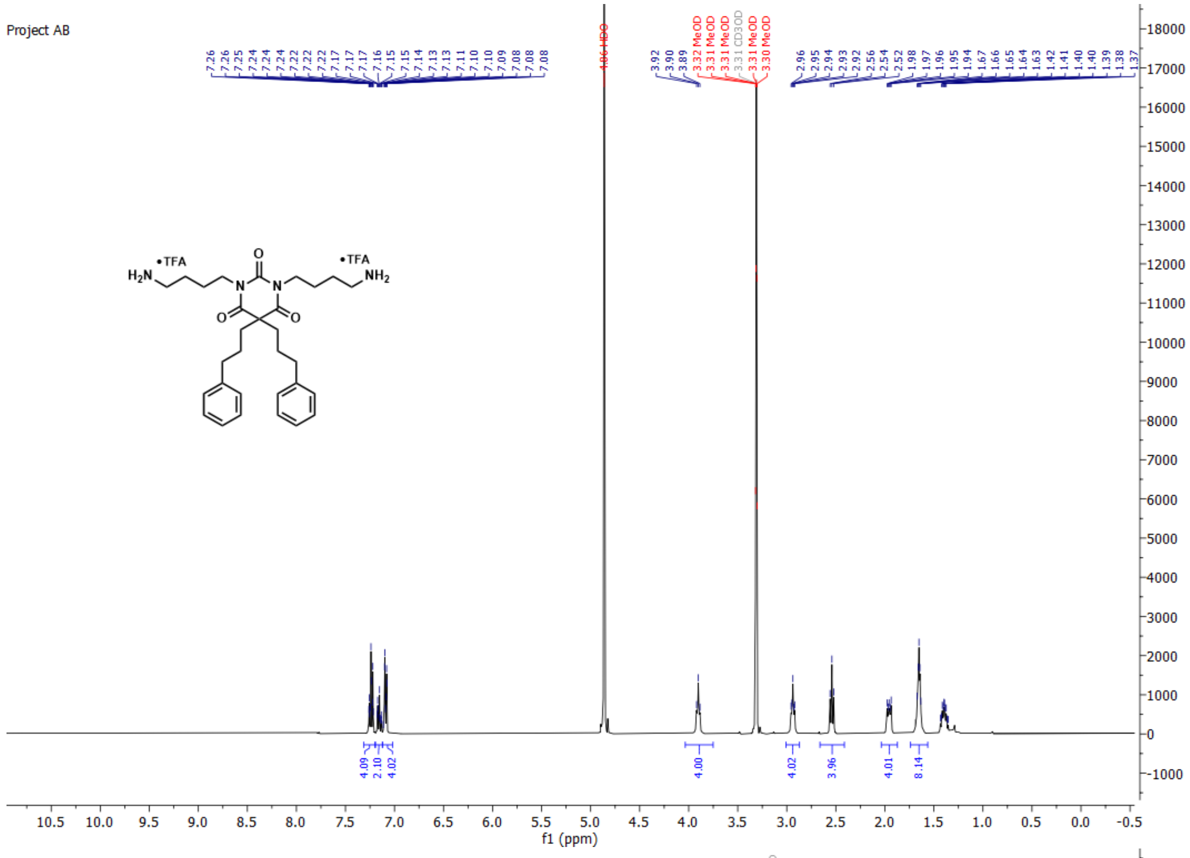

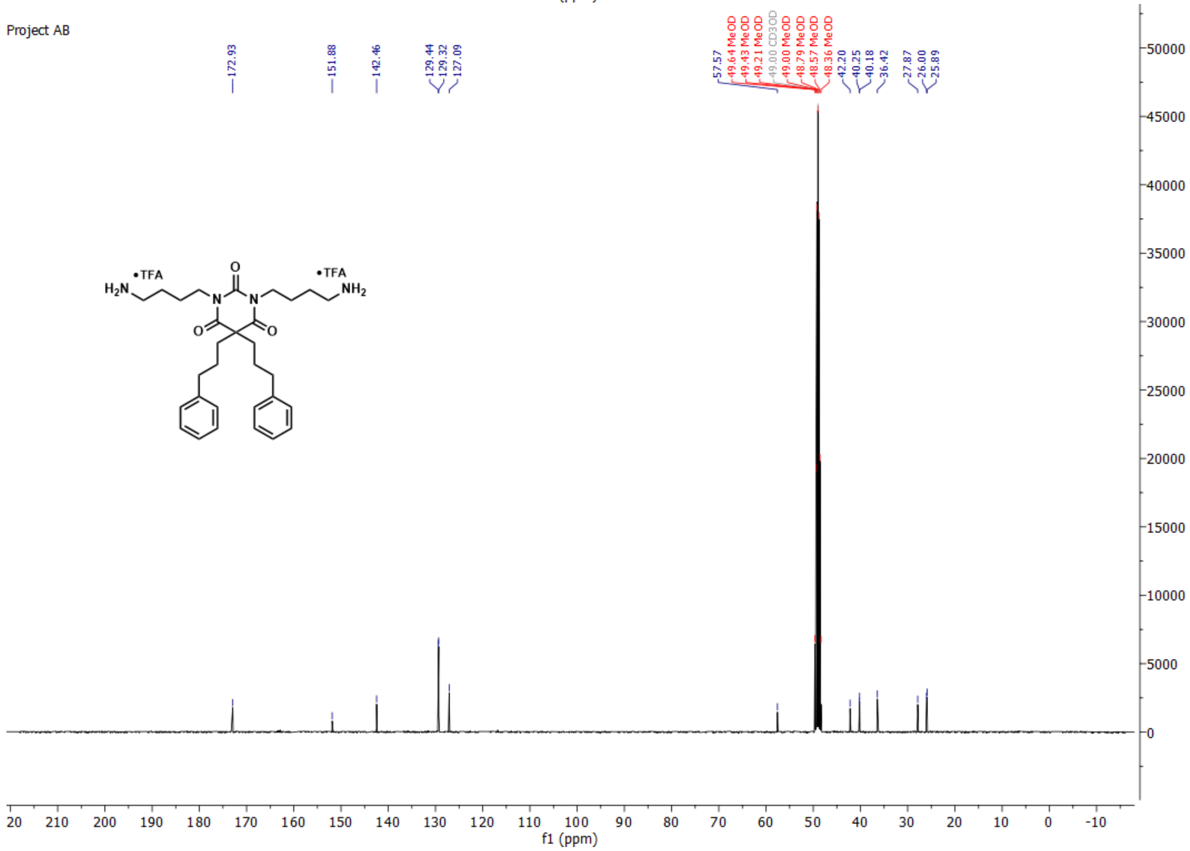

1,3-bis(5-aminopentyl)-5,5-bis(3-phenylpropyl)pyrimidine-2,4,6(1H,3H,5H)-trione **MPM-5:0**.

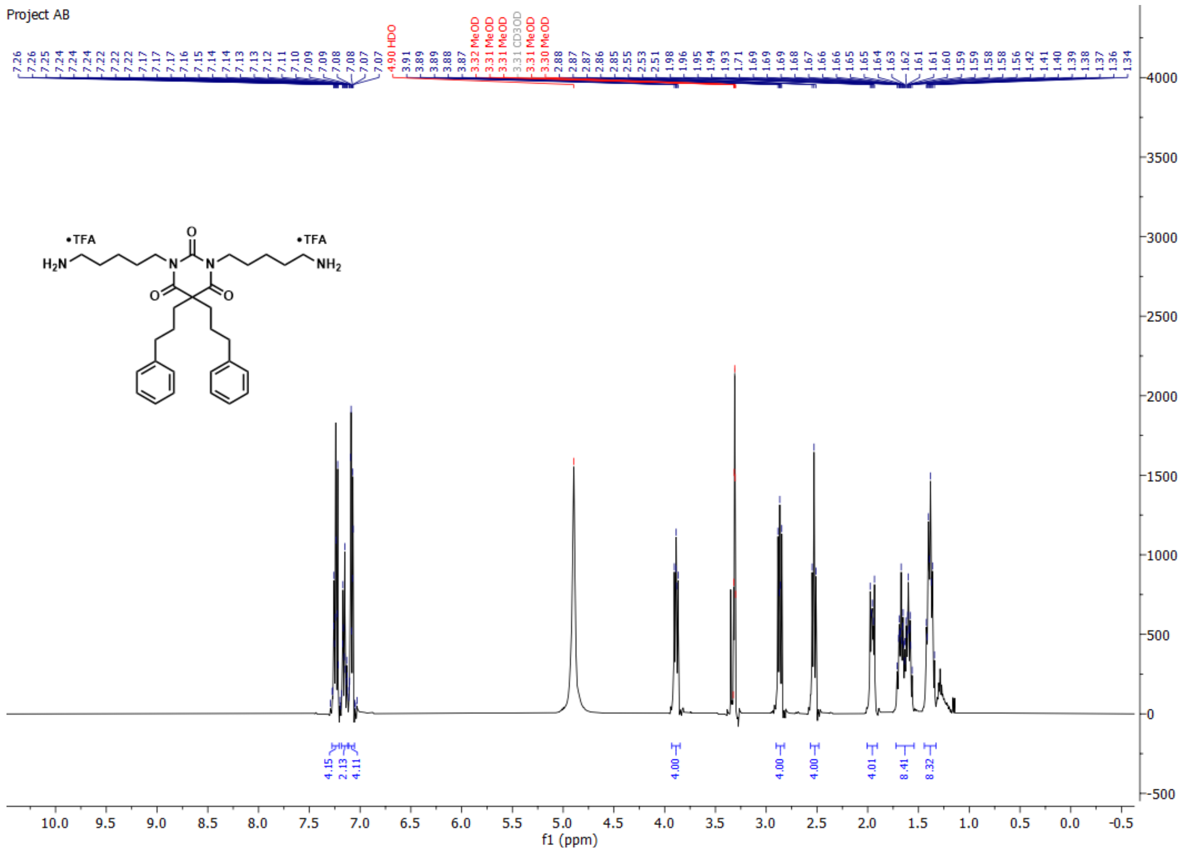

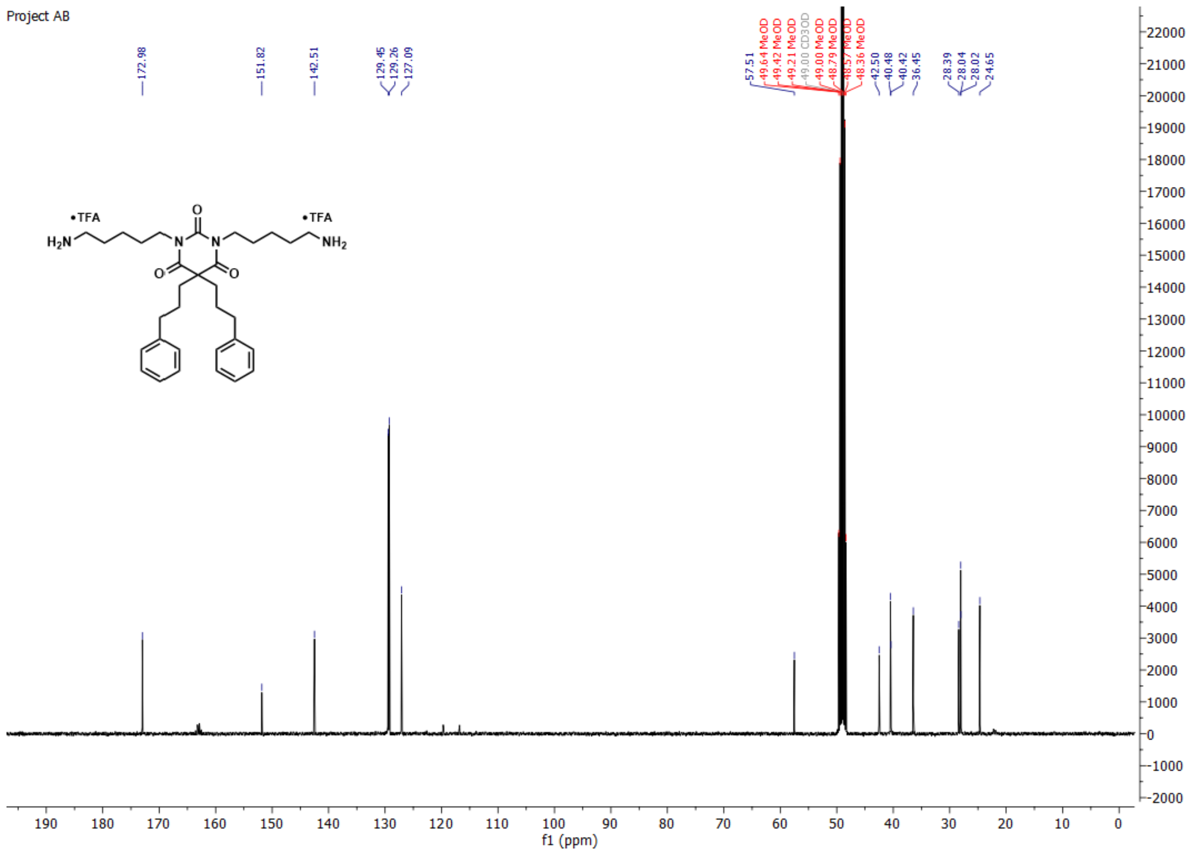

1,3-bis(6-aminohexyl)-5,5-bis(3-phenylpropyl)pyrimidine-2,4,6(1H,3H,5H)-trione **MPM-6:0**.

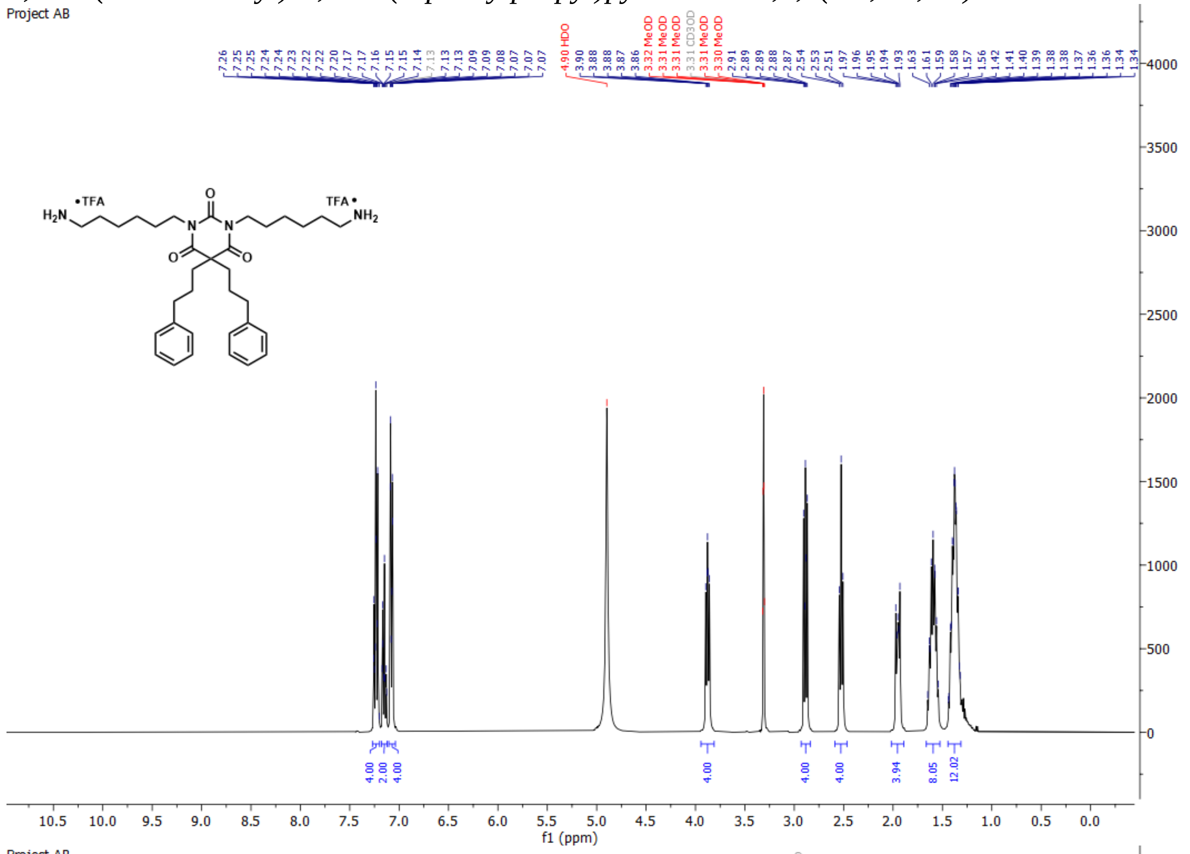

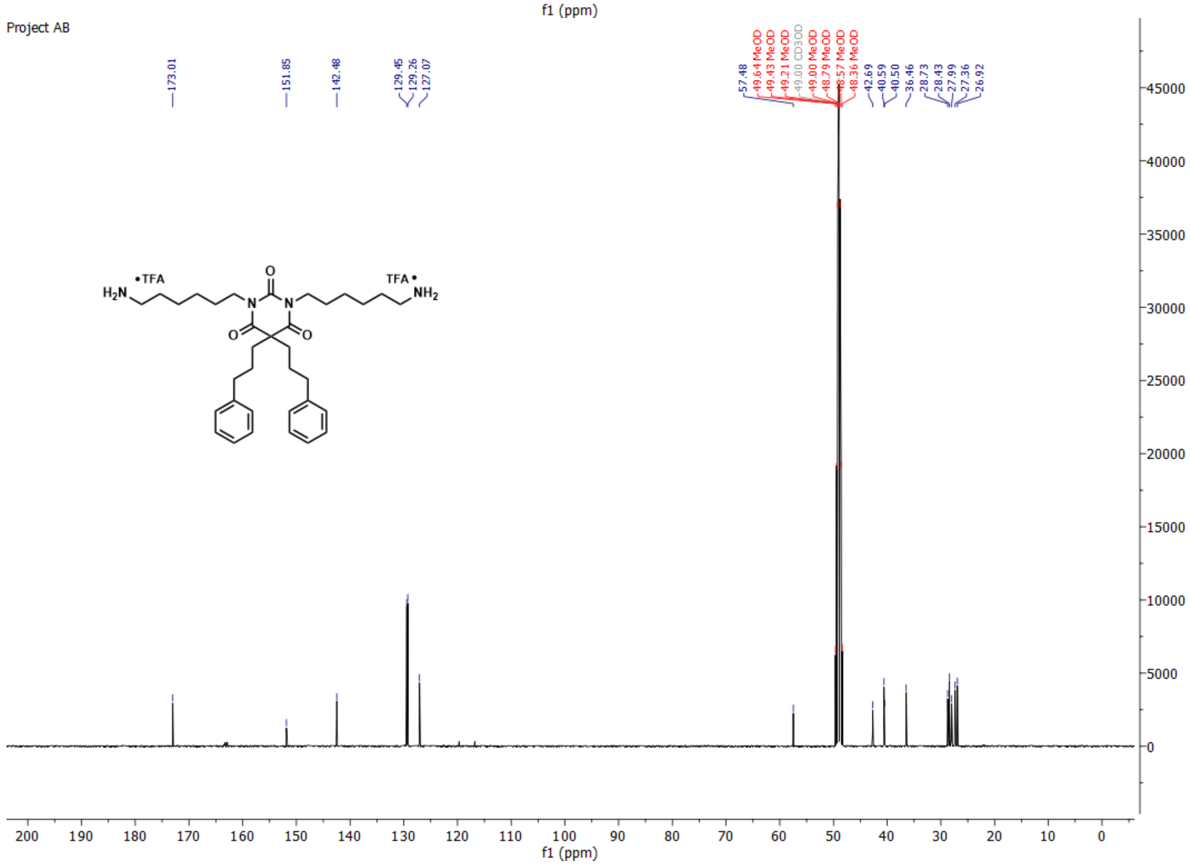

1,3-bis(4-(methylamino)butyl)-5,5-bis(3-phenylpropyl)pyrimidine-2,4,6(1H,3H,5H)-trione **MPM-4:1**.

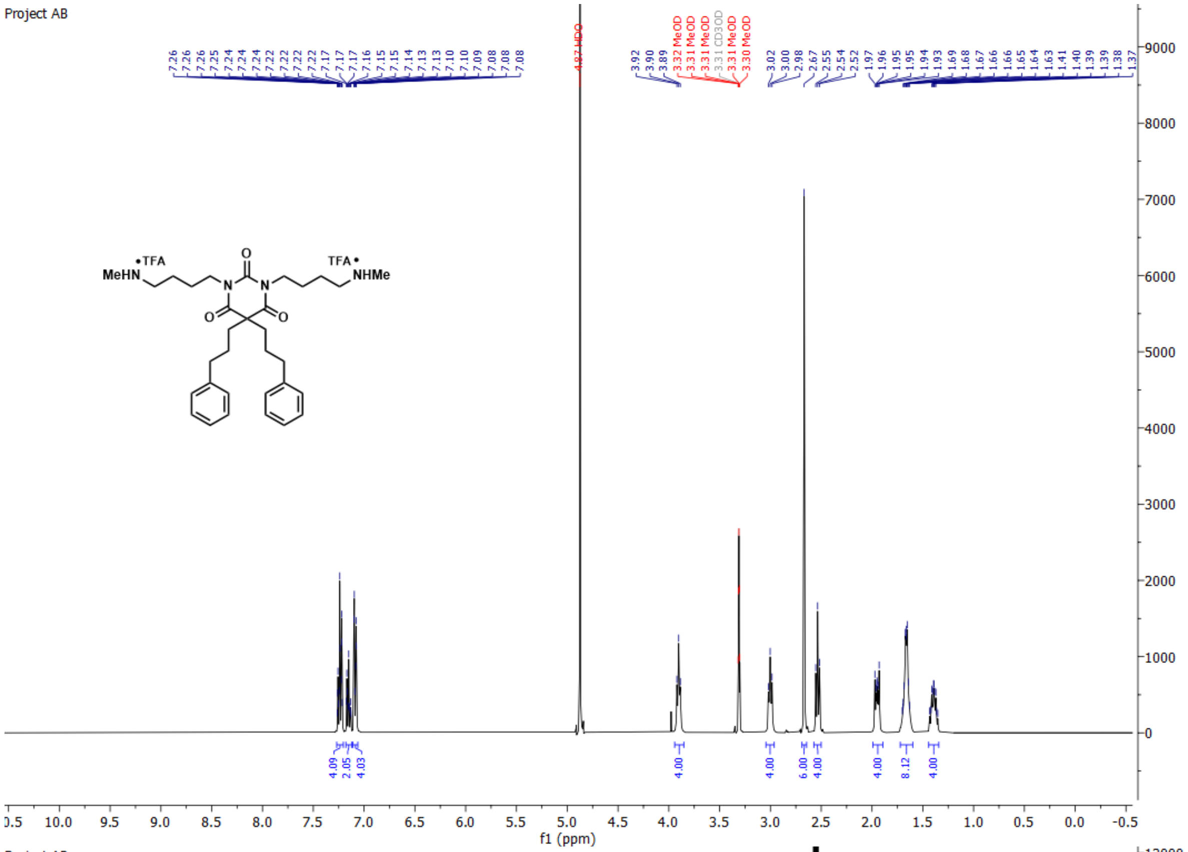

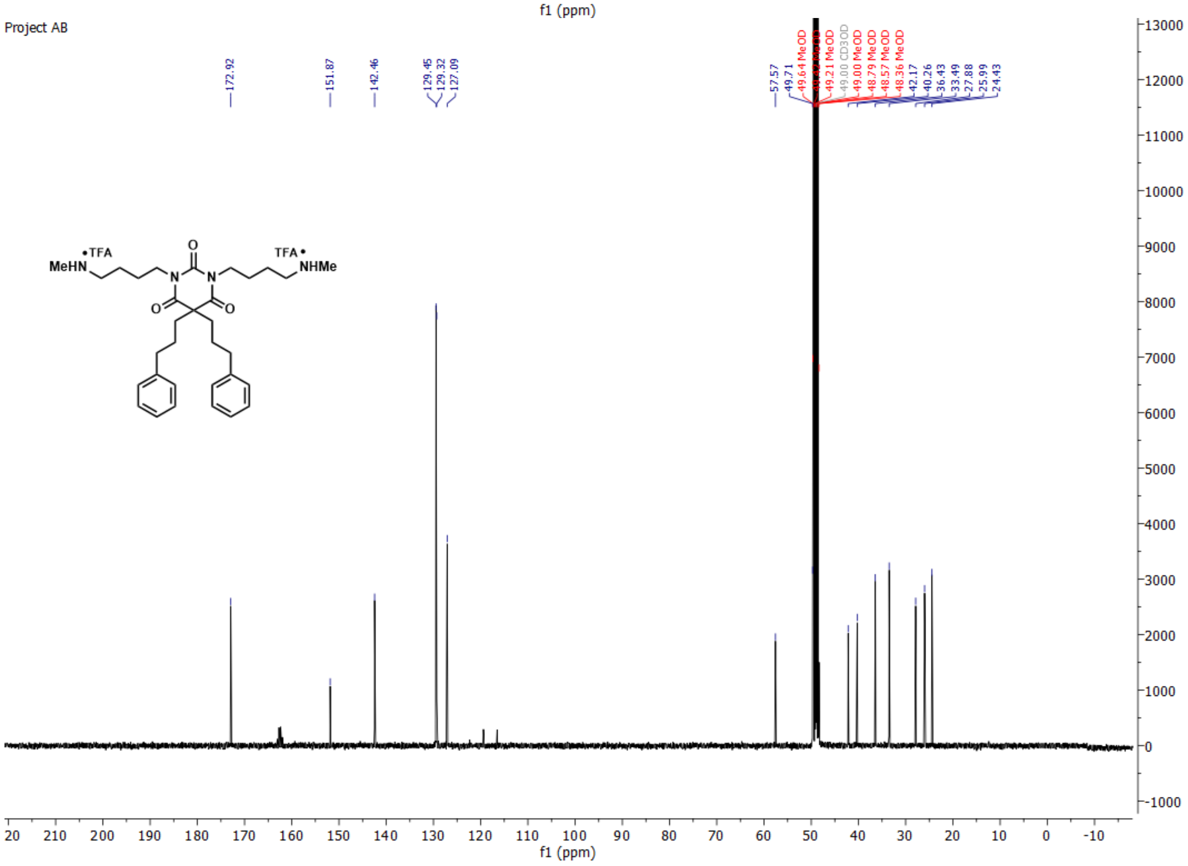

1,3-bis(4-(dimethylamino)butyl)-5,5-bis(3-phenylpropyl)pyrimidine-2,4,6(1H,3H,5H)-trione **MPM-4:2**.

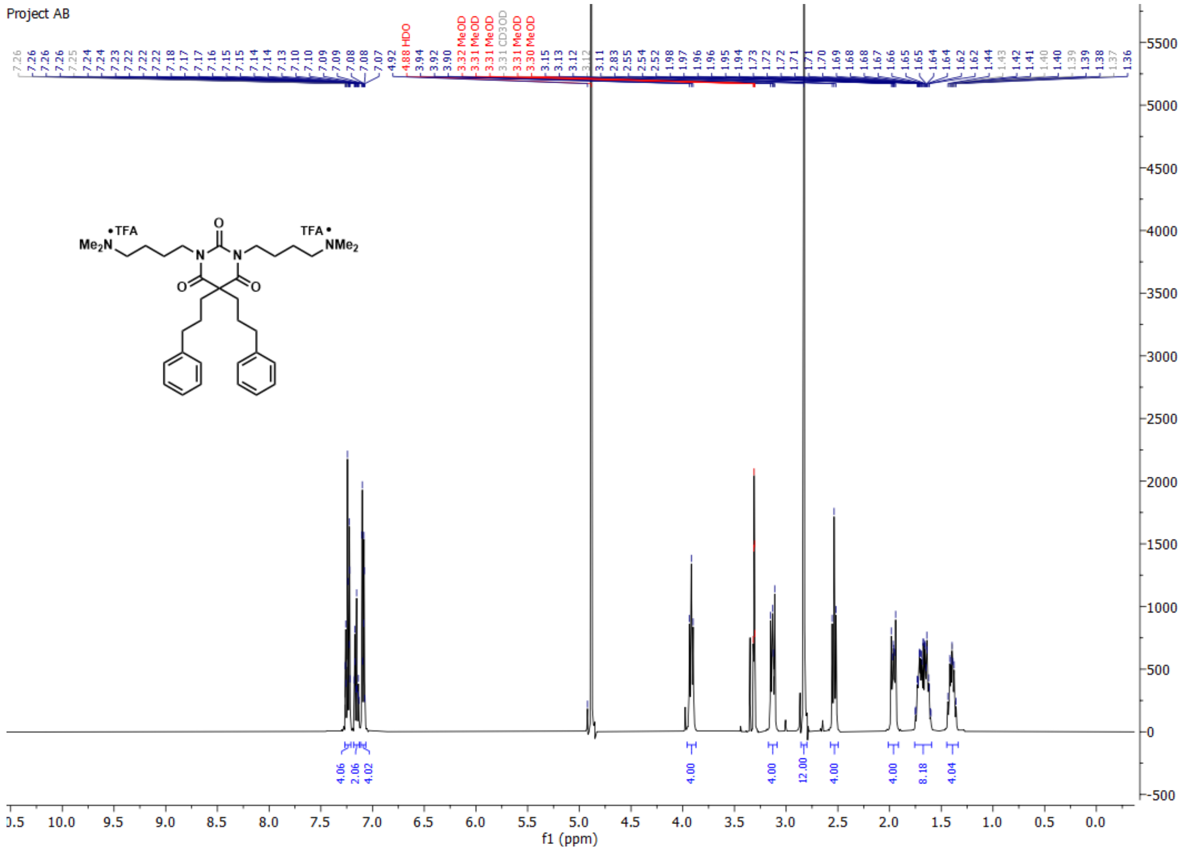

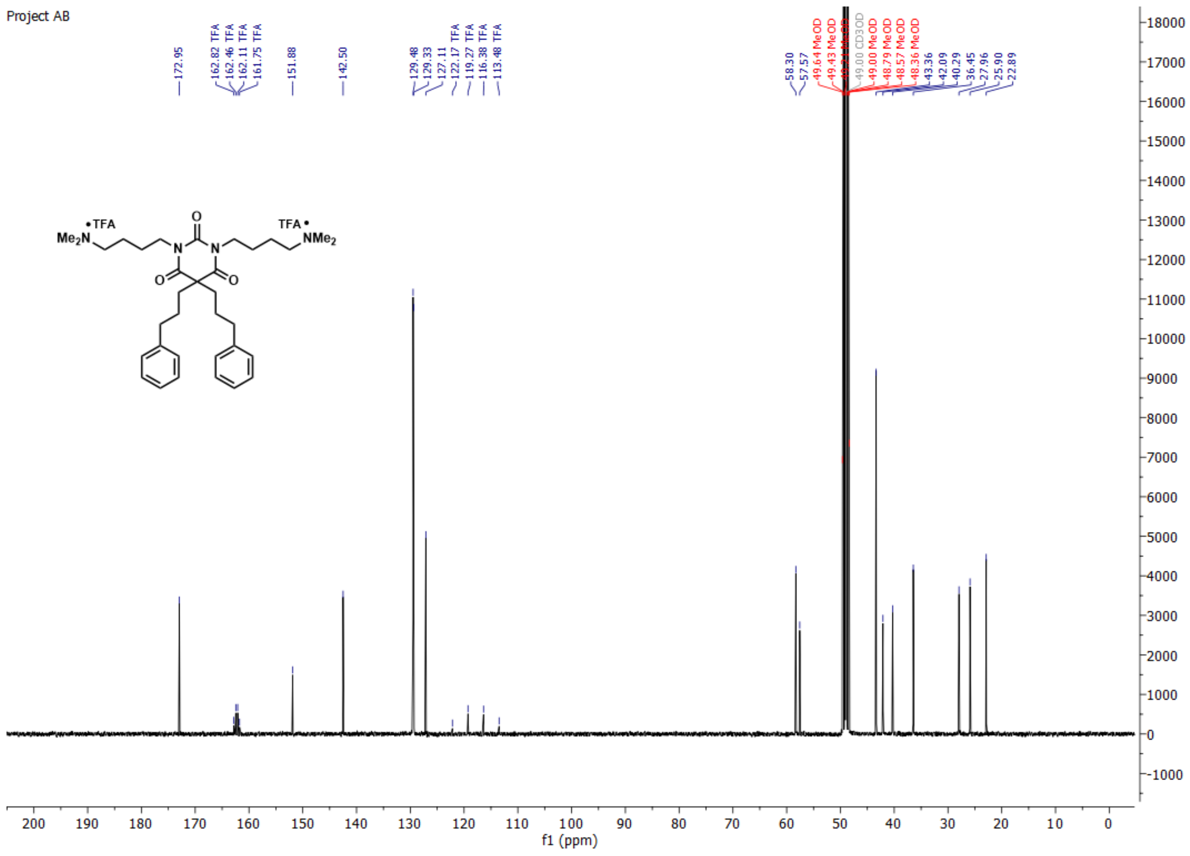
Project AB
172.95
162.82 TFA
162.46 TFA
162.11 TFA
161.75 TFA
151.88
142.50
129.48
129.33
127.11
122.17 TFA
119.27 TFA
116.38 TFA
113.48 TFA
58.30
57.57
49.64 MeOD
49.43 MeOD
49.00 CD3OD
49.00 MeOD
48.79 MeOD
48.57 MeOD
48.36 MeOD
43.36
42.09
40.29
36.45
27.96
25.90
22.89
•TFA
Me2N
TFA•
NMe2
f1 (ppm)

4,4'-(2,4,6-trioxo-5,5-bis(3-phenylpropyl)dihydropyrimi-dine-1,3(2H,4H)-diyl)bis(N,N,N-trimethylbutan-1-aminium) **MPM-4:3**.

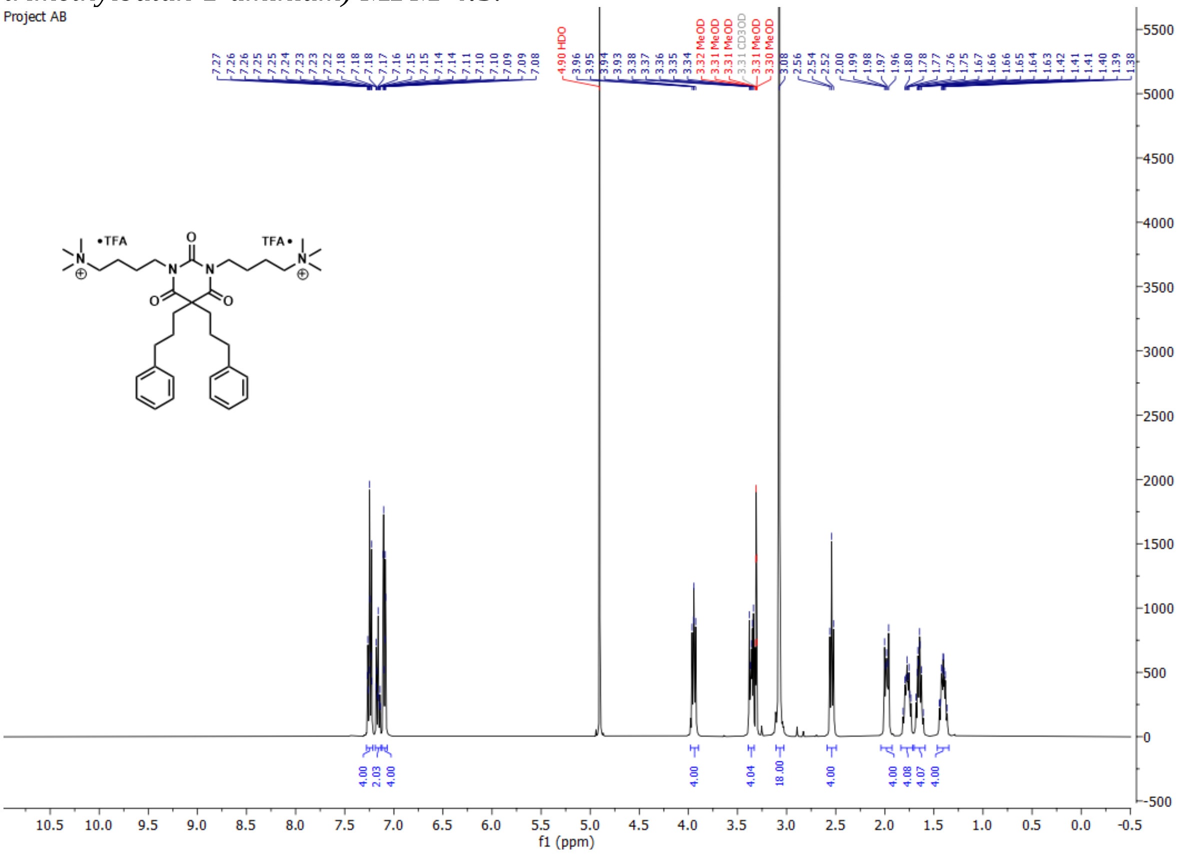

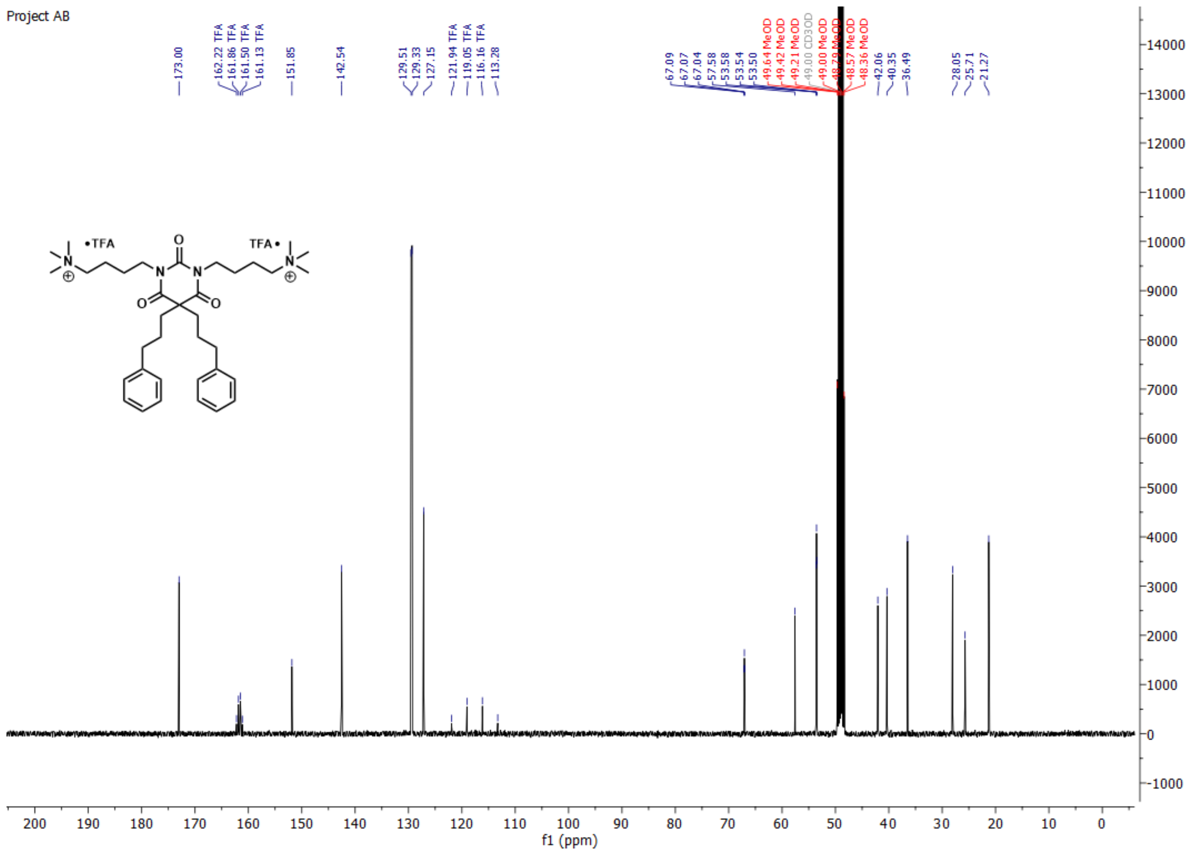

Project AB
173.00
162.22 TFA
161.86 TFA
161.50 TFA
161.13 TFA
151.85
142.54
129.51
129.33
127.15
121.94 TFA
119.05 TFA
116.16 TFA
113.28
67.09
67.07
67.04
57.58
53.58
53.54
53.50
49.64 MeOD
49.42 MeOD
49.21 MeOD
49.00 CD3OD
49.00 MeOD
48.79 MeOD
48.57 MeOD
48.36 MeOD
42.06
40.35
36.49
28.05
25.71
21.27
•TFA
TFA•
f1 (ppm)

1,3-bis(3-(dimethylamino)propyl)-5,5-bis(3-phenylpropyl)-pyrimidine-2,4,6(1H,3H,5H)-trione **MPM-3:2**.

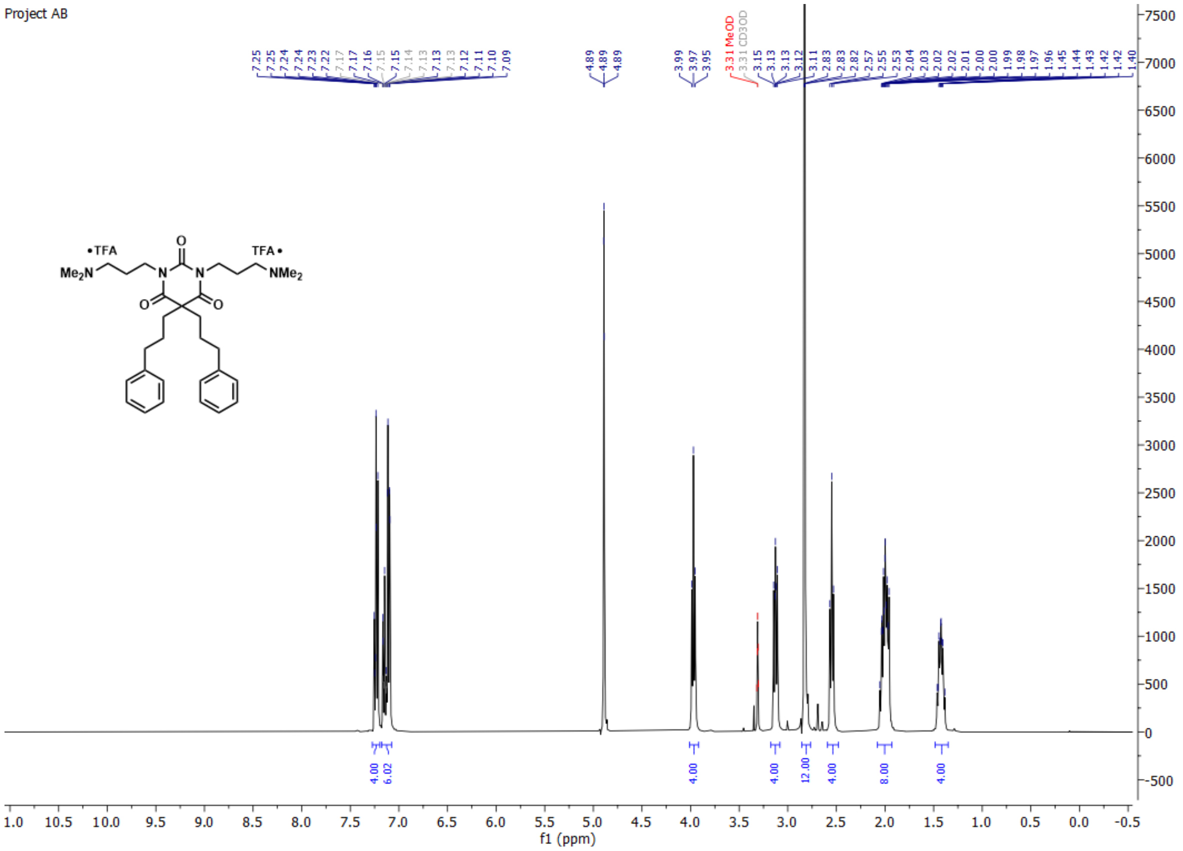

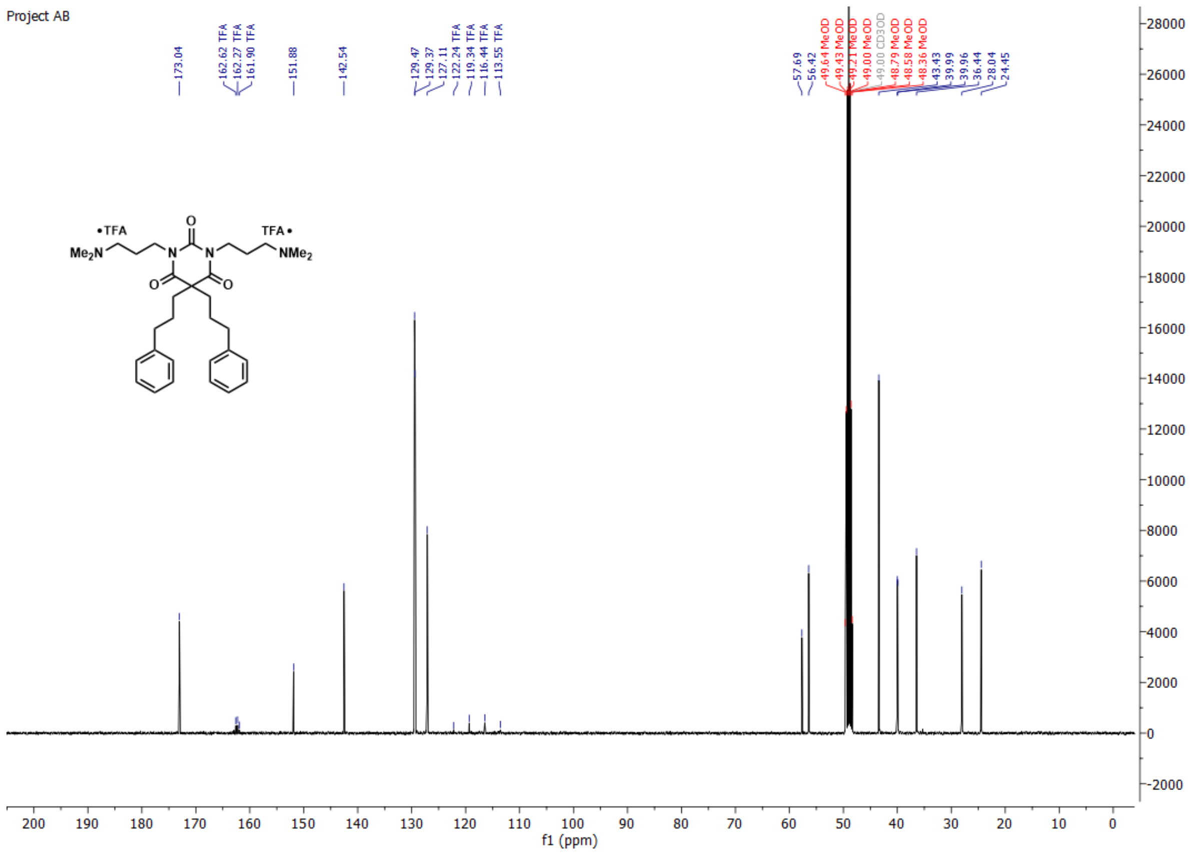
Project AB
173.04
162.62 TFA
162.27 TFA
161.90 TFA
151.88
142.54
129.47
129.37
127.11
122.24 TFA
119.34 TFA
116.44 TFA
113.55 TFA
57.69
56.42
49.64 MeOD
49.43 MeOD
49.21 MeOD
49.00 MeOD
49.00 CD3OD
48.79 MeOD
48.58 MeOD
48.36 MeOD
43.43
39.99
39.96
36.44
28.04
24.45
•TFA
TFA•
Me_2N
NMe_2
f1 (ppm)

1,3-bis(2-(dimethylamino)ethyl)-5,5-bis(3-phenylpropyl)pyrimidine-2,4,6(1H,3H,5H)-trione **MPM-2:2**.

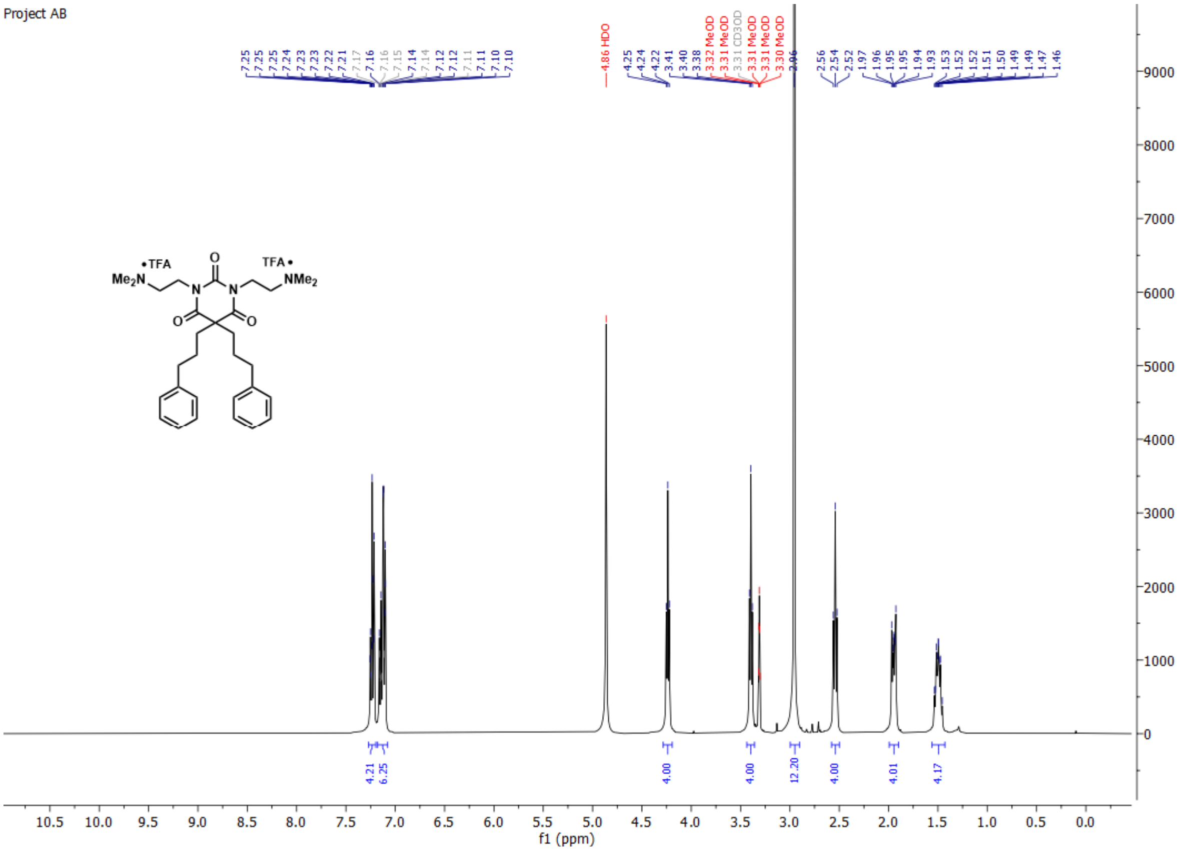

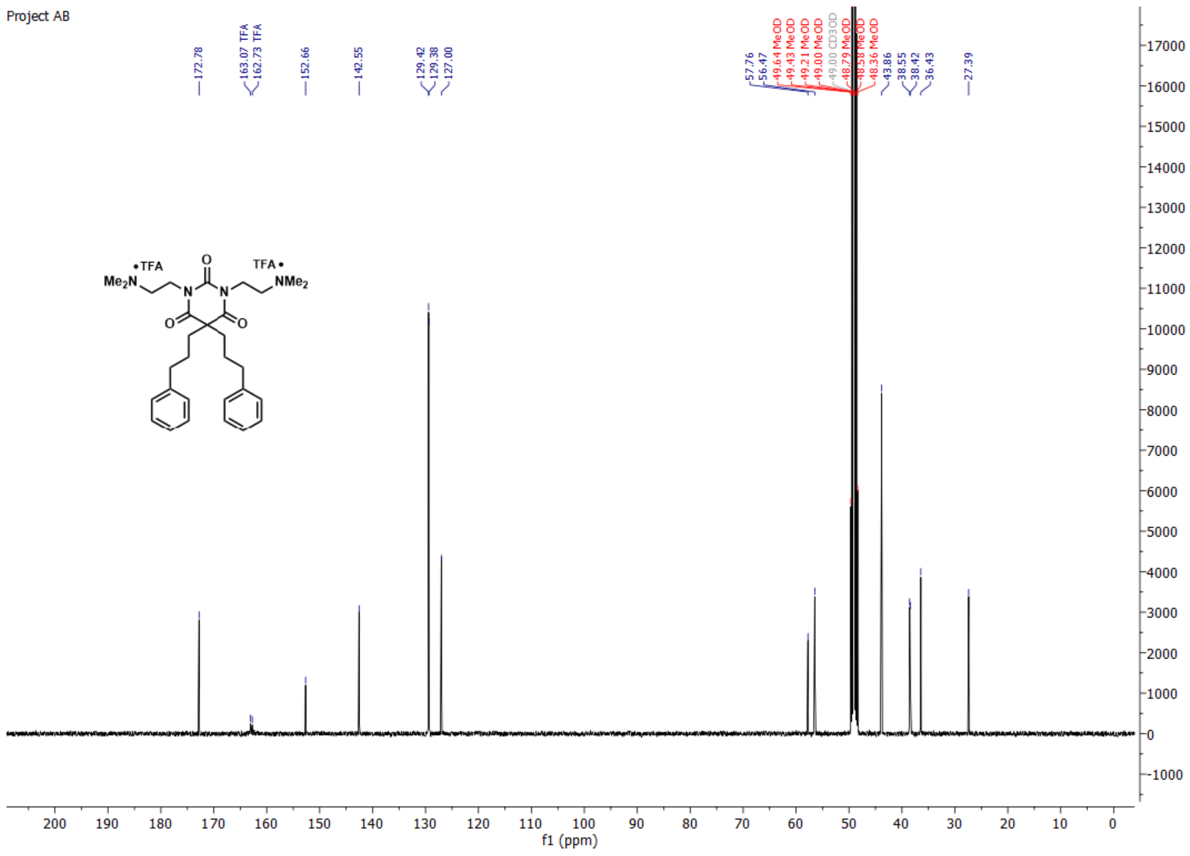

Project AB
172.78
163.07 TFA
162.73 TFA
152.66
142.55
129.42
129.38
127.00
57.76
56.47
49.64 MeOD
49.43 MeOD
49.21 MeOD
49.00 MeOD
49.00 CD3OD
48.79 MeOD
48.58 MeOD
48.36 MeOD
43.86
38.55
38.42
36.43
27.39
•TFA
TFA•
Me2N
NMe2
f1 (ppm)

3 SFC traces

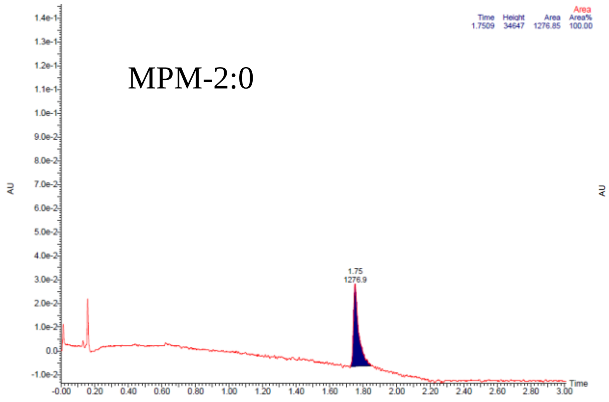
MPM-2:0
1.75
1276.9

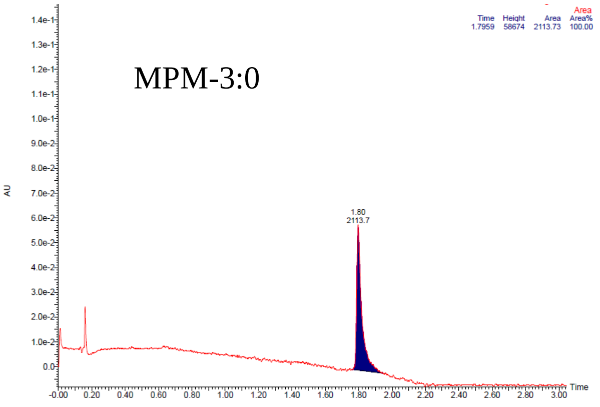
MPM-3:0
1.80
2113.7

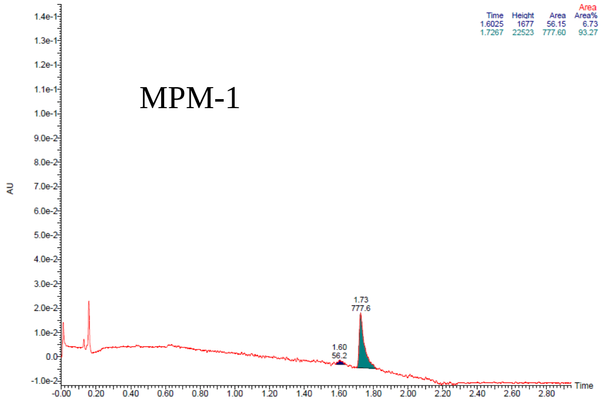
MPM-1
1.73
777.6
1.60
56.2

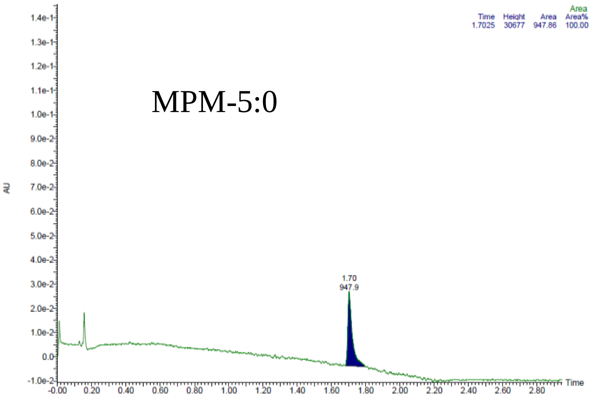
MPM-5:0
1.70
947.9

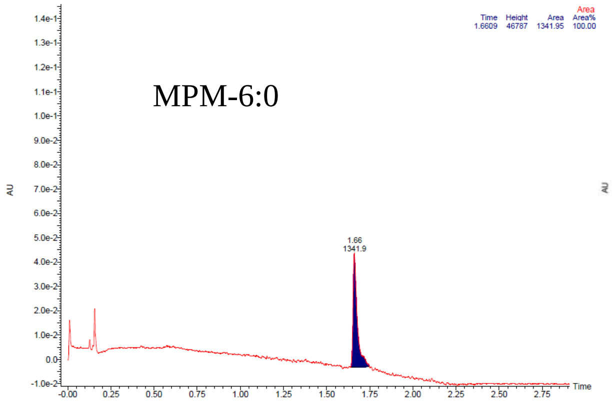
MPM-6:0
1.66
1341.9

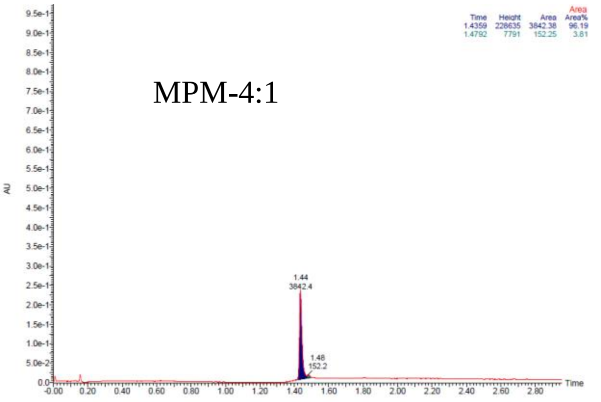
MPM-4:1
1.44
3842.4
1.48
152.2

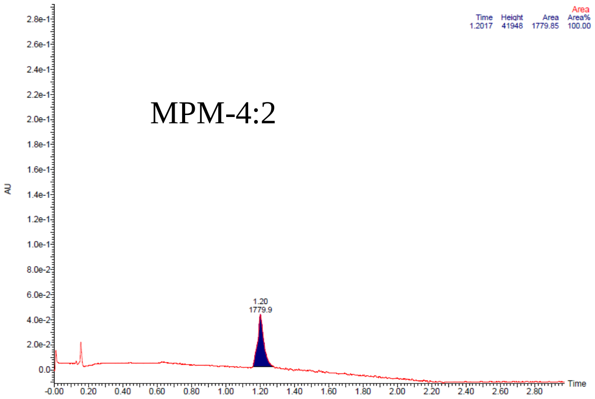

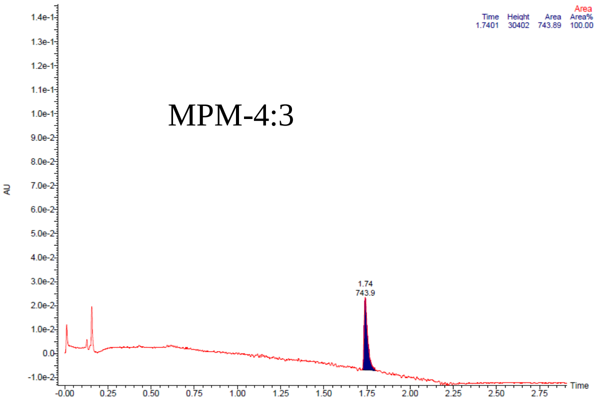

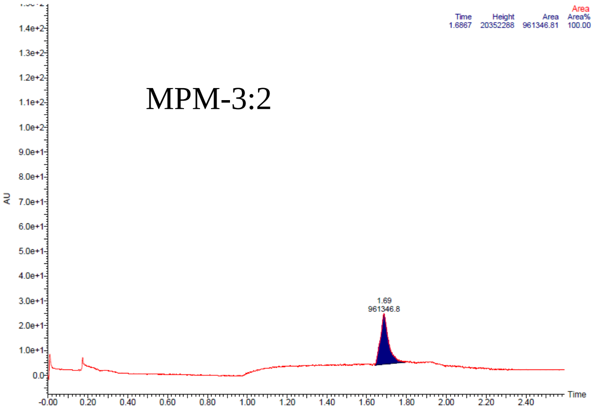

4 References

(1) von Hofsten, S.; Paulsen, M. H.; Magnussen, S. N.; Ausbacher, D.; Kranz, M.; Bayer, A.; Strøm, M. B.; Berge, G. The marine natural product mimic MPM-1 is cytolytic and induces DAMP release from human cancer cell lines. *Sci. Rep.* **2022**, *12* (1), 1-15.
